# Supplementary material for: Comparative genomics analysis of three conserved plasmid families in the Western Hemisphere soft tick-borne relapsing fever borreliae provides insight into variation in genome structure and antigenic variation systems
Source: bioRxiv. 2023 Mar 6:2023.03.06.531354. Preprint. [Version 1] doi: 10.1101/2023.03.06.531354 (PMC10028826; doi:10.1101/2023.03.06.531354)
Supplement: Supplement 2 [file media-2.pdf]

This file contains a sheet for each of the 5 repetitive blocks. The sheets contain the gene loci found in each of the 5 repetitive blocks for all the isolates analyzed.

Block A	locus	PFAM/SSF Designation by InterProScan	Description
B. anserina **BA2**	baBA2 000918	PF05714	Borrelia Bbcrasp-1 domain containing protein
	baBA2 000919	PF05714	Borrelia Bbcrasp-1 domain containing protein
	baBA2 000920	PF05714	Borrelia Bbcrasp-1 domain containing protein
	baBA2 000921	PF05714	Borrelia Bbcrasp-1 domain containing protein
	baBA2 000922	PF05714	Borrelia Bbcrasp-1 domain containing protein
	baBA2 000923	PF05714	Borrelia Bbcrasp-1 domain containing protein
B. hermsii **DAH**	bhDAH 001156	PF05714	Borrelia Bbcrasp-1 domain containing protein
	bhDAH 001157	PF05714	Borrelia Bbcrasp-1 domain containing protein
	bhDAH 001158	PF05714	Borrelia Bbcrasp-1 domain containing protein
	bhDAH 001159	PF05714	Borrelia Bbcrasp-1 domain containing protein
	bhDAH 001160	PF05714	Borrelia Bbcrasp-1 domain containing protein
	bhDAH 001161	PF05714	Borrelia Bbcrasp-1 domain containing protein
	bhDAH 001162	PF05714	Borrelia Bbcrasp-1 domain containing protein
	bhDAH 001163	PF05714	Borrelia Bbcrasp-1 domain containing protein
	bhDAH 001164	PF05714	Borrelia Bbcrasp-1 domain containing protein
	bhDAH 001165	PF05714	Borrelia Bbcrasp-1 domain containing protein
	bhDAH 001166	PF05714	Borrelia Bbcrasp-1 domain containing protein
	bhDAH 001167	PF05714	Borrelia Bbcrasp-1 domain containing protein
	bhDAH 001168	not classified by interproscan	
	bhDAH 001169	PF05714	Borrelia Bbcrasp-1 domain containing protein
	bhDAH 001170	PF05714	Borrelia Bbcrasp-1 domain containing protein
	bhDAH 001171	PF05714	Borrelia Bbcrasp-1 domain containing protein
	bhDAH 001172	PF05714	Borrelia Bbcrasp-1 domain containing protein
	bhDAH 001173	PF05714	Borrelia Bbcrasp-1 domain containing protein
	bhDAH 001174	PF05714	Borrelia Bbcrasp-1 domain containing protein
	bhDAH 001175	PF05714	Borrelia Bbcrasp-1 domain containing protein
	bhDAH 001176	PF05714	Borrelia Bbcrasp-1 domain containing protein
	bhDAH 001177	PF05714	Borrelia Bbcrasp-1 domain containing protein
	bhDAH 001178	PF05714	Borrelia Bbcrasp-1 domain containing protein
	bhDAH 001179	PF05714	Borrelia Bbcrasp-1 domain containing protein
	bhDAH 001180	PF05714	Borrelia Bbcrasp-1 domain containing protein
	bhDAH 001181	PF05714	Borrelia Bbcrasp-1 domain containing protein
B. hermsii **YOR**	bhYOR 001068	PF05714	Borrelia Bbcrasp-1 domain containing protein
	bhYOR 001069	PF05714	Borrelia Bbcrasp-1 domain containing protein
	bhYOR 001070	PF05714	Borrelia Bbcrasp-1 domain containing protein
	bhYOR 001071	PF05714	Borrelia Bbcrasp-1 domain containing protein
	bhYOR 001072	PF05714	Borrelia Bbcrasp-1 domain containing protein
	bhYOR 001073	PF05714	Borrelia Bbcrasp-1 domain containing protein
	bhYOR 001074	PF05714	Borrelia Bbcrasp-1 domain containing protein
	bhYOR 001075	PF05714	Borrelia Bbcrasp-1 domain containing protein
	bhYOR 001076	not classified by interproscan	
	bhYOR 001077	not classified by interproscan	
	bhYOR 001078	PF05714	Borrelia Bbcrasp-1 domain containing protein
	bhYOR 001079	not classified by interproscan	
	bhYOR 001080	not classified by interproscan	
	bhYOR 001081	PF05714	Borrelia Bbcrasp-1 domain containing protein
	bhYOR 001082	PF05714	Borrelia Bbcrasp-1 domain containing protein
	bhYOR 001083	PF05714	Borrelia Bbcrasp-1 domain containing protein
	bhYOR 001084	PF05714	Borrelia Bbcrasp-1 domain containing protein
	bhYOR 001085	PF05714	Borrelia Bbcrasp-1 domain containing protein
	bhYOR 001086	PF05714	Borrelia Bbcrasp-1 domain containing protein
	bhYOR 001087	SSF51126	pectin lyase-like fold
	bhYOR 001088	PF05714	Borrelia Bbcrasp-1 domain containing protein
	bhYOR 001089	not classified by interproscan	
	bhYOR 001090	not classified by interproscan	
	bhYOR 001091	PF05714	Borrelia Bbcrasp-1 domain containing protein
	bhYOR 001092	not classified by interproscan	
	bhYOR 001093	PF05714	Borrelia Bbcrasp-1 domain containing protein
	bhYOR 001094	PF05714	Borrelia Bbcrasp-1 domain containing protein
	bhYOR 001095	PF05714	Borrelia Bbcrasp-1 domain containing protein
	bhYOR 001096	PF05714	Borrelia Bbcrasp-1 domain containing protein
	bhYOR 001097	PF05714	Borrelia Bbcrasp-1 domain containing protein
	bhYOR 001098	PF05714	Borrelia Bbcrasp-1 domain containing protein
	bhYOR 001099	PF05714	Borrelia Bbcrasp-1 domain containing protein
	bhYOR 001100	PF05714	Borrelia Bbcrasp-1 domain containing protein
	bhYOR 001101	PF05714	Borrelia Bbcrasp-1 domain containing protein
	bhYOR 001102	PF05714	Borrelia Bbcrasp-1 domain containing protein
	bhYOR 001103	PF05714	Borrelia Bbcrasp-1 domain containing protein
	bhYOR 001104	PF05714	Borrelia Bbcrasp-1 domain containing protein
	bhYOR 001105	PF05714	Borrelia Bbcrasp-1 domain containing protein
B. coriaceae **Co53**	bcCo53 001122	PF05714	Borrelia Bbcrasp-1 domain containing protein
	bcCo53 001123	PF05714	Borrelia Bbcrasp-1 domain containing protein
	bcCo53 001124	PF05714	Borrelia Bbcrasp-1 domain containing protein
	bcCo53 001125	PF05714	Borrelia Bbcrasp-1 domain containing protein
	bcCo53 001126	PF05714	Borrelia Bbcrasp-1 domain containing protein
	bcCo53 001127	PF05714	Borrelia Bbcrasp-1 domain containing protein
	bcCo53 001128	PF05714	Borrelia Bbcrasp-1 domain containing protein
	bcCo53 001129	not classified by interproscan	
	bcCo53 001130	PF05714	Borrelia Bbcrasp-1 domain containing protein
	bcCo53 001131	PF05714	Borrelia Bbcrasp-1 domain containing protein
	bcCo53 001132	PF05714	Borrelia Bbcrasp-1 domain containing protein
	bcCo53 001133	PF05714	Borrelia Bbcrasp-1 domain containing protein
	bcCo53 001134	PF05714	Borrelia Bbcrasp-1 domain containing protein
	bcCo53 001135	PF05714	Borrelia Bbcrasp-1 domain containing protein
	bcCo53 001136	PF05714	Borrelia Bbcrasp-1 domain containing protein
B. puertoricensis **SUM**	bpuSUM 001474	PF05714	Borrelia Bbcrasp-1 domain containing protein
	bpuSUM 001475	PF05714	Borrelia Bbcrasp-1 domain containing protein
	bpuSUM 001476	PF05714	Borrelia Bbcrasp-1 domain containing protein
	bpuSUM 001477	PF05714	Borrelia Bbcrasp-1 domain containing protein
	bpuSUM 001478	PF05714	Borrelia Bbcrasp-1 domain containing protein
	bpuSUM 001479	PF05714	Borrelia Bbcrasp-1 domain containing protein
	bpuSUM 001480	PF05714	Borrelia Bbcrasp-1 domain containing protein
	bpuSUM 001481	PF05714	Borrelia Bbcrasp-1 domain containing protein
	bpuSUM 001482	PF05714	Borrelia Bbcrasp-1 domain containing protein
	bpuSUM 001483	PF05714	Borrelia Bbcrasp-1 domain containing protein
	bpuSUM 001484	PF05714	Borrelia Bbcrasp-1 domain containing protein
	bpuSUM 001485	PF05714	Borrelia Bbcrasp-1 domain containing protein
	bpuSUM 001486	PF05714	Borrelia Bbcrasp-1 domain containing protein
	bpuSUM 001487	PF05714	Borrelia Bbcrasp-1 domain containing protein
	bpuSUM 001488	PF05714	Borrelia Bbcrasp-1 domain containing protein
B. parkeri **SLO**	bpSLO 001051	PF05714	Borrelia Bbcrasp-1 domain containing protein
	bpSLO 001052	PF05714	Borrelia Bbcrasp-1 domain containing protein
	bpSLO 001053	PF05714	Borrelia Bbcrasp-1 domain containing protein
	bpSLO 001054	PF05714	Borrelia Bbcrasp-1 domain containing protein
	bpSLO 001055	not classified by interproscan	
	bpSLO 001056	PF05714	Borrelia Bbcrasp-1 domain containing protein
	bpSLO 001057	PF05714	Borrelia Bbcrasp-1 domain containing protein
	bpSLO 001058	PF05714	Borrelia Bbcrasp-1 domain containing protein
	bpSLO 001059	PF05714	Borrelia Bbcrasp-1 domain containing protein
	bpSLO 001060	PF05714	Borrelia Bbcrasp-1 domain containing protein
	bpSLO 001061	PF05714	Borrelia Bbcrasp-1 domain containing protein
	bpSLO 001062	PF05714	Borrelia Bbcrasp-1 domain containing protein
	bpSLO 001063	PF05714	Borrelia Bbcrasp-1 domain containing protein
	bpSLO 001064	PF05714	Borrelia Bbcrasp-1 domain containing protein
	bpSLO 001065	PF05714	Borrelia Bbcrasp-1 domain containing protein
B. venezuelensis **RMA01**	bvRMA01 000926	PF05714	Borrelia Bbcrasp-1 domain containing protein
	bvRMA01 000927	PF05714	Borrelia Bbcrasp-1 domain containing protein
	bvRMA01 000928	PF05714	Borrelia Bbcrasp-1 domain containing protein
	bvRMA01 000929	PF05714	Borrelia Bbcrasp-1 domain containing protein
	bvRMA01 000930	not classified by interproscan	
	bvRMA01 000931	PF05714	Borrelia Bbcrasp-1 domain containing protein
	bvRMA01 000932	PF05714	Borrelia Bbcrasp-1 domain containing protein
	bvRMA01 000933	PF05714	Borrelia Bbcrasp-1 domain containing protein
	bvRMA01 000934	PF05714	Borrelia Bbcrasp-1 domain containing protein
	bvRMA01 000935	PF05714	Borrelia Bbcrasp-1 domain containing protein
	bvRMA01 000936	PF05714	Borrelia Bbcrasp-1 domain containing protein
	bvRMA01 000937	PF05714	Borrelia Bbcrasp-1 domain containing protein
B. turicatae **91E135**	bt91E135 001096	PF05714	Borrelia Bbcrasp-1 domain containing protein
	bt91E135 001097	PF05714	Borrelia Bbcrasp-1 domain containing protein
	bt91E135 001098	PF05714	Borrelia Bbcrasp-1 domain containing protein
	bt91E135 001099	PF05714	Borrelia Bbcrasp-1 domain containing protein
	bt91E135 001100	not classified by interproscan	
	bt91E135 001101	PF05714	Borrelia Bbcrasp-1 domain containing protein
	bt91E135 001102	PF05714	Borrelia Bbcrasp-1 domain containing protein
	bt91E135 001103	PF05714	Borrelia Bbcrasp-1 domain containing protein
	bt91E135 001104	PF05714	Borrelia Bbcrasp-1 domain containing protein
	bt91E135 001105	PF05714	Borrelia Bbcrasp-1 domain containing protein
	bt91E135 001106	PF05714	Borrelia Bbcrasp-1 domain containing protein
	bt91E135 001107	PF05714	Borrelia Bbcrasp-1 domain containing protein
	bt91E135 001108	PF05714	Borrelia Bbcrasp-1 domain containing protein
	bt91E135 001109	PF05714	Borrelia Bbcrasp-1 domain containing protein
	bt91E135 001110	PF05714	Borrelia Bbcrasp-1 domain containing protein
B. turicatae **BTE5EL**	btBTE5EL 001107	PF05714	Borrelia Bbcrasp-1 domain containing protein
	btBTE5EL 001108	PF05714	Borrelia Bbcrasp-1 domain containing protein
	btBTE5EL 001109	PF05714	Borrelia Bbcrasp-1 domain containing protein
	btBTE5EL 001110	PF05714	Borrelia Bbcrasp-1 domain containing protein
	btBTE5EL 001111	not classified by interproscan	
	btBTE5EL 001112	PF05714	Borrelia Bbcrasp-1 domain containing protein
	btBTE5EL 001113	PF05714	Borrelia Bbcrasp-1 domain containing protein
	btBTE5EL 001114	PF05714	Borrelia Bbcrasp-1 domain containing protein
	btBTE5EL 001115	PF05714	Borrelia Bbcrasp-1 domain containing protein
	btBTE5EL 001116	PF05714	Borrelia Bbcrasp-1 domain containing protein
	btBTE5EL 001117	PF05714	Borrelia Bbcrasp-1 domain containing protein
	btBTE5EL 001118	PF05714	Borrelia Bbcrasp-1 domain containing protein
	btBTE5EL 001119	PF05714	Borrelia Bbcrasp-1 domain containing protein
	btBTE5EL 001120	PF05714	Borrelia Bbcrasp-1 domain containing protein
	btBTE5EL 001121	PF05714	Borrelia Bbcrasp-1 domain containing protein

Block B	locus	PFAM/SSF Designation by InterProScan	Description
B. anserina BA2	baBA2_000925	not classified by interproscan	
	baBA2_000926	not classified by interproscan	
	baBA2_000927	not classified by interproscan	
	baBA2_000928	not classified by interproscan	
	baBA2_000929	not classified by interproscan	
	baBA2_000930	not classified by interproscan	
	baBA2_000931	not classified by interproscan	
	baBA2_000932	not classified by interproscan	
	baBA2_000933	not classified by interproscan	
	baBA2_000934	not classified by interproscan	
	baBA2_000935	not classified by interproscan	
	baBA2_000936	not classified by interproscan	
	baBA2_000937	not classified by interproscan	
B. hermsii DAH	bhDAH_001186	PF12815	Spt5 C-terminal nonapeptide repeat binding Spt4
	bhDAH_001187	not classified by interproscan	
	bhDAH_001188	cd03796	GT4_PIG-A-like
	bhDAH_001189	not classified by interproscan	
	bhDAH_001190	not classified by interproscan	
	bhDAH_001191	not classified by interproscan	
	bhDAH_001192	not classified by interproscan	
	bhDAH_001193	not classified by interproscan	
	bhDAH_001194	not classified by interproscan	
	bhDAH_001195	not classified by interproscan	
	bhDAH_001196	not classified by interproscan	
	bhDAH_001197	not classified by interproscan	
	bhDAH_001198	not classified by interproscan	
	bhDAH_001199	not classified by interproscan	
	bhDAH_001200	not classified by interproscan	
	bhDAH_001201	not classified by interproscan	
	bhDAH_001202	not classified by interproscan	
	bhDAH_001203	not classified by interproscan	
	bhDAH_001204	not classified by interproscan	
	bhDAH_001205	not classified by interproscan	
	bhDAH_001206	PIN_VapC-like	cd18707
	bhDAH_001207	not classified by interproscan	
	bhDAH_001208	not classified by interproscan	
	bhDAH_001209	not classified by interproscan	
	bhDAH_001210	not classified by interproscan	
	bhDAH_001211	not classified by interproscan	
	bhDAH_001212	not classified by interproscan	
	bhDAH_001213	not classified by interproscan	
	bhDAH_001214	not classified by interproscan	
	bhDAH_001215	not classified by interproscan	
B. hermsii YOR	bhYOR_001110	not classified by interproscan	
	bhYOR_001111	not classified by interproscan	
	bhYOR_001112	not classified by interproscan	
	bhYOR_001113	cd03796	GT4_PIG-A-like
	bhYOR_001114	not classified by interproscan	
	bhYOR_001115	not classified by interproscan	
	bhYOR_001116	not classified by interproscan	
	bhYOR_001117	not classified by interproscan	
	bhYOR_001118	not classified by interproscan	
	bhYOR_001119	not classified by interproscan	
	bhYOR_001120	not classified by interproscan	
	bhYOR_001121	not classified by interproscan	
	bhYOR_001122	not classified by interproscan	
	bhYOR_001123	not classified by interproscan	
	bhYOR_001124	not classified by interproscan	
	bhYOR_001125	not classified by interproscan	
	bhYOR_001126	not classified by interproscan	
	bhYOR_001127	not classified by interproscan	
	bhYOR_001128	not classified by interproscan	
	bhYOR_001129	not classified by interproscan	
	bhYOR_001130	not classified by interproscan	
	bhYOR_001131	not classified by interproscan	
	bhYOR_001132	not classified by interproscan	
	bhYOR_001133	not classified by interproscan	
	bhYOR_001134	not classified by interproscan	
	bhYOR_001135	not classified by interproscan	
	bhYOR_001136	not classified by interproscan	
	bhYOR_001137	not classified by interproscan	
	bhYOR_001138	not classified by interproscan	
	bhYOR_001139	not classified by interproscan	
B. coriaceae Co53	bcCo53_001141	not classified by interproscan	
	bcCo53_001142	not classified by interproscan	
	bcCo53_001143	not classified by interproscan	
	bcCo53_001144	not classified by interproscan	
	bcCo53_001145	not classified by interproscan	
	bcCo53_001146	not classified by interproscan	
	bcCo53_001147	not classified by interproscan	
	bcCo53_001148	not classified by interproscan	
	bcCo53_001149	not classified by interproscan	
	bcCo53_001150	not classified by interproscan	
	bcCo53_001151	not classified by interproscan	
	bcCo53_001152	not classified by interproscan	
	bcCo53_001153	not classified by interproscan	
	bcCo53_001154	not classified by interproscan	
	bcCo53_001155	not classified by interproscan	
	bcCo53_001156	not classified by interproscan	
	bcCo53_001157	not classified by interproscan	
	bcCo53_001158	not classified by interproscan	
	bcCo53_001159	not classified by interproscan	
	bcCo53_001160	not classified by interproscan	
	bcCo53_001161	not classified by interproscan	
	bcCo53_001162	not classified by interproscan	
	bcCo53_001163	not classified by interproscan	
B. puertoricensis SUM	bpuSUM_001492	not classified by interproscan	
	bpuSUM_001493	not classified by interproscan	
	bpuSUM_001494	PF05714	Borrelia Bbcrasp-1 domain containing protein
	bpuSUM_001495	PF01391	Collagen triple helix repeat (20 copies)
	bpuSUM_001496	PF01391	Collagen triple helix repeat (20 copies)
	bpuSUM_001497	not classified by interproscan	
	bpuSUM_001498	not classified by interproscan	
	bpuSUM_001499	not classified by interproscan	
	bpuSUM_001500	not classified by interproscan	
	bpuSUM_001501	not classified by interproscan	
	bpuSUM_001502	not classified by interproscan	
	bpuSUM_001503	not classified by interproscan	
	bpuSUM_001504	not classified by interproscan	
	bpuSUM_001505	not classified by interproscan	
	bpuSUM_001506	not classified by interproscan	
	bpuSUM_001507	not classified by interproscan	
	bpuSUM_001508	not classified by interproscan	
	bpuSUM_001509	not classified by interproscan	
B. parkeri SLO	bpSLO_001069	not classified by interproscan	
	bpSLO_001070	not classified by interproscan	
	bpSLO_001071	not classified by interproscan	
	bpSLO_001072	PF05714	Borrelia Bbcrasp-1 domain containing protein
	bpSLO_001073	PF01391	Collagen triple helix repeat (20 copies)
	bpSLO_001074	not classified by interproscan	
	bpSLO_001075	not classified by interproscan	
	bpSLO_001076	not classified by interproscan	
	bpSLO_001077	not classified by interproscan	
	bpSLO_001078	not classified by interproscan	
	bpSLO_001079	not classified by interproscan	
	bpSLO_001080	not classified by interproscan	
	bpSLO_001081	not classified by interproscan	
	bpSLO_001082	not classified by interproscan	
	bpSLO_001083	not classified by interproscan	
	bpSLO_001084	PF05714	Borrelia Bbcrasp-1 domain containing protein
	bpSLO_001085	not classified by interproscan	
	bpSLO_001086	not classified by interproscan	
	bpSLO_001087	not classified by interproscan	
	bpSLO_001088	PF05714	Borrelia Bbcrasp-1 domain containing protein
	bpSLO_001089	not classified by interproscan	
	bpSLO_001090	not classified by interproscan	
	bpSLO_001091	not classified by interproscan	
	bpSLO_001092	not classified by interproscan	
B. venezuelensis RMA01	bvRMA01_000941	not classified by interproscan	
	bvRMA01_000942	not classified by interproscan	
	bvRMA01_000943	not classified by interproscan	
	bvRMA01_000944	PF05714	Borrelia Bbcrasp-1 domain containing protein
	bvRMA01_000945	PF01391	Collagen triple helix repeat (20 copies)
	bvRMA01_000946	not classified by interproscan	
	bvRMA01_000947	not classified by interproscan	
	bvRMA01_000948	not classified by interproscan	
	bvRMA01_000949	not classified by interproscan	
	bvRMA01_000950	not classified by interproscan	
	bvRMA01_000951	not classified by interproscan	
	bvRMA01_000952	not classified by interproscan	
	bvRMA01_000953	not classified by interproscan	
	bvRMA01_000954	not classified by interproscan	
	bvRMA01_000955	not classified by interproscan	
	bvRMA01_000956	not classified by interproscan	
	bvRMA01_000957	not classified by interproscan	
	bvRMA01_000958	not classified by interproscan	
	bvRMA01_000959	not classified by interproscan	
	bvRMA01_000960	not classified by interproscan	
	bvRMA01_000961	PF05714	Borrelia Bbcrasp-1 domain containing protein
	bvRMA01_000962	not classified by interproscan	
	bvRMA01_000963	not classified by interproscan	
	bvRMA01_000964	not classified by interproscan	
	bvRMA01_000965	not classified by interproscan	
	bvRMA01_000966	not classified by interproscan	
	bvRMA01_000967	not classified by interproscan	
B. turicatae 91E135	bt91E135_001114	not classified by interproscan	
	bt91E135_001115	not classified by interproscan	
	bt91E135_001116	not classified by interproscan	
	bt91E135_001117	not classified by interproscan	
	bt91E135_001118	PF01391	Collagen triple helix repeat (20 copies)
	bt91E135_001119	not classified by interproscan	
	bt91E135_001120	not classified by interproscan	
	bt91E135_001121	not classified by interproscan	
	bt91E135_001122	not classified by interproscan	
	bt91E135_001123	not classified by interproscan	
	bt91E135_001124	not classified by interproscan	
	bt91E135_001125	not classified by interproscan	
	bt91E135_001126	not classified by interproscan	
	bt91E135_001127	not classified by interproscan	
	bt91E135_001128	not classified by interproscan	
	bt91E135_001129	not classified by interproscan	
	bt91E135_001130	not classified by interproscan	
	bt91E135_001131	not classified by interproscan	
	bt91E135_001132	not classified by interproscan	
	bt91E135_001133	PF05714	Borrelia Bbcrasp-1 domain containing protein
	bt91E135_001134	not classified by interproscan	
	bt91E135_001135	not classified by interproscan	
	bt91E135_001136	not classified by interproscan	
	bt91E135_001137	not classified by interproscan	
B. turicatae BTE5EL	btBTE5EL_001125	not classified by interproscan	
	btBTE5EL_001126	not classified by interproscan	
	btBTE5EL_001127	not classified by interproscan	
	btBTE5EL_001128	not classified by interproscan	
	btBTE5EL_001129	PF01391	Collagen triple helix repeat (20 copies)
	btBTE5EL_001130	not classified by interproscan	
	btBTE5EL_001131	not classified by interproscan	
	btBTE5EL_001132	not classified by interproscan	
	btBTE5EL_001133	not classified by interproscan	
	btBTE5EL_001134	not classified by interproscan	
	btBTE5EL_001135	not classified by interproscan	
	btBTE5EL_001136	not classified by interproscan	
	btBTE5EL_001137	not classified by interproscan	
	btBTE5EL_001138	not classified by interproscan	
	btBTE5EL_001139	not classified by interproscan	
	btBTE5EL_001140	not classified by interproscan	
	btBTE5EL_001141	not classified by interproscan	
	btBTE5EL_001142	not classified by interproscan	
	btBTE5EL_001143	not classified by interproscan	
	btBTE5EL_001144	PF05714	Borrelia Bbcrasp-1 domain containing protein
	btBTE5EL_001145	not classified by interproscan	
	btBTE5EL_001146	not classified by interproscan	
	btBTE5EL_001147	not classified by interproscan	
	btBTE5EL_001148	not classified by interproscan	

Block C	locus	PFAM/SSF Designation by InterProScan	Description
***B. anserina* BA2**	baBA2_000961	not classified by interproscan	
	baBA2_000962	PF17044	Borrelial persistence in ticks protein A
	baBA2_000963	not classified by interproscan	
***B. hermsii* DAH**	bhDAH_001244	not classified by interproscan	
	bhDAH_001245	PF17044	Borrelial persistence in ticks protein A
	bhDAH_001246	PF17044	Borrelial persistence in ticks protein A
	bhDAH_001247	not classified by interproscan	not classified by interproscan
	bhDAH_001248	PF17044	Borrelial persistence in ticks protein A
	bhDAH_001249	PF17044	Borrelial persistence in ticks protein A
	bhDAH_001250	not classified by interproscan	
	bhDAH_001251	PF17044	Borrelial persistence in ticks protein A
	bhDAH_001252	not classified by interproscan	
	bhDAH_001253	PF17044	Borrelial persistence in ticks protein A
	bhDAH_001254	not classified by interproscan	
	bhDAH_001255	PF17044	Borrelial persistence in ticks protein A
	bhDAH_001256	not classified by interproscan	
	bhDAH_001257	PF17044	Borrelial persistence in ticks protein A
	bhDAH_001258	not classified by interproscan	
	bhDAH_001259	PF17044	Borrelial persistence in ticks protein A
	bhDAH_001260	not classified by interproscan	
	bhDAH_001261	not classified by interproscan	
	bhDAH_001262	not classified by interproscan	
	bhDAH_001263	PF17044	Borrelial persistence in ticks protein A
	bhDAH_001264	not classified by interproscan	
	bhDAH_001265	PF02524	KID repeat
	bhDAH_001266	PF17044	Borrelial persistence in ticks protein A
	bhDAH_001267	not classified by interproscan	
	bhDAH_001268	PF17044	Borrelial persistence in ticks protein A
	bhDAH_001269	not classified by interproscan	
***B. hermsii* YOR**	bhYOR_001168	not classified by interproscan	
	bhYOR_001169	PF17044	Borrelial persistence in ticks protein A
	bhYOR_001170	not classified by interproscan	
	bhYOR_001171	PF17044	Borrelial persistence in ticks protein A
	bhYOR_001172	not classified by interproscan	
	bhYOR_001173	PF17044	Borrelial persistence in ticks protein A
	bhYOR_001174	not classified by interproscan	
	bhYOR_001175	PF17044	Borrelial persistence in ticks protein A
	bhYOR_001176	not classified by interproscan	
	bhYOR_001177	PF17044	Borrelial persistence in ticks protein A
	bhYOR_001178	not classified by interproscan	
	bhYOR_001179	PF17044	Borrelial persistence in ticks protein A
	bhYOR_001180	not classified by interproscan	
	bhYOR_001181	not classified by interproscan	
	bhYOR_001182	not classified by interproscan	
	bhYOR_001183	not classified by interproscan	
	bhYOR_001184	PF17044	Borrelial persistence in ticks protein A
	bhYOR_001185	not classified by interproscan	
	bhYOR_001186	SSF58113	Apolipoprotein A-I
	bhYOR_001187	PF17044	Borrelial persistence in ticks protein A
	bhYOR_001188	not classified by interproscan	
	bhYOR_001189	PF17044	Borrelial persistence in ticks protein A
	bhYOR_001190	not classified by interproscan	
***B. coriaceae* Co53**	bcCo53_001189	not classified by interproscan	
	bcCo53_001190	not classified by interproscan	
	bcCo53_001191	PF17044	Borrelial persistence in ticks protein A
	bcCo53_001192	not classified by interproscan	
	bcCo53_001193	PF17044	Borrelial persistence in ticks protein A
	bcCo53_001194	not classified by interproscan	
	bcCo53_001195	PF17044	Borrelial persistence in ticks protein A
	bcCo53_001196	not classified by interproscan	
	bcCo53_001197	PF17044	Borrelial persistence in ticks protein A
	bcCo53_001198	not classified by interproscan	
	bcCo53_001199	PF17044	Borrelial persistence in ticks protein A
	bcCo53_001200	not classified by interproscan	
	bcCo53_001201	PF17044	Borrelial persistence in ticks protein A
	bcCo53_001202	not classified by interproscan	
	bcCo53_001203	PF17044	Borrelial persistence in ticks protein A
	bcCo53_001204	not classified by interproscan	
	bcCo53_001205	PF17044	Borrelial persistence in ticks protein A
	bcCo53_001206	PF17044	Borrelial persistence in ticks protein A
	bcCo53_001207	PF17044	Borrelial persistence in ticks protein A
	bcCo53_001208	not classified by interproscan	
***B. puertoricensis* SUM**	bpuSUM_001533	PF17044	Borrelial persistence in ticks protein A
	bpuSUM_001534	PF17044	Borrelial persistence in ticks protein A
	bpuSUM_001535	not classified by interproscan	
***B. parkeri* SLO**	bpSLO_001117	PF17044	Borrelial persistence in ticks protein A
	bpSLO_001118	not classified by interproscan	
	bpSLO_001119	PF17044	Borrelial persistence in ticks protein A
	bpSLO_001120	PF17044	Borrelial persistence in ticks protein A
	bpSLO_001121	PF17044	Borrelial persistence in ticks protein A
	bpSLO_001122	not classified by interproscan	
	bpSLO_001123	PF17044	Borrelial persistence in ticks protein A
	bpSLO_001124	PF17044	Borrelial persistence in ticks protein A
	bpSLO_001125	not classified by interproscan	
	bpSLO_001126	not classified by interproscan	
	bpSLO_001127	not classified by interproscan	
	bpSLO_001128	PF17044	Borrelial persistence in ticks protein A
	bpSLO_001129	not classified by interproscan	
***B. venezuelensis* RMA01**	bvRMA01_000992	PF17044	Borrelial persistence in ticks protein A
	bvRMA01_000993	PF17044	Borrelial persistence in ticks protein A
	bvRMA01_000994	not classified by interproscan	
	bvRMA01_000995	not classified by interproscan	
***B. turicatae* 91E135**	bt91E135_001162	not classified by interproscan	
	bt91E135_001163	not classified by interproscan	
	bt91E135_001164	PF17044	Borrelial persistence in ticks protein A
	bt91E135_001165	PF17044	Borrelial persistence in ticks protein A
	bt91E135_001166	PF17044	Borrelial persistence in ticks protein A
	bt91E135_001167	not classified by interproscan	
	bt91E135_001168	PF17044	Borrelial persistence in ticks protein A
	bt91E135_001169	not classified by interproscan	
	bt91E135_001170	PF17044	Borrelial persistence in ticks protein A
	bt91E135_001171	not classified by interproscan	
	bt91E135_001172	not classified by interproscan	
	bt91E135_001173	not classified by interproscan	
	bt91E135_001174	not classified by interproscan	
	bt91E135_001175	not classified by interproscan	
	bt91E135_001176	PF17044	Borrelial persistence in ticks protein A
	bt91E135_001177	not classified by interproscan	
***B. turicatae* BTE5EL**	btBTE5EL_001173	not classified by interproscan	
	btBTE5EL_001174	not classified by interproscan	
	btBTE5EL_001175	PF17044	Borrelial persistence in ticks protein A
	btBTE5EL_001176	PF17044	Borrelial persistence in ticks protein A
	btBTE5EL_001177	PF17044	Borrelial persistence in ticks protein A
	btBTE5EL_001178	not classified by interproscan	
	btBTE5EL_001179	PF17044	Borrelial persistence in ticks protein A
	btBTE5EL_001180	not classified by interproscan	
	btBTE5EL_001181	PF17044	Borrelial persistence in ticks protein A
	btBTE5EL_001182	not classified by interproscan	
	btBTE5EL_001183	not classified by interproscan	
	btBTE5EL_001184	not classified by interproscan	
	btBTE5EL_001185	not classified by interproscan	
	btBTE5EL_001186	PF17044	Borrelial persistence in ticks protein A
	btBTE5EL_001187	not classified by interproscan	

Block D	locus	PFAM/SSF Designation by InterProScan	Description
B. anserina BA2	baBA2_000981	not classified by interproscan	
	baBA2_000982	not classified by interproscan	
	baBA2_000983	not classified by interproscan	
	baBA2_000984	not classified by interproscan	
	baBA2_000985	not classified by interproscan	
	baBA2_000986	not classified by interproscan	
	baBA2_000987	not classified by interproscan	
	baBA2_000988	not classified by interproscan	
	baBA2_000989	not classified by interproscan	
	baBA2_000990	SSF69349	Phage fibre proteins
B. hermsii DAH	bhDAH_001291	not classified by interproscan	
	bhDAH_001292	not classified by interproscan	
	bhDAH_001293	not classified by interproscan	
	bhDAH_001294	not classified by interproscan	
	bhDAH_001295	not classified by interproscan	
	bhDAH_001296	not classified by interproscan	
	bhDAH_001297	not classified by interproscan	
	bhDAH_001298	not classified by interproscan	
	bhDAH_001299	not classified by interproscan	
	bhDAH_001300	not classified by interproscan	
	bhDAH_001301	not classified by interproscan	
	bhDAH_001302	not classified by interproscan	
	bhDAH_001303	not classified by interproscan	
	bhDAH_001304	not classified by interproscan	
	bhDAH_001305	not classified by interproscan	
	bhDAH_001306	not classified by interproscan	
	bhDAH_001307	not classified by interproscan	
	bhDAH_001308	not classified by interproscan	
	bhDAH_001309	not classified by interproscan	
B. hermsii YOR	bhYOR_001214	not classified by interproscan	
	bhYOR_001215	not classified by interproscan	
	bhYOR_001216	not classified by interproscan	
	bhYOR_001217	not classified by interproscan	
	bhYOR_001218	not classified by interproscan	
	bhYOR_001219	not classified by interproscan	
	bhYOR_001220	not classified by interproscan	
	bhYOR_001221	not classified by interproscan	
	bhYOR_001222	not classified by interproscan	
	bhYOR_001223	not classified by interproscan	
	bhYOR_001224	not classified by interproscan	
	bhYOR_001225	not classified by interproscan	
	bhYOR_001226	not classified by interproscan	
	bhYOR_001227	not classified by interproscan	
	bhYOR_001228	not classified by interproscan	
	bhYOR_001229	not classified by interproscan	
	bhYOR_001230	not classified by interproscan	
	bhYOR_001231	not classified by interproscan	
	bhYOR_001232	not classified by interproscan	
	bcCo53_001231	not classified by interproscan	
B. coriaceae Co53	bcCo53_001232	not classified by interproscan	
	bcCo53_001233	not classified by interproscan	
	bcCo53_001234	not classified by interproscan	
	bcCo53_001235	not classified by interproscan	
	bcCo53_001236	not classified by interproscan	
	bcCo53_001237	not classified by interproscan	
	bcCo53_001238	not classified by interproscan	
	bcCo53_001239	not classified by interproscan	
	bcCo53_001240	not classified by interproscan	
	bcCo53_001241	not classified by interproscan	
	bcCo53_001242	not classified by interproscan	
	bcCo53_001243	not classified by interproscan	
	bcCo53_001244	not classified by interproscan	
	bcCo53_001245	not classified by interproscan	
	bcCo53_001246	not classified by interproscan	
	bcCo53_001247	not classified by interproscan	
	bcCo53_001248	not classified by interproscan	
	bcCo53_001249	not classified by interproscan	
	bcCo53_001250	not classified by interproscan	
	bcCo53_001251	not classified by interproscan	
	bcCo53_001252	not classified by interproscan	
	bcCo53_001253	not classified by interproscan	
	bcCo53_001254	not classified by interproscan	
	bcCo53_001255	not classified by interproscan	
	bcCo53_001256	not classified by interproscan	
	bcCo53_001257	not classified by interproscan	
	bcCo53_001258	not classified by interproscan	
	bcCo53_001259	not classified by interproscan	
	bcCo53_001260	not classified by interproscan	
	bcCo53_001261	not classified by interproscan	
B. puertoricensis SUM	none		
B. parkeri SLO	bpSLO_001155	not classified by interproscan	
	bpSLO_001156	not classified by interproscan	
	bpSLO_001157	not classified by interproscan	
	bpSLO_001158	not classified by interproscan	
	bpSLO_001159	PF07761	Protein of unknown function (DUF1617)
	bpSLO_001160	not classified by interproscan	
	bpSLO_001161	not classified by interproscan	
	bpSLO_001162	PF01391	Collagen triple helix repeat (20 copies)
	bpSLO_001163	not classified by interproscan	
	bpSLO_001164	not classified by interproscan	
	bpSLO_001165	not classified by interproscan	
	bpSLO_001166	not classified by interproscan	
	bpSLO_001167	not classified by interproscan	
	bpSLO_001168	not classified by interproscan	
	bpSLO_001169	not classified by interproscan	
	bpSLO_001170	not classified by interproscan	
	bpSLO_001171	not classified by interproscan	
	bpSLO_001172	not classified by interproscan	
	bpSLO_001173	SSF51161	Trimeric LpxA-like enzymes
	bpSLO_001174	not classified by interproscan	
	bpSLO_001175	not classified by interproscan	
	bpSLO_001176	not classified by interproscan	
	bpSLO_001177	not classified by interproscan	
	bpSLO_001178	not classified by interproscan	
	bpSLO_001179	not classified by interproscan	
	bpSLO_001180	not classified by interproscan	
	bpSLO_001181	not classified by interproscan	
B. venezuelensis RMA01	bvRMA01_001022	not classified by interproscan	
	bvRMA01_001023	not classified by interproscan	
	bvRMA01_001024	not classified by interproscan	
	bvRMA01_001025	not classified by interproscan	
	bvRMA01_001026	not classified by interproscan	
	bvRMA01_001027	not classified by interproscan	
	bvRMA01_001028	not classified by interproscan	
	bvRMA01_001029	not classified by interproscan	
	bvRMA01_001030	not classified by interproscan	
	bvRMA01_001031	not classified by interproscan	
	bvRMA01_001032	not classified by interproscan	
	bvRMA01_001033	not classified by interproscan	
	bvRMA01_001034	not classified by interproscan	
	bvRMA01_001035	not classified by interproscan	
	bvRMA01_001036	not classified by interproscan	
	bvRMA01_001037	cd00139	PIPKc
	bvRMA01_001038	not classified by interproscan	
	bvRMA01_001039	not classified by interproscan	
	bvRMA01_001040	not classified by interproscan	
B. turicatae 91E135	bt91E135_001201	not classified by interproscan	
	bt91E135_001202	not classified by interproscan	
	bt91E135_001203	not classified by interproscan	
	bt91E135_001204	not classified by interproscan	
	bt91E135_001205	not classified by interproscan	
	bt91E135_001206	PF07761	Protein of unknown function (DUF1617)
	bt91E135_001207	not classified by interproscan	
	bt91E135_001208	not classified by interproscan	
	bt91E135_001209	not classified by interproscan	
	bt91E135_001210	not classified by interproscan	
	bt91E135_001211	not classified by interproscan	
	bt91E135_001212	not classified by interproscan	
	bt91E135_001213	PF07761	Protein of unknown function (DUF1617)
	bt91E135_001214	not classified by interproscan	
	bt91E135_001215	not classified by interproscan	
	bt91E135_001216	not classified by interproscan	
	bt91E135_001217	not classified by interproscan	
	bt91E135_001218	not classified by interproscan	
	bt91E135_001219	not classified by interproscan	
	bt91E135_001220	not classified by interproscan	
	bt91E135_001221	not classified by interproscan	
	bt91E135_001222	not classified by interproscan	
	bt91E135_001223	not classified by interproscan	
	bt91E135_001224	not classified by interproscan	
	bt91E135_001225	not classified by interproscan	
	bt91E135_001226	not classified by interproscan	
	bt91E135_001227	not classified by interproscan	
	bt91E135_001228	not classified by interproscan	
B. turicatae BTE5EL	btBTE5EL_001211	not classified by interproscan	
	btBTE5EL_001212	not classified by interproscan	
	btBTE5EL_001213	not classified by interproscan	
	btBTE5EL_001214	not classified by interproscan	
	btBTE5EL_001215	not classified by interproscan	
	btBTE5EL_001216	PF07761	Protein of unknown function (DUF1617)
	btBTE5EL_001217	not classified by interproscan	
	btBTE5EL_001218	not classified by interproscan	
	btBTE5EL_001219	not classified by interproscan	
	btBTE5EL_001220	not classified by interproscan	
	btBTE5EL_001221	not classified by interproscan	
	btBTE5EL_001222	not classified by interproscan	
	btBTE5EL_001223	PF07761	Protein of unknown function (DUF1617)
	btBTE5EL_001224	not classified by interproscan	
	btBTE5EL_001225	not classified by interproscan	
	btBTE5EL_001226	not classified by interproscan	
	btBTE5EL_001227	not classified by interproscan	
	btBTE5EL_001228	not classified by interproscan	
	btBTE5EL_001229	not classified by interproscan	
	btBTE5EL_001230	not classified by interproscan	
	btBTE5EL_001231	not classified by interproscan	
	btBTE5EL_001232	not classified by interproscan	
	btBTE5EL_001233	not classified by interproscan	
	btBTE5EL_001234	not classified by interproscan	
	btBTE5EL_001235	not classified by interproscan	
	btBTE5EL_001236	not classified by interproscan	
	btBTE5EL_001237	not classified by interproscan	
	btBTE5EL_001238	not classified by interproscan	
	btBTE5EL_001239	not classified by interproscan	
	btBTE5EL_001240	not classified by interproscan	

Block E	locus	PFAM/SSF Designation by InterProScan	Description
B. anserina **BA2**	none		
B. hermsii **DAH**	bhDAH_001311	not classified by interproscan	
B. hermsii **YOR**	none		
B. coriaceae **Co53**	none		
B. puertoricensis **SUM**	none		
B. parkeri **SLO**	none		
B. venezuelensis **RMA01**	none		
B. turicatae **91E135**	none		
B. turicatae **BTE5EL**	none		
