# Supplementary material for: Comparative genomics analysis of three conserved plasmid families in the Western Hemisphere soft tick-borne relapsing fever borreliae provides insight into variation in genome structure and antigenic variation systems
Source: bioRxiv. 2023 Mar 6:2023.03.06.531354. Preprint. [Version 1] doi: 10.1101/2023.03.06.531354 (PMC10028826; doi:10.1101/2023.03.06.531354)
Supplement: Supplement 5 [file media-5.pdf]

This file contains the loci classified as either a vsp or vlp for each of the isolates investigated. Vlp genes are further classified by subfamily.

Vsp	replicon	gene/pseudo								
gene-baBA2_0009	lp27	gene	Pfam	protein_matc	37	205	4.3E-58	+	.	Target=null 37 205;ID=match$1484_37_205;signature_desc=Lipoprotein;Name=PF01441;status=T;Dbxref="InterPro:IPR001800"

Vlp
none

Vsp	replicon	gene/pseudo								
gene-bhDAH_001040 (	cp90	gene	Pfam	protein_matc	38	205	6E-59	+	.	Target=null 38 205;ID=match$1421_38_205;signature_desc=Lipoprotein;Name=PF01441;status=T;Dbxref="InterPro:IPR001800"
gene-bhDAH_001041 (	cp90	pseudo	Pfam	protein_matc	21	61	7.3E-13	+	.	Target=null 21 61;ID=match$1591_21_61;signature_desc=Lipoprotein;Name=PF01441;status=T;Dbxref="InterPro:IPR001800"
gene-bhDAH_001053 (	cp90	pseudo	Pfam	protein_matc	257	398	4.7E-32	+	.	Target=null 257 398;ID=match$493_257_398;signature_desc=Lipoprotein;Name=PF01441;status=T;Dbxref="InterPro:IPR001800"
gene-bhDAH_001055 (	cp90	gene	Pfam	protein_matc	2	65	9E-10	+	.	Target=null 2 65;ID=match$1289_2_65;signature_desc=Lipoprotein;Name=PF01441;status=T;Dbxref="InterPro:IPR001800"
gene-bhDAH_001058 (	cp90	gene	Pfam	protein_matc	47	209	5.8E-38	+	.	Target=null 47 209;ID=match$2796_47_209;signature_desc=Lipoprotein;Name=PF01441;status=T;Dbxref="InterPro:IPR001800"
gene-bhDAH_001089 (	cp90	pseudo	Pfam	protein_matc	40	114	6.7E-27	+	.	Target=null 40 114;ID=match$103_40_114;signature_desc=Lipoprotein;Name=PF01441;status=T;Dbxref="InterPro:IPR001800"
gene-bhDAH_001089 (	cp90	pseudo	Pfam	protein_matc	2	35	1.8E-08	+	.	Target=null 2 35;ID=match$934_2_35;signature_desc=Lipoprotein;Name=PF01441;status=T;Dbxref="InterPro:IPR001800"
gene-bhDAH_001093 (	cp90	pseudo	Pfam	protein_matc	2	167	2.3E-52	+	.	Target=null 2 167;ID=match$1056_2_167;signature_desc=Lipoprotein;Name=PF01441;status=T;Dbxref="InterPro:IPR001800"
gene-bhDAH_001101 (	cp90	gene	Pfam	protein_matc	1	169	2.7E-67	+	.	Target=null 1 169;ID=match$2690_1_169;signature_desc=Lipoprotein;Name=PF01441;status=T;Dbxref="InterPro:IPR001800"
gene-bhDAH_001121 (	cp90	pseudo	Pfam	protein_matc	38	165	8.3E-36	+	.	Target=null 38 165;ID=match$528_38_165;signature_desc=Lipoprotein;Name=PF01441;status=T;Dbxref="InterPro:IPR001800"
gene-bhDAH_001121 (	cp90	pseudo	Pfam	protein_matc	3	50	1.1E-10	+	.	Target=null 3 50;ID=match$2604_3_50;signature_desc=Lipoprotein;Name=PF01441;status=T;Dbxref="InterPro:IPR001800"
gene-bhDAH_001328 (	lp26	gene	Pfam	protein_matc	29	197	2.1E-66	+	.	Target=null 29 197;ID=match$2243_29_197;signature_desc=Lipoprotein;Name=PF01441;status=T;Dbxref="InterPro:IPR001800"
gene-bhDAH_001331 (	lp26	pseudo	Pfam	protein_matc	38	79	4E-16	+	.	Target=null 38 79;ID=match$1921_38_79;signature_desc=Lipoprotein;Name=PF01441;status=T;Dbxref="InterPro:IPR001800"
gene-bhDAH_001331 (	lp26	pseudo	Pfam	protein_matc	2	126	8.2E-41	+	.	Target=null 2 126;ID=match$1455_2_126;signature_desc=Lipoprotein;Name=PF01441;status=T;Dbxref="InterPro:IPR001800"
gene-bhDAH_001334 (	lp26	pseudo	Pfam	protein_matc	29	70	2.2E-17	+	.	Target=null 29 70;ID=match$1948_29_70;signature_desc=Lipoprotein;Name=PF01441;status=T;Dbxref="InterPro:IPR001800"
gene-bhDAH_001334 (	lp26	pseudo	Pfam	protein_matc	2	127	1.1E-40	+	.	Target=null 2 127;ID=match$1050_2_127;signature_desc=Lipoprotein;Name=PF01441;status=T;Dbxref="InterPro:IPR001800"
gene-bhDAH_001337 (	lp26	gene	Pfam	protein_matc	38	207	2.7E-66	+	.	Target=null 38 207;ID=match$204_38_207;signature_desc=Lipoprotein;Name=PF01441;status=T;Dbxref="InterPro:IPR001800"
gene-bhDAH_001354 (	lp27	gene	Pfam	protein_matc	29	200	8.7E-45	+	.	Target=null 29 200;ID=match$1681_29_200;signature_desc=Lipoprotein;Name=PF01441;status=T;Dbxref="InterPro:IPR001800"
gene-bhDAH_001405 (	lp31	gene	Pfam	protein_matc	22	192	1.3E-51	+	.	Target=null 22 192;ID=match$956_22_192;signature_desc=Lipoprotein;Name=PF01441;status=T;Dbxref="InterPro:IPR001800"
gene-bhDAH_001407 (	lp31	gene	Pfam	protein_matc	1	116	8.1E-35	+	.	Target=null 1 116;ID=match$929_1_116;signature_desc=Lipoprotein;Name=PF01441;status=T;Dbxref="InterPro:IPR001800"
gene-bhDAH_001408 (	lp31	gene	Pfam	protein_matc	38	207	1.9E-58	+	.	Target=null 38 207;ID=match$19_38_207;signature_desc=Lipoprotein;Name=PF01441;status=T;Dbxref="InterPro:IPR001800"
gene-bhDAH_001419 (	lp31	gene	Pfam	protein_matc	38	210	2.6E-58	+	.	Target=null 38 210;ID=match$2482_38_210;signature_desc=Lipoprotein;Name=PF01441;status=T;Dbxref="InterPro:IPR001800"
gene-bhDAH_001420 (	lp31	gene	Pfam	protein_matc	29	197	1.1E-62	+	.	Target=null 29 197;ID=match$2024_29_197;signature_desc=Lipoprotein;Name=PF01441;status=T;Dbxref="InterPro:IPR001800"
gene-bhDAH_001422 (	lp31	pseudo	Pfam	protein_matc	43	214	1.2E-53	+	.	Target=null 43 214;ID=match$511_43_214;signature_desc=Lipoprotein;Name=PF01441;status=T;Dbxref="InterPro:IPR001800"
gene-bhDAH_001423 (	lp31	pseudo	Pfam	protein_matc	7	172	1E-40	+	.	Target=null 7 172;ID=match$1227_7_172;signature_desc=Lipoprotein;Name=PF01441;status=T;Dbxref="InterPro:IPR001800"
gene-bhDAH_001447 (	lp59	gene	Pfam	protein_matc	29	199	2.1E-60	+	.	Target=null 29 199;ID=match$1337_29_199;signature_desc=Lipoprotein;Name=PF01441;status=T;Dbxref="InterPro:IPR001800"
gene-bhDAH_001449 (	lp59	gene	Pfam	protein_matc	25	188	4.6E-49	+	.	Target=null 25 188;ID=match$562_25_188;signature_desc=Lipoprotein;Name=PF01441;status=T;Dbxref="InterPro:IPR001800"
gene-bhDAH_001450 (	lp59	gene	Pfam	protein_matc	38	207	1.6E-52	+	.	Target=null 38 207;ID=match$92_38_207;signature_desc=Lipoprotein;Name=PF01441;status=T;Dbxref="InterPro:IPR001800"
gene-bhDAH_001454 (	lp59	gene	Pfam	protein_matc	38	209	1.3E-44	+	.	Target=null 38 209;ID=match$1000_38_209;signature_desc=Lipoprotein;Name=PF01441;status=T;Dbxref="InterPro:IPR001800"
gene-bhDAH_001455 (	lp59	gene	Pfam	protein_matc	38	206	6.3E-60	+	.	Target=null 38 206;ID=match$1364_38_206;signature_desc=Lipoprotein;Name=PF01441;status=T;Dbxref="InterPro:IPR001800"
gene-bhDAH_001456 (	lp59	gene	Pfam	protein_matc	38	206	3.2E-66	+	.	Target=null 38 206;ID=match$2899_38_206;signature_desc=Lipoprotein;Name=PF01441;status=T;Dbxref="InterPro:IPR001800"
gene-bhDAH_001490 (	lp59	gene	Pfam	protein_matc	38	201	6E-53	+	.	Target=null 38 201;ID=match$1958_38_201;signature_desc=Lipoprotein;Name=PF01441;status=T;Dbxref="InterPro:IPR001800"

Vlp			Subfamily								
gene-bhDAH_001038 (	cp90	gene	gamma	Pfam	protein_matc	2	69	3.2E-10	+	.	Target=null 2 69;ID=match$1517_2_69;signature_desc=Borrelia lipoprotein;Name=PF00921;status=T;Dbxref="InterPro:IPR000680"
gene-bhDAH_001039 (	cp90	gene	gamma	Pfam	protein_matc	4	97	1.5E-14	+	.	Target=null 4 97;ID=match$2620_4_97;signature_desc=Borrelia lipoprotein;Name=PF00921;status=T;Dbxref="InterPro:IPR000680"
gene-bhDAH_001048 (	cp90	gene	gamma	Pfam	protein_matc	30	311	2E-83	+	.	Target=null 30 311;ID=match$2791_30_311;signature_desc=Borrelia lipoprotein;Name=PF00921;status=T;Dbxref="InterPro:IPR000680"
gene-bhDAH_001049 (	cp90	gene	delta	Pfam	protein_matc	43	322	1.6E-84	+	.	Target=null 43 322;ID=match$2080_43_322;signature_desc=Borrelia lipoprotein;Name=PF00921;status=T;Dbxref="InterPro:IPR000680"
gene-bhDAH_001050 (	cp90	gene	gamma	Pfam	protein_matc	43	321	6E-93	+	.	Target=null 43 321;ID=match$2084_43_321;signature_desc=Borrelia lipoprotein;Name=PF00921;status=T;Dbxref="InterPro:IPR000680"
gene-bhDAH_001051 (	cp90	pseudo	beta	Pfam	protein_matc	10	265	4E-42	+	.	Target=null 10 265;ID=match$1812_10_265;signature_desc=Borrelia lipoprotein;Name=PF00921;status=T;Dbxref="InterPro:IPR000680"
gene-bhDAH_001052 (	cp90	pseudo	gamma	Pfam	protein_matc	7	53	2.8E-15	+	.	Target=null 7 53;ID=match$363_7_53;signature_desc=Borrelia lipoprotein;Name=PF00921;status=T;Dbxref="InterPro:IPR000680"
gene-bhDAH_001052 (	cp90	pseudo		Pfam	protein_matc	1	240	6.9E-60	+	.	Target=null 1 240;ID=match$265_1_240;signature_desc=Borrelia lipoprotein;Name=PF00921;status=T;Dbxref="InterPro:IPR000680"
gene-bhDAH_001053 (	cp90	pseudo	delta	Pfam	protein_matc	43	88	4.2E-16	+	.	Target=null 43 88;ID=match$438_43_88;signature_desc=Borrelia lipoprotein;Name=PF00921;status=T;Dbxref="InterPro:IPR000680"
gene-bhDAH_001053 (	cp90	pseudo		Pfam	protein_matc	2	249	4.5E-56	+	.	Target=null 2 249;ID=match$492_2_249;signature_desc=Borrelia lipoprotein;Name=PF00921;status=T;Dbxref="InterPro:IPR000680"
gene-bhDAH_001054 (	cp90	gene	gamma	Pfam	protein_matc	11	271	4.8E-75	+	.	Target=null 11 271;ID=match$1767_11_271;signature_desc=Borrelia lipoprotein;Name=PF00921;status=T;Dbxref="InterPro:IPR000680"
gene-bhDAH_001055 (	cp90	gene	delta	Pfam	protein_matc	25	271	7E-56	+	.	Target=null 25 271;ID=match$1291_25_271;signature_desc=Borrelia lipoprotein;Name=PF00921;status=T;Dbxref="InterPro:IPR000680"
gene-bhDAH_001056 (	cp90	gene	gamma	Pfam	protein_matc	34	312	5.4E-93	+	.	Target=null 34 312;ID=match$2655_34_312;signature_desc=Borrelia lipoprotein;Name=PF00921;status=T;Dbxref="InterPro:IPR000680"
gene-bhDAH_001057 (	cp90	pseudo	gamma	Pfam	protein_matc	25	317	6.8E-94	+	.	Target=null 25 317;ID=match$2987_25_317;signature_desc=Borrelia lipoprotein;Name=PF00921;status=T;Dbxref="InterPro:IPR000680"
gene-bhDAH_001059 (	cp90	pseudo	gamma	Pfam	protein_matc	25	317	3.6E-91	+	.	Target=null 25 317;ID=match$2272_25_317;signature_desc=Borrelia lipoprotein;Name=PF00921;status=T;Dbxref="InterPro:IPR000680"
gene-bhDAH_001060 (	cp90	gene	beta	Pfam	protein_matc	47	336	2.3E-51	+	.	Target=null 47 336;ID=match$764_47_336;signature_desc=Borrelia lipoprotein;Name=PF00921;status=T;Dbxref="InterPro:IPR000680"
gene-bhDAH_001069 (	cp90	gene	delta	Pfam	protein_matc	43	335	1.6E-86	+	.	Target=null 43 335;ID=match$197_43_335;signature_desc=Borrelia lipoprotein;Name=PF00921;status=T;Dbxref="InterPro:IPR000680"
gene-bhDAH_001070 (	cp90	pseudo	gamma	Pfam	protein_matc	13	45	1.1E-11	+	.	Target=null 13 45;ID=match$378_13_45;signature_desc=Borrelia lipoprotein;Name=PF00921;status=T;Dbxref="InterPro:IPR000680"
gene-bhDAH_001092 (	cp90	gene	alpha	Pfam	protein_matc	59	348	1.4E-67	+	.	Target=null 59 348;ID=match$1719_59_348;signature_desc=Borrelia lipoprotein;Name=PF00921;status=T;Dbxref="InterPro:IPR000680"
gene-bhDAH_001094 (	cp90	gene	beta	Pfam	protein_matc	55	345	1.9E-42	+	.	Target=null 55 345;ID=match$408_55_345;signature_desc=Borrelia lipoprotein;Name=PF00921;status=T;Dbxref="InterPro:IPR000680"
gene-bhDAH_001095 (	cp90	gene	delta	Pfam	protein_matc	43	326	5.6E-83	+	.	Target=null 43 326;ID=match$1735_43_326;signature_desc=Borrelia lipoprotein;Name=PF00921;status=T;Dbxref="InterPro:IPR000680"
gene-bhDAH_001096 (	cp90	gene	alpha	Pfam	protein_matc	27	327	7.7E-64	+	.	Target=null 27 327;ID=match$1180_27_327;signature_desc=Borrelia lipoprotein;Name=PF00921;status=T;Dbxref="InterPro:IPR000680"
gene-bhDAH_001097 (	cp90	gene	beta	Pfam	protein_matc	20	310	6.2E-49	+	.	Target=null 20 310;ID=match$1029_20_310;signature_desc=Borrelia lipoprotein;Name=PF00921;status=T;Dbxref="InterPro:IPR000680"
gene-bhDAH_001099 (	cp90	gene	gamma	Pfam	protein_matc	43	207	5.3E-44	+	.	Target=null 43 207;ID=match$2900_43_207;signature_desc=Borrelia lipoprotein;Name=PF00921;status=T;Dbxref="InterPro:IPR000680"
gene-bhDAH_001099 (	cp90	gene		Pfam	protein_matc	200	275	4.1E-28	+	.	Target=null 200 275;ID=match$2900_200_275;signature_desc=Borrelia lipoprotein;Name=PF00921;status=T;Dbxref="InterPro:IPR000680"
gene-bhDAH_001100 (	cp90	gene	delta	Pfam	protein_matc	20	304	4.8E-87	+	.	Target=null 20 304;ID=match$80_20_304;signature_desc=Borrelia lipoprotein;Name=PF00921;status=T;Dbxref="InterPro:IPR000680"
gene-bhDAH_001102 (	cp90	gene	gamma	Pfam	protein_matc	176	251	3.6E-27	+	.	Target=null 176 251;ID=match$230_176_251;signature_desc=Borrelia lipoprotein;Name=PF00921;status=T;Dbxref="InterPro:IPR000680"
gene-bhDAH_001102 (	cp90	gene		Pfam	protein_matc	19	183	4.1E-44	+	.	Target=null 19 183;ID=match$230_19_183;signature_desc=Borrelia lipoprotein;Name=PF00921;status=T;Dbxref="InterPro:IPR000680"
gene-bhDAH_001119 (	cp90	pseudo	delta	Pfam	protein_matc	34	127	3.2E-18	+	.	Target=null 34 127;ID=match$2703_34_127;signature_desc=Borrelia lipoprotein;Name=PF00921;status=T;Dbxref="InterPro:IPR000680"
gene-bhDAH_001120 (	cp90	gene	gamma	Pfam	protein_matc	19	306	4E-91	+	.	Target=null 19 306;ID=match$706_19_306;signature_desc=Borrelia lipoprotein;Name=PF00921;status=T;Dbxref="InterPro:IPR000680"
gene-bhDAH_001122 (	cp90	gene	beta	Pfam	protein_matc	20	312	3.6E-49	+	.	Target=null 20 312;ID=match$2340_20_312;signature_desc=Borrelia lipoprotein;Name=PF00921;status=T;Dbxref="InterPro:IPR000680"
gene-bhDAH_001123 (	cp90	gene	alpha	Pfam	protein_matc	59	349	2.4E-70	+	.	Target=null 59 349;ID=match$2156_59_349;signature_desc=Borrelia lipoprotein;Name=PF00921;status=T;Dbxref="InterPro:IPR000680"
gene-bhDAH_001124 (	cp90	pseudo	delta	Pfam	protein_matc	1	168	9.1E-33	+	.	Target=null 1 168;ID=match$2128_1_168;signature_desc=Borrelia lipoprotein;Name=PF00921;status=T;Dbxref="InterPro:IPR000680"
gene-bhDAH_001124 (	cp90	pseudo		Pfam	protein_matc	8	39	3.1E-10	+	.	Target=null 8 39;ID=match$1019_8_39;signature_desc=Borrelia lipoprotein;Name=PF00921;status=T;Dbxref="InterPro:IPR000680"
gene-bhDAH_001124 (	cp90	pseudo		Pfam	protein_matc	24	58	1.2E-08	+	.	Target=null 24 58;ID=match$1994_24_58;signature_desc=Borrelia lipoprotein;Name=PF00921;status=T;Dbxref="InterPro:IPR000680"
gene-bhDAH_001125 (	cp90	gene	delta	Pfam	protein_matc	53	75	0.000022	+	.	Target=null 53 75;ID=match$2545_53_75;signature_desc=Borrelia lipoprotein;Name=PF00921;status=T;Dbxref="InterPro:IPR000680"
gene-bhDAH_001128 (	cp90	pseudo	gamma	Pfam	protein_matc	13	45	1.1E-11	+	.	Target=null 13 45;ID=match$376_13_45;signature_desc=Borrelia lipoprotein;Name=PF00921;status=T;Dbxref="InterPro:IPR000680"
gene-bhDAH_001129 (	cp90	gene	delta	Pfam	protein_matc	43	326	2.9E-84	+	.	Target=null 43 326;ID=match$614_43_326;signature_desc=Borrelia lipoprotein;Name=PF00921;status=T;Dbxref="InterPro:IPR000680"
gene-bhDAH_001130 (	cp90	gene	gamma	Pfam	protein_matc	5	38	5.7E-08	+	.	Target=null 5 38;ID=match$2909_5_38;signature_desc=Borrelia lipoprotein;Name=PF00921;status=T;Dbxref="InterPro:IPR000680"
gene-bhDAH_001326 (	lp26	gene	gamma	Pfam	protein_matc	19	296	8.1E-80	+	.	Target=null 19 296;ID=match$1350_19_296;signature_desc=Borrelia lipoprotein;Name=PF00921;status=T;Dbxref="InterPro:IPR000680"
gene-bhDAH_001327 (	lp26	gene	delta	Pfam	protein_matc	43	332	6.5E-83	+	.	Target=null 43 332;ID=match$1139_43_332;signature_desc=Borrelia lipoprotein;Name=PF00921;status=T;Dbxref="InterPro:IPR000680"
gene-bhDAH_001329 (	lp26	gene	gamma	Pfam	protein_matc	29	313	4.1E-90	+	.	Target=null 29 313;ID=match$2163_29_313;signature_desc=Borrelia lipoprotein;Name=PF00921;status=T;Dbxref="InterPro:IPR000680"
gene-bhDAH_001330 (	lp26	pseudo	gamma	Pfam	protein_matc	2	118	7.2E-28	+	.	Target=null 2 118;ID=match$748_2_118;signature_desc=Borrelia lipoprotein;Name=PF00921;status=T;Dbxref="InterPro:IPR000680"
gene-bhDAH_001332 (	lp26	gene	gamma	Pfam	protein_matc	9	235	4.2E-59	+	.	Target=null 9 235;ID=match$2061_9_235;signature_desc=Borrelia lipoprotein;Name=PF00921;status=T;Dbxref="InterPro:IPR000680"
gene-bhDAH_001333 (	lp26	pseudo	delta	Pfam	protein_matc	1	88	3.9E-15	+	.	Target=null 1 88;ID=match$1688_1_88;signature_desc=Borrelia lipoprotein;Name=PF00921;status=T;Dbxref="InterPro:IPR000680"
gene-bhDAH_001333 (	lp26	pseudo		Pfam	protein_matc	4	84	4.2E-14	+	.	Target=null 4 84;ID=match$152_4_84;signature_desc=Borrelia lipoprotein;Name=PF00921;status=T;Dbxref="InterPro:IPR000680"
gene-bhDAH_001333 (	lp26	pseudo		Pfam	protein_matc	3	81	4.6E-28	+	.	Target=null 3 81;ID=match$2106_3_81;signature_desc=Borrelia lipoprotein;Name=PF00921;status=T;Dbxref="InterPro:IPR000680"
gene-bhDAH_001335 (	lp26	gene	gamma	Pfam	protein_matc	29	304	1.1E-81	+	.	Target=null 29 304;ID=match$2816_29_304;signature_desc=Borrelia lipoprotein;Name=PF00921;status=T;Dbxref="InterPro:IPR000680"
gene-bhDAH_001336 (	lp26	pseudo	gamma	Pfam	protein_matc	2	66	1.4E-09	+	.	Target=null 2 66;ID=match$1839_2_66;signature_desc=Borrelia lipoprotein;Name=PF00921;status=T;Dbxref="InterPro:IPR000680"
gene-bhDAH_001346 (	lp27	gene	delta	Pfam	protein_matc	34	314	7.4E-87	+	.	Target=null 34 314;ID=match$2881_34_314;signature_desc=Borrelia lipoprotein;Name=PF00921;status=T;Dbxref="InterPro:IPR000680"
gene-bhDAH_001347 (	lp27	pseudo	beta	Pfam	protein_matc	20	74	2.7E-08	+	.	Target=null 20 74;ID=match$981_20_74;signature_desc=Borrelia lipoprotein;Name=PF00921;status=T;Dbxref="InterPro:IPR000680"
gene-bhDAH_001347 (	lp27	pseudo		Pfam	protein_matc	7	221	1.1E-29	+	.	Target=null 7 221;ID=match$573_7_221;signature_desc=Borrelia lipoprotein;Name=PF00921;status=T;Dbxref="InterPro:IPR000680"
gene-bhDAH_001348 (	lp27	pseudo	gamma	Pfam	protein_matc	4	148	1.1E-42	+	.	Target=null 4 148;ID=match$1068_4_148;signature_desc=Borrelia lipoprotein;Name=PF00921;status=T;Dbxref="InterPro:IPR000680"
gene-bhDAH_001355 (	lp27	pseudo	delta	Pfam	protein_matc	9	196	8.2E-42	+	.	Target=null 9 196;ID=match$570_9_196;signature_desc=Borrelia lipoprotein;Name=PF00921;status=T;Dbxref="InterPro:IPR000680"
gene-bhDAH_001355 (	lp27	pseudo		Pfam	protein_matc	33	108	1.6E-26	+	.	Target=null 33 108;ID=match$630_33_108;signature_desc=Borrelia lipoprotein;Name=PF00921;status=T;Dbxref="InterPro:IPR000680"
gene-bhDAH_001356 (	lp27	pseudo	alpha	Pfam	protein_matc	7	74	3.3E-15	+	.	Target=null 7 74;ID=match$963_7_74;signature_desc=Borrelia lipoprotein;Name=PF00921;status=T;Dbxref="InterPro:IPR000680"
gene-bhDAH_001356 (	lp27	pseudo		Pfam	protein_matc	12	224	1.4E-52	+	.	Target=null 12 224;ID=match$2821_12_224;signature_desc=Borrelia lipoprotein;Name=PF00921;status=T;Dbxref="InterPro:IPR000680"
gene-bhDAH_001357 (	lp27	pseudo	beta	Pfam	protein_matc	20	77	4.4E-10	+	.	Target=null 20 77;ID=match$542_20_77;signature_desc=Borrelia lipoprotein;Name=PF00921;status=T;Dbxref="InterPro:IPR000680"
gene-bhDAH_001357 (	lp27	pseudo		Pfam	protein_matc	7	241	4.2E-34	+	.	Target=null 7 241;ID=match$268_7_241;signature_desc=Borrelia lipoprotein;Name=PF00921;status=T;Dbxref="InterPro:IPR000680"
gene-bhDAH_001358 (	lp27	gene	alpha	Pfam	protein_matc	60	351	1.9E-71	+	.	Target=null 60 351;ID=match$2736_60_351;signature_desc=Borrelia lipoprotein;Name=PF00921;status=T;Dbxref="InterPro:IPR000680"
gene-bhDAH_001359 (	lp27	gene	beta	Pfam	protein_matc	20	308	2.1E-42	+	.	Target=null 20 308;ID=match$1105_20_308;signature_desc=Borrelia lipoprotein;Name=PF00921;status=T;Dbxref="InterPro:IPR000680"
gene-bhDAH_001360 (	lp27	pseudo	delta	Pfam	protein_matc	14	290	8.4E-85	+	.	Target=null 14 290;ID=match$429_14_290;signature_desc=Borrelia lipoprotein;Name=PF00921;status=T;Dbxref="InterPro:IPR000680"
gene-bhDAH_001361 (	lp27	pseudo	gamma	Pfam	protein_matc	12	89	4.3E-28	+	.	Target=null 12 89;ID=match$1200_12_89;signature_desc=Borrelia lipoprotein;Name=PF00921;status=T;Dbxref="InterPro:IPR000680"
gene-bhDAH_001361 (	lp27	pseudo		Pfam	protein_matc	9	70	2.8E-09	+	.	Target=null 9 70;ID=match$1569_9_70;signature_desc=Borrelia lipoprotein;Name=PF00921;status=T;Dbxref="InterPro:IPR000680"
gene-bhDAH_001361 (	lp27	pseudo		Pfam	protein_matc	6	131	2.4E-28	+	.	Target=null 6 131;ID=match$2607_6_131;signature_desc=Borrelia lipoprotein;Name=PF00921;status=T;Dbxref="InterPro:IPR000680"
gene-bhDAH_001363 (	lp27	gene	delta	Pfam	protein_matc	13	98	6.6E-14	+	.	Target=null 13 98;ID=match$2798_13_98;signature_desc=Borrelia lipoprotein;Name=PF00921;status=T;Dbxref="InterPro:IPR000680"
gene-bhDAH_001404 (	lp31	gene	gamma	Pfam	protein_matc	13	65	2E-10	+	.	Target=null 13 65;ID=match$1363_13_65;signature_desc=Borrelia lipoprotein;Name=PF00921;status=T;Dbxref="InterPro:IPR000680"
gene-bhDAH_001406 (	lp31	gene	alpha	Pfam	protein_matc	57	354	5.3E-70	+	.	Target=null 57 354;ID=match$1805_57_354;signature_desc=Borrelia lipoprotein;Name=PF00921;status=T;Dbxref="InterPro:IPR000680"
gene-bhDAH_001417 (	lp31	gene	delta	Pfam	protein_matc	43	324	2E-88	+	.	Target=null 43 324;ID=match$2580_43_324;signature_desc=Borrelia lipoprotein;Name=PF00921;status=T;Dbxref="InterPro:IPR000680"
gene-bhDAH_001418 (	lp31	gene	beta	Pfam	protein_matc	47	334	4.6E-48	+	.	Target=null 47 334;ID=match$2044_47_334;signature_desc=Borrelia lipoprotein;Name=PF00921;status=T;Dbxref="InterPro:IPR000680"
gene-bhDAH_001421 (	lp31	pseudo	gamma	Pfam	protein_matc	1	45	5.7E-15	+	.	Target=null 1 45;ID=match$3046_1_45;signature_desc=Borrelia lipoprotein;Name=PF00921;status=T;Dbxref="InterPro:IPR000680"
gene-bhDAH_001421 (	lp31	pseudo		Pfam	protein_matc	1	239	5E-60	+	.	Target=null 1 239;ID=match$959_1_239;signature_desc=Borrelia lipoprotein;Name=PF00921;status=T;Dbxref="InterPro:IPR000680"
gene-bhDAH_001429 (	lp31	pseudo	gamma	Pfam	protein_matc	2	70	2.8E-13	+	.	Target=null 2 70;ID=match$233_2_70;signature_desc=Borrelia lipoprotein;Name=PF00921;status=T;Dbxref="InterPro:IPR000680"
gene-bhDAH_001429 (	lp31	pseudo		Pfam	protein_matc	2	82	1.2E-14	+	.	Target=null 2 82;ID=match$574_2_82;signature_desc=Borrelia lipoprotein;Name=PF00921;status=T;Dbxref="InterPro:IPR000680"
gene-bhDAH_001431 (	lp31	gene	delta	Pfam	protein_matc	1	46	2E-15	+	.	Target=null 1 46;ID=match$766_1_46;signature_desc=Borrelia lipoprotein;Name=PF00921;status=T;Dbxref="InterPro:IPR000680"
gene-bhDAH_001432 (	lp31	pseudo	alpha	Pfam	protein_matc	2	64	1.9E-09	+	.	Target=null 2 64;ID=match$1482_2_64;signature_desc=Borrelia lipoprotein;Name=PF00921;status=T;Dbxref="InterPro:IPR000680"
gene-bhDAH_001433 (	lp31	gene	beta	Pfam	protein_matc	6	46	3.2E-11	+	.	Target=null 6 46;ID=match$2360_6_46;signature_desc=Borrelia lipoprotein;Name=PF00921;status=T;Dbxref="InterPro:IPR000680"
gene-bhDAH_001440 (	lp59	pseudo	beta	Pfam	protein_matc	5	34	0.000013	+	.	Target=null 5 34;ID=match$1250_5_34;signature_desc=Borrelia lipoprotein;Name=PF00921;status=T;Dbxref="InterPro:IPR000680"
gene-bhDAH_001441 (	lp59	pseudo	gamma	Pfam	protein_matc	43	65	0.0000019	+	.	Target=null 43 65;ID=match$1922_43_65;signature_desc=Borrelia lipoprotein;Name=PF00921;status=T;Dbxref="InterPro:IPR000680"
gene-bhDAH_001441 (	lp59	pseudo		Pfam	protein_matc	1	245	9.4E-67	+	.	Target=null 1 245;ID=match$386_1_245;signature_desc=Borrelia lipoprotein;Name=PF00921;status=T;Dbxref="InterPro:IPR000680"
gene-bhDAH_001442 (	lp59	gene	beta	Pfam	protein_matc	47	331	5.9E-45	+	.	Target=null 47 331;ID=match$2922_47_331;signature_desc=Borrelia lipoprotein;Name=PF00921;status=T;Dbxref="InterPro:IPR000680"
gene-bhDAH_001443 (	lp59	gene	beta	Pfam	protein_matc	47	340	2.2E-48	+	.	Target=null 47 340;ID=match$961_47_340;signature_desc=Borrelia lipoprotein;Name=PF00921;status=T;Dbxref="InterPro:IPR000680"
gene-bhDAH_001444 (	lp59	pseudo	gamma	Pfam	protein_matc	1	53	7.8E-08	+	.	Target=null 1 53;ID=match$2458_1_53;signature_desc=Borrelia lipoprotein;Name=PF00921;status=T;Dbxref="InterPro:IPR000680"
gene-bhDAH_001444 (	lp59	pseudo		Pfam	protein_matc	4	89	1.9E-26	+	.	Target=null 4 89;ID=match$1759_4_89;signature_desc=Borrelia lipoprotein;Name=PF00921;status=T;Dbxref="InterPro:IPR000680"
gene-bhDAH_001446 (	lp59	pseudo	alpha	Pfam	protein_matc	57	300	3E-47	+	.	Target=null 57 300;ID=match$849_57_300;signature_desc=Borrelia lipoprotein;Name=PF00921;status=T;Dbxref="InterPro:IPR000680"
gene-bhDAH_001446 (	lp59	pseudo		Pfam	protein_matc	2	50	9.2E-10	+	.	Target=null 2 50;ID=match$2120_2_50;signature_desc=Borrelia lipoprotein;Name=PF00921;status=T;Dbxref="InterPro:IPR000680"
gene-bhDAH_001448 (	lp59	gene	gamma	Pfam	protein_matc	30	322	9.8E-92	+	.	Target=null 30 322;ID=match$2602_30_322;signature_desc=Borrelia lipoprotein;Name=PF00921;status=T;Dbxref="InterPro:IPR000680"

Duplicates between Vsp and Vlp hits. Did not use for Vsp analysis.

Vsp			replicon	gene/pseudo										
gene-bhYOR	001254	orf2	lp23	gene	Pfam	protein	matc	25	193	1.4E-51	+	.	Target=null 25 193;ID=match$608_25_193;signature_desc=Lipoprotein;Name=PF01441;status=T;Dbxref="InterPro:IPR001800"
gene-bhYOR	001256	orf2	lp23	pseudo	Pfam	protein	matc	3	165	4.9E-55	+	.	Target=null 3 165;ID=match$2592_3_165;signature_desc=Lipoprotein;Name=PF01441;status=T;Dbxref="InterPro:IPR001800"
gene-bhYOR	001259	orf2	lp23	gene	Pfam	protein	matc	38	205	1E-62	+	.	Target=null 38 205;ID=match$1182_38_205;signature_desc=Lipoprotein;Name=PF01441;status=T;Dbxref="InterPro:IPR001800"
gene-bhYOR	001265	orf2	lp46	pseudo	Pfam	protein	matc	2	43	1.8E-17	+	.	Target=null 2 43;ID=match$411_2_43;signature_desc=Lipoprotein;Name=PF01441;status=T;Dbxref="InterPro:IPR001800"
gene-bhYOR	001265	orf2	lp46	pseudo	Pfam	protein	matc	13	144	6.9E-33	+	.	Target=null 13 144;ID=match$407_13_144;signature_desc=Lipoprotein;Name=PF01441;status=T;Dbxref="InterPro:IPR001800"
gene-bhYOR	001266	orf2	lp46	gene	Pfam	protein	matc	38	207	5.1E-64	+	.	Target=null 38 207;ID=match$2931_38_207;signature_desc=Lipoprotein;Name=PF01441;status=T;Dbxref="InterPro:IPR001800"
gene-bhYOR	001268	orf2	lp46	pseudo	Pfam	protein	matc	25	188	3.1E-58	+	.	Target=null 25 188;ID=match$2823_25_188;signature_desc=Lipoprotein;Name=PF01441;status=T;Dbxref="InterPro:IPR001800"
gene-bhYOR	001299	orf2	lp46	gene	Pfam	protein	matc	38	206	2.4E-61	+	.	Target=null 38 206;ID=match$639_38_206;signature_desc=Lipoprotein;Name=PF01441;status=T;Dbxref="InterPro:IPR001800"
gene-bhYOR	001308	orf2	lp73	gene	Pfam	protein	matc	38	205	4.2E-63	+	.	Target=null 38 205;ID=match$1719_38_205;signature_desc=Lipoprotein;Name=PF01441;status=T;Dbxref="InterPro:IPR001800"
gene-bhYOR	001311	orf2	lp73	gene	Pfam	protein	matc	1	83	7.9E-23	+	.	Target=null 1 83;ID=match$654_1_83;signature_desc=Lipoprotein;Name=PF01441;status=T;Dbxref="InterPro:IPR001800"
gene-bhYOR	001312	orf2	lp73	pseudo	Pfam	protein	matc	7	172	1.4E-40	+	.	Target=null 7 172;ID=match$2640_7_172;signature_desc=Lipoprotein;Name=PF01441;status=T;Dbxref="InterPro:IPR001800"
gene-bhYOR	001313	orf2	lp73	pseudo	Pfam	protein	matc	43	214	1.2E-53	+	.	Target=null 43 214;ID=match$544_43_214;signature_desc=Lipoprotein;Name=PF01441;status=T;Dbxref="InterPro:IPR001800"
gene-bhYOR	001315	orf2	lp73	gene	Pfam	protein	matc	29	195	1.1E-64	+	.	Target=null 29 195;ID=match$356_29_195;signature_desc=Lipoprotein;Name=PF01441;status=T;Dbxref="InterPro:IPR001800"
gene-bhYOR	001316	orf2	lp73	gene	Pfam	protein	matc	38	205	2.3E-59	+	.	Target=null 38 205;ID=match$1395_38_205;signature_desc=Lipoprotein;Name=PF01441;status=T;Dbxref="InterPro:IPR001800"
gene-bhYOR	001354	orf2	lp73	gene	Pfam	protein	matc	1	116	8.4E-37	+	.	Target=null 1 116;ID=match$817_1_116;signature_desc=Lipoprotein;Name=PF01441;status=T;Dbxref="InterPro:IPR001800"
gene-bhYOR	001361	orf2	lp73	gene	Pfam	protein	matc	38	211	1.1E-43	+	.	Target=null 38 211;ID=match$697_38_211;signature_desc=Lipoprotein;Name=PF01441;status=T;Dbxref="InterPro:IPR001800"
gene-bhYOR	001363	orf2	lp73	pseudo	Pfam	protein	matc	3	171	7.2E-48	+	.	Target=null 3 171;ID=match$2664_3_171;signature_desc=Lipoprotein;Name=PF01441;status=T;Dbxref="InterPro:IPR001800"
gene-bhYOR	001374	orf2	lp73	pseudo	Pfam	protein	matc	2	139	3.8E-51	+	.	Target=null 2 139;ID=match$1956_2_139;signature_desc=Lipoprotein;Name=PF01441;status=T;Dbxref="InterPro:IPR001800"
gene-bhYOR	001376	orf2	lp73	pseudo	Pfam	protein	matc	2	137	3.1E-49	+	.	Target=null 2 137;ID=match$1698_2_137;signature_desc=Lipoprotein;Name=PF01441;status=T;Dbxref="InterPro:IPR001800"
gene-bhYOR	001397	orf2	lp89	gene	Pfam	protein	matc	29	198	6.9E-61	+	.	Target=null 29 198;ID=match$1970_29_198;signature_desc=Lipoprotein;Name=PF01441;status=T;Dbxref="InterPro:IPR001800"
gene-bhYOR	001399	orf2	lp89	gene	Pfam	protein	matc	38	205	3.1E-62	+	.	Target=null 38 205;ID=match$3043_38_205;signature_desc=Lipoprotein;Name=PF01441;status=T;Dbxref="InterPro:IPR001800"
gene-bhYOR	001403	orf2	lp89	gene	Pfam	protein	matc	25	195	3.8E-45	+	.	Target=null 25 195;ID=match$1596_25_195;signature_desc=Lipoprotein;Name=PF01441;status=T;Dbxref="InterPro:IPR001800"
gene-bhYOR	001407	orf2	lp89	pseudo	Pfam	protein	matc	38	71	6.2E-11	+	.	Target=null 38 71;ID=match$288_38_71;signature_desc=Lipoprotein;Name=PF01441;status=T;Dbxref="InterPro:IPR001800"
gene-bhYOR	001407	orf2	lp89	pseudo	Pfam	protein	matc	8	99	5.9E-17	+	.	Target=null 8 99;ID=match$1168_8_99;signature_desc=Lipoprotein;Name=PF01441;status=T;Dbxref="InterPro:IPR001800"
gene-bhYOR	001409	orf2	lp89	pseudo	Pfam	protein	matc	38	82	1.4E-17	+	.	Target=null 38 82;ID=match$1056_38_82;signature_desc=Lipoprotein;Name=PF01441;status=T;Dbxref="InterPro:IPR001800"
gene-bhYOR	001409	orf2	lp89	pseudo	Pfam	protein	matc	1	81	1.7E-24	+	.	Target=null 1 81;ID=match$2048_1_81;signature_desc=Lipoprotein;Name=PF01441;status=T;Dbxref="InterPro:IPR001800"
gene-bhYOR	001409	orf2	lp89	pseudo	Pfam	protein	matc	18	65	1.1E-12	+	.	Target=null 18 65;ID=match$449_18_65;signature_desc=Lipoprotein;Name=PF01441;status=T;Dbxref="InterPro:IPR001800"
gene-bhYOR	001418	orf2	lp89	pseudo	Pfam	protein	matc	3	74	3.4E-28	+	.	Target=null 3 74;ID=match$40_3_74;signature_desc=Lipoprotein;Name=PF01441;status=T;Dbxref="InterPro:IPR001800"
gene-bhYOR	001418	orf2	lp89	pseudo	Pfam	protein	matc	7	44	9.4E-07	+	.	Target=null 7 44;ID=match$43_7_44;signature_desc=Lipoprotein;Name=PF01441;status=T;Dbxref="InterPro:IPR001800"
gene-bhYOR	001445	orf2	lp89	gene	Pfam	protein	matc	38	205	6.4E-68	+	.	Target=null 38 205;ID=match$1670_38_205;signature_desc=Lipoprotein;Name=PF01441;status=T;Dbxref="InterPro:IPR001800"
gene-bhYOR	001447	orf2	lp89	pseudo	Pfam	protein	matc	2	34	7.1E-11	+	.	Target=null 2 34;ID=match$1104_2_34;signature_desc=Lipoprotein;Name=PF01441;status=T;Dbxref="InterPro:IPR001800"
gene-bhYOR	001449	orf2	lp89	gene	Pfam	protein	matc	38	205	7.1E-61	+	.	Target=null 38 205;ID=match$3003_38_205;signature_desc=Lipoprotein;Name=PF01441;status=T;Dbxref="InterPro:IPR001800"
gene-bhYOR	001451	orf2	lp89	gene	Pfam	protein	matc	38	199	2.5E-40	+	.	Target=null 38 199;ID=match$3187_38_199;signature_desc=Lipoprotein;Name=PF01441;status=T;Dbxref="InterPro:IPR001800"
gene-bhYOR	001454	orf2	lp89	pseudo	Pfam	protein	matc	38	138	6.5E-36	+	.	Target=null 38 138;ID=match$2869_38_138;signature_desc=Lipoprotein;Name=PF01441;status=T;Dbxref="InterPro:IPR001800"
gene-bhYOR	001454	orf2	lp89	pseudo	Pfam	protein	matc	6	77	5.1E-18	+	.	Target=null 6 77;ID=match$3226_6_77;signature_desc=Lipoprotein;Name=PF01441;status=T;Dbxref="InterPro:IPR001800"
gene-bhYOR	001469	orf2	lp89	gene	Pfam	protein	matc	38	207	2.2E-61	+	.	Target=null 38 207;ID=match$695_38_207;signature_desc=Lipoprotein;Name=PF01441;status=T;Dbxref="InterPro:IPR001800"
gene-bhYOR	001476	orf2	lp89	pseudo	Pfam	protein	matc	29	169	1.1E-52	+	.	Target=null 29 169;ID=match$2781_29_169;signature_desc=Lipoprotein;Name=PF01441;status=T;Dbxref="InterPro:IPR001800"
gene-bhYOR	001478	orf2	lp89	pseudo	Pfam	protein	matc	16	43	8E-08	+	.	Target=null 16 43;ID=match$220_16_43;signature_desc=Lipoprotein;Name=PF01441;status=T;Dbxref="InterPro:IPR001800"
gene-bhYOR	001479	orf2	lp89	gene	Pfam	protein	matc	1	115	4.5E-24	+	.	Target=null 1 115;ID=match$2336_1_115;signature_desc=Lipoprotein;Name=PF01441;status=T;Dbxref="InterPro:IPR001800"
gene-bhYOR	001481	orf2	lp89	gene	Pfam	protein	matc	29	200	4E-58	+	.	Target=null 29 200;ID=match$2694_29_200;signature_desc=Lipoprotein;Name=PF01441;status=T;Dbxref="InterPro:IPR001800"
gene-bhYOR	001488	orf2	lp93	gene	Pfam	protein	matc	38	206	1.4E-59	+	.	Target=null 38 206;ID=match$286_38_206;signature_desc=Lipoprotein;Name=PF01441;status=T;Dbxref="InterPro:IPR001800"
gene-bhYOR	001499	orf2	lp93	pseudo	Pfam	protein	matc	2	43	1.8E-17	+	.	Target=null 2 43;ID=match$413_2_43;signature_desc=Lipoprotein;Name=PF01441;status=T;Dbxref="InterPro:IPR001800"
gene-bhYOR	001499	orf2	lp93	pseudo	Pfam	protein	matc	13	144	6.9E-33	+	.	Target=null 13 144;ID=match$409_13_144;signature_desc=Lipoprotein;Name=PF01441;status=T;Dbxref="InterPro:IPR001800"
gene-bhYOR	001500	orf2	lp93	gene	Pfam	protein	matc	38	207	5.1E-64	+	.	Target=null 38 207;ID=match$2929_38_207;signature_desc=Lipoprotein;Name=PF01441;status=T;Dbxref="InterPro:IPR001800"
gene-bhYOR	001601	orf3	lp93	gene	Pfam	protein	matc	39	207	4.5E-60	+	.	Target=null 39 207;ID=match$701_39_207;signature_desc=Lipoprotein;Name=PF01441;status=T;Dbxref="InterPro:IPR001800"
gene-bhYOR	001605	orf3	lp93	gene	Pfam	protein	matc	28	194	3.5E-63	+	.	Target=null 28 194;ID=match$2360_28_194;signature_desc=Lipoprotein;Name=PF01441;status=T;Dbxref="InterPro:IPR001800"

Vlp					Subfamily									
gene-bhYOR	001251	orf2	lp23	pseudo	gamma	Pfam	protein	matc	19	126	4.5E-37	+	.	Target=null 19 126;ID=match$737_19_126;signature_desc=Borrelia lipoprotein;Name=PF00921;status=T;Dbxref="InterPro:IPR000680"
gene-bhYOR	001251	orf2	lp23	pseudo		Pfam	protein	matc	9	38	0.000046	+	.	Target=null 9 38;ID=match$279_9_38;signature_desc=Borrelia lipoprotein;Name=PF00921;status=T;Dbxref="InterPro:IPR000680"
gene-bhYOR	001251	orf2	lp23	pseudo		Pfam	protein	matc	9	76	6.9E-10	+	.	Target=null 9 76;ID=match$2190_9_76;signature_desc=Borrelia lipoprotein;Name=PF00921;status=T;Dbxref="InterPro:IPR000680"
gene-bhYOR	001251	orf2	lp23	pseudo		Pfam	protein	matc	13	64	2E-19	+	.	Target=null 13 64;ID=match$24_13_64;signature_desc=Borrelia lipoprotein;Name=PF00921;status=T;Dbxref="InterPro:IPR000680"
gene-bhYOR	001252	orf2	lp23	pseudo	gamma	Pfam	protein	matc	1	118	6E-30	+	.	Target=null 1 118;ID=match$1257_1_118;signature_desc=Borrelia lipoprotein;Name=PF00921;status=T;Dbxref="InterPro:IPR000680"
gene-bhYOR	001253	orf2	lp23	pseudo	delta	Pfam	protein	matc	21	284	3.2E-80	+	.	Target=null 21 284;ID=match$314_21_284;signature_desc=Borrelia lipoprotein;Name=PF00921;status=T;Dbxref="InterPro:IPR000680"
gene-bhYOR	001255	orf2	lp23	gene	delta	Pfam	protein	matc	1	276	1.3E-81	+	.	Target=null 1 276;ID=match$719_1_276;signature_desc=Borrelia lipoprotein;Name=PF00921;status=T;Dbxref="InterPro:IPR000680"
gene-bhYOR	001257	orf2	lp23	pseudo	gamma	Pfam	protein	matc	19	126	4.5E-37	+	.	Target=null 19 126;ID=match$735_19_126;signature_desc=Borrelia lipoprotein;Name=PF00921;status=T;Dbxref="InterPro:IPR000680"
gene-bhYOR	001257	orf2	lp23	pseudo		Pfam	protein	matc	9	38	0.000046	+	.	Target=null 9 38;ID=match$280_9_38;signature_desc=Borrelia lipoprotein;Name=PF00921;status=T;Dbxref="InterPro:IPR000680"
gene-bhYOR	001257	orf2	lp23	pseudo		Pfam	protein	matc	9	76	6.9E-10	+	.	Target=null 9 76;ID=match$2188_9_76;signature_desc=Borrelia lipoprotein;Name=PF00921;status=T;Dbxref="InterPro:IPR000680"
gene-bhYOR	001257	orf2	lp23	pseudo		Pfam	protein	matc	13	82	4.5E-30	+	.	Target=null 13 82;ID=match$565_13_82;signature_desc=Borrelia lipoprotein;Name=PF00921;status=T;Dbxref="InterPro:IPR000680"
gene-bhYOR	001258	orf2	lp23	pseudo	gamma	Pfam	protein	matc	1	61	5.2E-10	+	.	Target=null 1 61;ID=match$2186_1_61;signature_desc=Borrelia lipoprotein;Name=PF00921;status=T;Dbxref="InterPro:IPR000680"
gene-bhYOR	001260	orf2	lp46	gene	delta	Pfam	protein	matc	34	313	6E-85	+	.	Target=null 34 313;ID=match$1068_34_313;signature_desc=Borrelia lipoprotein;Name=PF00921;status=T;Dbxref="InterPro:IPR000680"
gene-bhYOR	001261	orf2	lp46	pseudo	beta	Pfam	protein	matc	10	79	1.1E-19	+	.	Target=null 10 79;ID=match$644_10_79;signature_desc=Borrelia lipoprotein;Name=PF00921;status=T;Dbxref="InterPro:IPR000680"
gene-bhYOR	001262	orf2	lp46	gene	gamma	Pfam	protein	matc	30	314	1.2E-85	+	.	Target=null 30 314;ID=match$363_30_314;signature_desc=Borrelia lipoprotein;Name=PF00921;status=T;Dbxref="InterPro:IPR000680"
gene-bhYOR	001263	orf2	lp46	gene	alpha	Pfam	protein	matc	7	302	8.7E-72	+	.	Target=null 7 302;ID=match$2223_7_302;signature_desc=Borrelia lipoprotein;Name=PF00921;status=T;Dbxref="InterPro:IPR000680"
gene-bhYOR	001264	orf2	lp46	pseudo	beta	Pfam	protein	matc	20	120	1E-13	+	.	Target=null 20 120;ID=match$338_20_120;signature_desc=Borrelia lipoprotein;Name=PF00921;status=T;Dbxref="InterPro:IPR000680"
gene-bhYOR	001264	orf2	lp46	pseudo		Pfam	protein	matc	25	204	1.6E-30	+	.	Target=null 25 204;ID=match$1955_25_204;signature_desc=Borrelia lipoprotein;Name=PF00921;status=T;Dbxref="InterPro:IPR000680"
gene-bhYOR	001267	orf2	lp46	pseudo	delta	Pfam	protein	matc	43	320	1.6E-83	+	.	Target=null 43 320;ID=match$727_43_320;signature_desc=Borrelia lipoprotein;Name=PF00921;status=T;Dbxref="InterPro:IPR000680"
gene-bhYOR	001269	orf2	lp46	pseudo	gamma	Pfam	protein	matc	43	87	1.3E-11	+	.	Target=null 43 87;ID=match$2898_43_87;signature_desc=Borrelia lipoprotein;Name=PF00921;status=T;Dbxref="InterPro:IPR000680"
gene-bhYOR	001269	orf2	lp46	pseudo		Pfam	protein	matc	3	237	1.2E-61	+	.	Target=null 3 237;ID=match$2662_3_237;signature_desc=Borrelia lipoprotein;Name=PF00921;status=T;Dbxref="InterPro:IPR000680"
gene-bhYOR	001303	orf2	lp73	gene	alpha	Pfam	protein	matc	58	343	5.5E-62	+	.	Target=null 58 343;ID=match$1691_58_343;signature_desc=Borrelia lipoprotein;Name=PF00921;status=T;Dbxref="InterPro:IPR000680"
gene-bhYOR	001305	orf2	lp73	pseudo	beta	Pfam	protein	matc	2	190	6.8E-15	+	.	Target=null 2 190;ID=match$1696_2_190;signature_desc=Borrelia lipoprotein;Name=PF00921;status=T;Dbxref="InterPro:IPR000680"
gene-bhYOR	001306	orf2	lp73	gene	alpha	Pfam	protein	matc	23	132	3.5E-28	+	.	Target=null 23 132;ID=match$595_23_132;signature_desc=Borrelia lipoprotein;Name=PF00921;status=T;Dbxref="InterPro:IPR000680"
gene-bhYOR	001307	orf2	lp73	pseudo	gamma	Pfam	protein	matc	3	71	4.4E-23	+	.	Target=null 3 71;ID=match$435_3_71;signature_desc=Borrelia lipoprotein;Name=PF00921;status=T;Dbxref="InterPro:IPR000680"
gene-bhYOR	001314	orf2	lp73	pseudo	gamma	Pfam	protein	matc	1	285	6.2E-85	+	.	Target=null 1 285;ID=match$1311_1_285;signature_desc=Borrelia lipoprotein;Name=PF00921;status=T;Dbxref="InterPro:IPR000680"
gene-bhYOR	001317	orf2	lp73	gene	gamma	Pfam	protein	matc	34	319	1.6E-92	+	.	Target=null 34 319;ID=match$1314_34_319;signature_desc=Borrelia lipoprotein;Name=PF00921;status=T;Dbxref="InterPro:IPR000680"
gene-bhYOR	001318	orf2	lp73	gene	alpha	Pfam	protein	matc	9	130	2.6E-23	+	.	Target=null 9 130;ID=match$831_9_130;signature_desc=Borrelia lipoprotein;Name=PF00921;status=T;Dbxref="InterPro:IPR000680"
gene-bhYOR	001320	orf2	lp73	gene	beta	Pfam	protein	matc	47	334	7.5E-49	+	.	Target=null 47 334;ID=match$38_47_334;signature_desc=Borrelia lipoprotein;Name=PF00921;status=T;Dbxref="InterPro:IPR000680"
gene-bhYOR	001321	orf2	lp73	gene	gamma	Pfam	protein	matc	28	63	2E-10	+	.	Target=null 28 63;ID=match$132_28_63;signature_desc=Borrelia lipoprotein;Name=PF00921;status=T;Dbxref="InterPro:IPR000680"
gene-bhYOR	001328	orf2	lp73	pseudo	gamma	Pfam	protein	matc	3	151	3.7E-36	+	.	Target=null 3 151;ID=match$730_3_151;signature_desc=Borrelia lipoprotein;Name=PF00921;status=T;Dbxref="InterPro:IPR000680"
gene-bhYOR	001328	orf2	lp73	pseudo		Pfam	protein	matc	4	91	6E-15	+	.	Target=null 4 91;ID=match$772_4_91;signature_desc=Borrelia lipoprotein;Name=PF00921;status=T;Dbxref="InterPro:IPR000680"
gene-bhYOR	001338	orf2	lp73	pseudo	delta	Pfam	protein	matc	4	287	6.1E-89	+	.	Target=null 4 287;ID=match$1873_4_287;signature_desc=Borrelia lipoprotein;Name=PF00921;status=T;Dbxref="InterPro:IPR000680"
gene-bhYOR	001339	orf2	lp73	pseudo	gamma	Pfam	protein	matc	2	89	1.1E-27	+	.	Target=null 2 89;ID=match$269_2_89;signature_desc=Borrelia lipoprotein;Name=PF00921;status=T;Dbxref="InterPro:IPR000680"
gene-bhYOR	001352	orf2	lp73	pseudo	gamma	Pfam	protein	matc	14	60	6.2E-13	+	.	Target=null 14 60;ID=match$2321_14_60;signature_desc=Borrelia lipoprotein;Name=PF00921;status=T;Dbxref="InterPro:IPR000680"
gene-bhYOR	001353	orf2	lp73	pseudo	gamma	Pfam	protein	matc	6	38	6.4E-13	+	.	Target=null 6 38;ID=match$3162_6_38;signature_desc=Borrelia lipoprotein;Name=PF00921;status=T;Dbxref="InterPro:IPR000680"
gene-bhYOR	001355	orf2	lp73	gene	alpha	Pfam	protein	matc	59	353	3.9E-72	+	.	Target=null 59 353;ID=match$164_59_353;signature_desc=Borrelia lipoprotein;Name=PF00921;status=T;Dbxref="InterPro:IPR000680"
gene-bhYOR	001356	orf2	lp73	gene	gamma	Pfam	protein	matc	1	236	1.9E-65	+	.	Target=null 1 236;ID=match$1928_1_236;signature_desc=Borrelia lipoprotein;Name=PF00921;status=T;Dbxref="InterPro:IPR000680"
gene-bhYOR	001362	orf2	lp73	pseudo	gamma	Pfam	protein	matc	3	116	1.7E-16	+	.	Target=null 3 116;ID=match$757_3_116;signature_desc=Borrelia lipoprotein;Name=PF00921;status=T;Dbxref="InterPro:IPR000680"
gene-bhYOR	001362	orf2	lp73	pseudo		Pfam	protein	matc	6	111	3.3E-35	+	.	Target=null 6 111;ID=match$3169_6_111;signature_desc=Borrelia lipoprotein;Name=PF00921;status=T;Dbxref="InterPro:IPR000680"
gene-bhYOR	001371	orf2	lp73	pseudo	delta	Pfam	protein	matc	10	107	1.4E-27	+	.	Target=null 10 107;ID=match$2111_10_107;signature_desc=Borrelia lipoprotein;Name=PF00921;status=T;Dbxref="InterPro:IPR000680"
gene-bhYOR	001372	orf2	lp73	gene	alpha	Pfam	protein	matc	4	78	7.8E-24	+	.	Target=null 4 78;ID=match$2080_4_78;signature_desc=Borrelia lipoprotein;Name=PF00921;status=T;Dbxref="InterPro:IPR000680"
gene-bhYOR	001373	orf2	lp73	gene	beta	Pfam	protein	matc	21	315	1.2E-45	+	.	Target=null 21 315;ID=match$1138_21_315;signature_desc=Borrelia lipoprotein;Name=PF00921;status=T;Dbxref="InterPro:IPR000680"
gene-bhYOR	001375	orf2	lp73	pseudo	gamma	Pfam	protein	matc	1	209	4.7E-49	+	.	Target=null 1 209;ID=match$2100_1_209;signature_desc=Borrelia lipoprotein;Name=PF00921;status=T;Dbxref="InterPro:IPR000680"
gene-bhYOR	001375	orf2	lp73	pseudo		Pfam	protein	matc	16	57	5.2E-14	+	.	Target=null 16 57;ID=match$1180_16_57;signature_desc=Borrelia lipoprotein;Name=PF00921;status=T;Dbxref="InterPro:IPR000680"
gene-bhYOR	001377	orf2	lp73	pseudo	delta	Pfam	protein	matc	3	84	9.2E-13	+	.	Target=null 3 84;ID=match$972_3_84;signature_desc=Borrelia lipoprotein;Name=PF00921;status=T;Dbxref="InterPro:IPR000680"
gene-bhYOR	001377	orf2	lp73	pseudo		Pfam	protein	matc	2	182	8.2E-54	+	.	Target=null 2 182;ID=match$765_2_182;signature_desc=Borrelia lipoprotein;Name=PF00921;status=T;Dbxref="InterPro:IPR000680"
gene-bhYOR	001378	orf2	lp73	pseudo	gamma	Pfam	protein	matc	15	48	2.8E-07	+	.	Target=null 15 48;ID=match$2948_15_48;signature_desc=Borrelia lipoprotein;Name=PF00921;status=T;Dbxref="InterPro:IPR000680"
gene-bhYOR	001379	orf2	lp73	pseudo	beta	Pfam	protein	matc	13	97	0.0000034	+	.	Target=null 13 97;ID=match$1784_13_97;signature_desc=Borrelia lipoprotein;Name=PF00921;status=T;Dbxref="InterPro:IPR000680"
gene-bhYOR	001390	orf2	lp89	gene	gamma	Pfam	protein	matc	43	319	1.5E-86	+	.	Target=null 43 319;ID=match$35_43_319;signature_desc=Borrelia lipoprotein;Name=PF00921;status=T;Dbxref="InterPro:IPR000680"
gene-bhYOR	001391	orf2	lp89	gene	beta	Pfam	protein	matc	47	331	5.7E-45	+	.	Target=null 47 331;ID=match$1424_47_331;signature_desc=Borrelia lipoprotein;Name=PF00921;status=T;Dbxref="InterPro:IPR000680"
gene-bhYOR	001392	orf2	lp89	gene	alpha	Pfam	protein	matc	61	353	2.7E-69	+	.	Target=null 61 353;ID=match$1600_61_353;signature_desc=Borrelia lipoprotein;Name=PF00921;status=T;Dbxref="InterPro:IPR000680"
gene-bhYOR	001393	orf2	lp89	gene	beta	Pfam	protein	matc	20	307	5.4E-49	+	.	Target=null 20 307;ID=match$153_20_307;signature_desc=Borrelia lipoprotein;Name=PF00921;status=T;Dbxref="InterPro:IPR000680"
gene-bhYOR	001394	orf2	lp89	pseudo	gamma	Pfam	protein	matc	1	57	2.2E-08	+	.	Target=null 1 57;ID=match$60_1_57;signature_desc=Borrelia lipoprotein;Name=PF00921;status=T;Dbxref="InterPro:IPR000680"
gene-bhYOR	001394	orf2	lp89	pseudo		Pfam	protein	matc	31	65	0.000014	+	.	Target=null 31 65;ID=match$3185_31_65;signature_desc=Borrelia lipoprotein;Name=PF00921;status=T;Dbxref="InterPro:IPR000680"
gene-bhYOR	001394	orf2	lp89	pseudo		Pfam	protein	matc	10	43	0.000012	+	.	Target=null 10 43;ID=match$37_10_43;signature_desc=Borrelia lipoprotein;Name=PF00921;status=T;Dbxref="InterPro:IPR000680"
gene-bhYOR	001394	orf2	lp89	pseudo		Pfam	protein	matc	10	85	9.7E-26	+	.	Target=null 10 85;ID=match$1881_10_85;signature_desc=Borrelia lipoprotein;Name=PF00921;status=T;Dbxref="InterPro:IPR000680"
gene-bhYOR	001396	orf2	lp89	gene	alpha	Pfam	protein	matc	58	355	1E-68	+	.	Target=null 58 355;ID=match$240_58_355;signature_desc=Borrelia lipoprotein;Name=PF00921;status=T;Dbxref="InterPro:IPR000680"
gene-bhYOR	001398	orf2	lp89	pseudo	gamma	Pfam	protein	matc	43	91	2.3E-14	+	.	Target=null 43 91;ID=match$465_43_91;signature_desc=Borrelia lipoprotein;Name=PF00921;status=T;Dbxref="InterPro:IPR000680"
gene-bhYOR	001398	orf2	lp89	pseudo		Pfam	protein	matc	7	237	1.9E-65	+	.	Target=null 7 237;ID=match$2167_7_237;signature_desc=Borrelia lipoprotein;Name=PF00921;status=T;Dbxref="InterPro:IPR000680"
gene-bhYOR	001404	orf2	lp89	pseudo	delta	Pfam	protein	matc	14	305	1.3E-85	+	.	Target=null 14 305;ID=match$513_14_305;signature_desc=Borrelia lipoprotein;Name=PF00921;status=T;Dbxref="InterPro:IPR000680"
gene-bhYOR	001405	orf2	lp89	pseudo	beta	Pfam	protein	matc	20	162	1.3E-17	+	.	Target=null 20 162;ID=match$1364_20_162;signature_desc=Borrelia lipoprotein;Name=PF00921;status=T;Dbxref="InterPro:IPR000680"
gene-bhYOR	001405	orf2	lp89	pseudo		Pfam	protein	matc	78	121	0.0000041	+	.	Target=null 78 121;ID=match$2482_78_121;signature_desc=Borrelia lipoprotein;Name=PF00921;status=T;Dbxref="InterPro:IPR000680"
gene-bhYOR	001406	orf2	lp89	gene	alpha	Pfam	protein	matc	7	304	5.2E-71	+	.	Target=null 7 304;ID=match$2031_7_304;signature_desc=Borrelia lipoprotein;Name=PF00921;status=T;Dbxref="InterPro:IPR000680"
gene-bhYOR	001408	orf2	lp89	pseudo	delta	Pfam	protein	matc	42	315	1.7E-83	+	.	Target=null 42 315;ID=match$1113_42_315;signature_desc=Borrelia lipoprotein;Name=PF00921;status=T;Dbxref="InterPro:IPR000680"
gene-bhYOR	001411	orf2	lp89	gene	beta	Pfam	protein	matc	21	310	1.6E-45	+	.	Target=null 21 310;ID=match$2795_21_310;signature_desc=Borrelia lipoprotein;Name=PF00921;status=T;Dbxref="InterPro:IPR000680"
gene-bhYOR	001412	orf2	lp89	pseudo	alpha	Pfam	protein	matc	58	358	4E-63	+	.	Target=null 58 358;ID=match$2836_58_358;signature_desc=Borrelia lipoprotein;Name=PF00921;status=T;Dbxref="InterPro:IPR000680"
gene-bhYOR	001413	orf2	lp89	gene	delta	Pfam	protein	matc	43	326	1.6E-83	+	.	Target=null 43 326;ID=match$2732_43_326;signature_desc=Borrelia lipoprotein;Name=PF00921;status=T;Dbxref="InterPro:IPR000680"
gene-bhYOR	001414	orf2	lp89	gene	beta	Pfam	protein	matc	55	345	2E-41	+	.	Target=null 55 345;ID=match$2050_55_345;signature_desc=Borrelia lipoprotein;Name=PF00921;status=T;Dbxref="InterPro:IPR000680"
gene-bhYOR	001415	orf2	lp89	pseudo	delta	Pfam	protein	matc	26	301	5.6E-83	+	.	Target=null 26 301;ID=match$2525_26_301;signature_desc=Borrelia lipoprotein;Name=PF00921;status=T;Dbxref="InterPro:IPR000680"
gene-bhYOR	001423	orf2	lp89	pseudo	gamma	Pfam	protein	matc	42	73	1.1E-07	+	.	Target=null 42 73;ID=match$3215_42_73;signature_desc=Borrelia lipoprotein;Name=PF00921;status=T;Dbxref="InterPro:IPR000680"
gene-bhYOR	001423	orf2	lp89	pseudo		Pfam	protein	matc	5	121	3.2E-24	+	.	Target=null 5 121;ID=match$1778_5_121;signature_desc=Borrelia lipoprotein;Name=PF00921;status=T;Dbxref="InterPro:IPR000680"
gene-bhYOR	001424	orf2	lp89	gene	beta	Pfam	protein	matc	20	307	2.4E-44	+	.	Target=null 20 307;ID=match$2008_20_307;signature_desc=Borrelia lipoprotein;Name=PF00921;status=T;Dbxref="InterPro:IPR000680"
gene-bhYOR	001425	orf2	lp89	gene	alpha	Pfam	protein	matc	60	355	1.2E-66	+	.	Target=null 60 355;ID=match$2407_60_355;signature_desc=Borrelia lipoprotein;Name=PF00921;status=T;Dbxref="InterPro:IPR000680"
gene-bhYOR	001429	orf2	lp89	pseudo	gamma	Pfam	protein	matc	4	35	2.3E-10	+	.	Target=null 4 35;ID=match$663_4_35;signature_desc=Borrelia lipoprotein;Name=PF00921;status=T;Dbxref="InterPro:IPR000680"
gene-bhYOR	001430	orf2	lp89	gene	delta	Pfam	protein	matc	43	323	5.3E-89	+	.	Target=null 43 323;ID=match$1266_43_323;signature_desc=Borrelia lipoprotein;Name=PF00921;status=T;Dbxref="InterPro:IPR000680"
gene-bhYOR	001431	orf2	lp89	pseudo	gamma	Pfam	protein	matc	5	31	0.000025	+	.	Target=null 5 31;ID=match$1829_5_31;signature_desc=Borrelia lipoprotein;Name=PF00921;status=T;Dbxref="InterPro:IPR000680"
gene-bhYOR	001439	orf2	lp89	pseudo	delta	Pfam	protein	matc	3	39	1.7E-12	+	.	Target=null 3 39;ID=match$1587_3_39;signature_desc=Borrelia lipoprotein;Name=PF00921;status=T;Dbxref="InterPro:IPR000680"
gene-bhYOR	001440	orf2	lp89	pseudo	beta	Pfam	protein	matc	1	48	8.7E-12	+	.	Target=null 1 48;ID=match$108_1_48;signature_desc=Borrelia lipoprotein;Name=PF00921;status=T;Dbxref="InterPro:IPR000680"
gene-bhYOR	001441	orf2	lp89	gene	delta	Pfam	protein	matc	11	55	3.2E-08	+	.	Target=null 11 55;ID=match$452_11_55;signature_desc=Borrelia lipoprotein;Name=PF00921;status=T;Dbxref="InterPro:IPR000680"
gene-bhYOR	001443	orf2	lp89	pseudo	gamma	Pfam	protein	matc	4	64	2.5E-11	+	.	Target=null 4 64;ID=match$2522_4_64;signature_desc=Borrelia lipoprotein;Name=PF00921;status=T;Dbxref="InterPro:IPR000680"
gene-bhYOR	001444	orf2	lp89	gene	gamma	Pfam	protein	matc	2	94	1.5E-17	+	.	Target=null 2 94;ID=match$2681_2_94;signature_desc=Borrelia lipoprotein;Name=PF00921;status=T;Dbxref="InterPro:IPR000680"
gene-bhYOR	001450	orf2	lp89	pseudo	gamma	Pfam	protein	matc	34	56	0.000011	+	.	Target=null 34 56;ID=match$1903_34_56;signature_desc=Borrelia lipoprotein;Name=PF00921;status=T;Dbxref="InterPro:IPR000680"
gene-bhYOR	001450	orf2	lp89	pseudo		Pfam	protein	matc	1	235	2.9E-60	+	.	Target=null 1 235;ID=match$1614_1_235;signature_desc=Borrelia lipoprotein;Name=PF00921;status=T;Dbxref="InterPro:IPR000680"
gene-bhYOR	001452	orf2	lp89	pseudo	gamma	Pfam	protein	matc	3	53	3.9E-18	+	.	Target=null 3 53;ID=match$1001_3_53;signature_desc=Borrelia lipoprotein;Name=PF00921;status=T;Dbxref="InterPro:IPR000680"
gene-bhYOR	001453	orf2	lp89	gene	gamma	Pfam	protein	matc	8	60	0.000022	+	.	Target=null 8 60;ID=match$1286_8_60;signature_desc=Borrelia lipoprotein;Name=PF00921;status=T;Dbxref="InterPro:IPR000680"
gene-bhYOR	001455	orf2	lp89	gene	gamma	Pfam	protein	matc	28	63	2.3E-10	+	.	Target=null 28 63;ID=match$2890_28_63;signature_desc=Borrelia lipoprotein;Name=PF00921;status=T;Dbxref="InterPro:IPR000680"
gene-bhYOR	001464	orf2	lp89	pseudo	delta	Pfam	protein	matc	1	171	1.7E-45	+	.	Target=null 1 171;ID=match$1813_1_171;signature_desc=Borrelia lipoprotein;Name=PF00921;status=T;Dbxref="InterPro:IPR000680"
gene-bhYOR	001465	orf2	lp89	pseudo	gamma	Pfam	protein	matc	6	38	6.4E-13	+	.	Target=null 6 38;ID=match$1693_6_38;signature_desc=Borrelia lipoprotein;Name=PF00921;status=T;Dbxref="InterPro:IPR000680"
gene-bhYOR	001470	orf2	lp89	gene	gamma	Pfam	protein	matc	4	96	1.1E-20	+	.	Target=null 4 96;ID=match$3035_4_96;signature_desc=Borrelia lipoprotein;Name=PF00921;status=T;Dbxref="InterPro:IPR000680"
gene-bhYOR	001471	orf2	lp89	pseudo	gamma	Pfam	protein	matc	1	29	4.8E-10	+	.	Target=null 1 29;ID=match$1673_1_29;signature_desc=Borrelia lipoprotein;Name=PF00921;status=T;Dbxref="InterPro:IPR000680"
gene-bhYOR	001472	orf2	lp89	pseudo	delta	Pfam	protein	matc	4	164	1.3E-38	+	.	Target=null 4 164;ID=match$708_4_164;signature_desc=Borrelia lipoprotein;Name=PF00921;status=T;Dbxref="InterPro:IPR000680"
gene-bhYOR	001472	orf2	lp89	pseudo		Pfam	protein	matc	17	56	5.6E-12	+	.	Target=null 17 56;ID=match$2400_17_56;signature_desc=Borrelia lipoprotein;Name=PF00921;status=T;Dbxref="InterPro:IPR000680"
gene-bhYOR	001473	orf2	lp89	gene	alpha	Pfam	protein	matc	61	350	9.4E-71	+	.	Target=null 61 350;ID=match$1959_61_350;signature_desc=Borrelia lipoprotein;Name=PF00921;status=T;Dbxref="InterPro:IPR000680"
gene-bhYOR	001474	orf2	lp89	gene	beta	Pfam	protein	matc	20	308	3.7E-45	+	.	Target=null 20 308;ID=match$1553_20_308;signature_desc=Borrelia lipoprotein;Name=PF00921;status=T;Dbxref="InterPro:IPR000680"
gene-bhYOR	001475	orf2	lp89	gene	delta	Pfam	protein	matc	20	302	7.2E-85	+	.	Target=null 20 302;ID=match$2601_20_302;signature_desc=Borrelia lipoprotein;Name=PF00921;status=T;Dbxref="InterPro:IPR000680"
gene-bhYOR	001477	orf2	lp89	gene	delta	Pfam	protein	matc	30	315	3.1E-86	+	.	Target=null 30 315;ID=match$1223_30_315;signature_desc=Borrelia lipoprotein;Name=PF00921;status=T;Dbxref="InterPro:IPR000680"
gene-bhYOR	001480	orf2	lp89	pseudo	gamma	Pfam	protein	matc	7	30	0.0000039	+	.	Target=null 7 30;ID=match$237_7_30;signature_desc=Borrelia lipoprotein;Name=PF00921;status=T;Dbxref="InterPro:IPR000680"
gene-bhYOR	001480	orf2	lp89	pseudo		Pfam	protein	matc	3	270	4.5E-74	+	.	Target=null 3 270;ID=match$3126_3_270;signature_desc=Borrelia lipoprotein;Name=PF00921;status=T;Dbxref="InterPro:IPR000680"
gene-bhYOR	001482	orf2	lp89	gene	alpha	Pfam	protein	matc	57	348	5.1E-66	+	.	Target=null 57 348;ID=match$2449_57_348;signature_desc=Borrelia lipoprotein;Name=PF00921;status=T;Dbxref="InterPro:IPR000680"
gene-bhYOR	001483	orf2	lp89	pseudo	beta	Pfam	protein	matc	34	77	0.0000086	+	.	Target=null 34 77;ID=match$2953_34_77;signature_desc=Borrelia lipoprotein;Name=PF00921;status=T;Dbxref="InterPro:IPR000680"
gene-bhYOR	001483	orf2	lp89	pseudo		Pfam	protein	matc	3	168	2.3E-33	+	.	Target=null 3 168;ID=match$3268_3_168;signature_desc=Borrelia lipoprotein;Name=PF00921;status=T;Dbxref="InterPro:IPR000680"
gene-bhYOR	001484	orf2	lp89	pseudo	gamma	Pfam	protein	matc	3	37	0.0000087	+	.	Target=null 3 37;ID=match$3069_3_37;signature_desc=Borrelia lipoprotein;Name=PF00921;status=T;Dbxref="InterPro:IPR000680"
gene-bhYOR	001485	orf2	lp89	gene	beta	Pfam	protein	matc	22	240	2.3E-37	+	.	Target=null 22 240;ID=match$2956_22_240;signature_desc=Borrelia lipoprotein;Name=PF00921;status=T;Dbxref="InterPro:IPR000680"
gene-bhYOR	001487	orf2	lp93	pseudo	delta	Pfam	protein	matc	14	298	2.4E-87	+	.	Target=null 14 298;ID=match$786_14_298;signature_desc=Borrelia lipoprotein;Name=PF00921;status=T;Dbxref="InterPro:IPR000680"
gene-bhYOR	001489	orf2	lp93	pseudo	gamma	Pfam	protein	matc	2	37	2.4E-11	+	.	Target=null 2 37;ID=match$2411_2_37;signature_desc=Borrelia lipoprotein;Name=PF00921;status=T;Dbxref="InterPro:IPR000680"
gene-bhYOR	001494	orf2	lp93	gene	delta	Pfam	protein	matc	34	313	5.8E-85	+	.	Target=null 34 313;ID=match$589_34_313;signature_desc=Borrelia lipoprotein;Name=PF00921;status=T;Dbxref="InterPro:IPR000680"
gene-bhYOR	001495	orf2	lp93	pseudo	beta	Pfam	protein	matc	10	79	1.1E-19	+	.	Target=null 10 79;ID=match$646_10_79;signature_desc=Borrelia lipoprotein;Name=PF00921;status=T;Dbxref="InterPro:IPR000680"
gene-bhYOR	001496	orf2	lp93	gene	gamma	Pfam	protein	matc	30	314	1.2E-85	+	.	Target=null 30 314;ID=match$365_30_314;signature_desc=Borrelia lipoprotein;Name=PF00921;status=T;Dbxref="InterPro:IPR000680"
gene-bhYOR	001497	orf2	lp93	gene	alpha	Pfam	protein	matc	7	302	8.7E-72	+	.	Target=null 7 302;ID=match$2221_7_302;signature_desc=Borrelia lipoprotein;Name=PF00921;status=T;Dbxref="InterPro:IPR000680"
gene-bhYOR	001498	orf2	lp93	pseudo	beta	Pfam	protein	matc	20	120	1E-13	+	.	Target=null 20 120;ID=match$336_20_120;signature_desc=Borrelia lipoprotein;Name=PF00921;status=T;Dbxref="InterPro:IPR000680"
gene-bhYOR	001498	orf2	lp93	pseudo		Pfam	protein	matc	25	204	1.6E-30	+	.	Target=null 25 204;ID=match$1953_25_204;signature_desc=Borrelia lipoprotein;Name=PF00921;status=T;Dbxref="InterPro:IPR000680"
gene-bhYOR	001501	orf2	lp93	pseudo	delta	Pfam	protein	matc	14	286	2.2E-85	+	.	Target=null 14 286;ID=match$531_14_286;signature_desc=Borrelia lipoprotein;Name=PF00921;status=T;Dbxref="InterPro:IPR000680"
gene-bhYOR	001602	orf3	lp93	gene	gamma	Pfam	protein	matc	1	275	8.6E-79	+	.	Target=null 1 275;ID=match$3110_1_275;signature_desc=Borrelia lipoprotein;Name=PF00921;status=T;Dbxref="InterPro:IPR000680"
gene-bhYOR	001603	orf3	lp93	gene	alpha	Pfam	protein	matc	7	302	8.7E-72	+	.	Target=null 7 302;ID=match$2219_7_302;signature_desc=Borrelia lipoprotein;Name=PF00921;status=T;Dbxref="InterPro:IPR000680"
gene-bhYOR	001604	orf3	lp93	pseudo	beta	Pfam	protein	matc	20	120	1E-13	+	.	Target=null 20 120;ID=match$334_20_120;signature_desc=Borrelia lipoprotein;Name=PF00921;status=T;Dbxref="InterPro:IPR000680"
gene-bhYOR	001604	orf3	lp93	pseudo		Pfam	protein	matc	25	204	1.6E-30	+	.	Target=null 25 204;ID=match$1823_25_204;signature_desc=Borrelia lipoprotein;Name=PF00921;status=T;Dbxref="InterPro:IPR000680"
gene-bhYOR	001606	orf3	lp93	pseudo	delta	Pfam	protein	matc	8	99	1.1E-27	+	.	Target=null 8 99;ID=match$3190_8_99;signature_desc=Borrelia lipoprotein;Name=PF00921;status=T;Dbxref="InterPro:IPR000680"
gene-bhYOR	001606	orf3	lp93	pseudo		Pfam	protein	matc	28	84	1.9E-07	+	.	Target=null 28 84;ID=match$3032_28_84;signature_desc=Borrelia lipoprotein;Name=PF00921;status=T;Dbxref="InterPro:IPR000680"
gene-bhYOR	001606	orf3	lp93	pseudo		Pfam	protein	matc	2	82	6.8E-22	+	.	Target=null 2 82;ID=match$238_2_82;signature_desc=Borrelia lipoprotein;Name=PF00921;status=T;Dbxref="InterPro:IPR000680"
gene-bhYOR	001606	orf3	lp93	pseudo		Pfam	protein	matc	2	41	2E-12	+	.	Target=null 2 41;ID=match$2410_2_41;signature_desc=Borrelia lipoprotein;Name=PF00921;status=T;Dbxref="InterPro:IPR000680"

Vsp			replicon	gene/pseudo										
gene-bcCo53	001291	c	lp20	gene		Pfam	protein	m	44	210	2.00E-59	+	.	Target=null 44 210;ID=match$761_44_210;signature_desc=Lipoprotein;Name=PF01441;status=T;Dbxref="InterPro:IPR001800"
gene-bcCo53	001358	c	Lp24-2	gene		Pfam	protein	m	50	220	4.40E-35	+	.	Target=null 50 220;ID=match$2107_50_220;signature_desc=Lipoprotein;Name=PF01441;status=T;Dbxref="InterPro:IPR001800"
gene-bcCo53	001519	c	lp31	gene		Pfam	protein	m	44	210	5.80E-39	+	.	Target=null 44 210;ID=match$985_44_210;signature_desc=Lipoprotein;Name=PF01441;status=T;Dbxref="InterPro:IPR001800"
gene-bcCo53	001542	c	lp32	gene		Pfam	protein	m	39	211	6.60E-48	+	.	Target=null 39 211;ID=match$2664_39_211;signature_desc=Lipoprotein;Name=PF01441;status=T;Dbxref="InterPro:IPR001800"
gene-bcCo53	001548	c	lp32	gene		Pfam	protein	m	47	209	8.00E-42	+	.	Target=null 47 209;ID=match$219_47_209;signature_desc=Lipoprotein;Name=PF01441;status=T;Dbxref="InterPro:IPR001800"
gene-bcCo53	001553	c	lp32	gene		Pfam	protein	m	48	215	2.80E-41	+	.	Target=null 48 215;ID=match$3114_48_215;signature_desc=Lipoprotein;Name=PF01441;status=T;Dbxref="InterPro:IPR001800"
gene-bcCo53	001554	c	lp32	gene		Pfam	protein	m	44	208	1.00E-51	+	.	Target=null 44 208;ID=match$2497_44_208;signature_desc=Lipoprotein;Name=PF01441;status=T;Dbxref="InterPro:IPR001800"
gene-bcCo53	001556	c	lp32	gene		Pfam	protein	m	39	210	1.70E-52	+	.	Target=null 39 210;ID=match$2418_39_210;signature_desc=Lipoprotein;Name=PF01441;status=T;Dbxref="InterPro:IPR001800"
gene-bcCo53	001603	c	lp34	gene		Pfam	protein	m	44	214	2.00E-43	+	.	Target=null 44 214;ID=match$2923_44_214;signature_desc=Lipoprotein;Name=PF01441;status=T;Dbxref="InterPro:IPR001800"
gene-bcCo53	001639	c	lp38	gene		Pfam	protein	m	44	214	4.70E-43	+	.	Target=null 44 214;ID=match$1934_44_214;signature_desc=Lipoprotein;Name=PF01441;status=T;Dbxref="InterPro:IPR001800"
gene-bcCo53	001642	c	lp38	pseudo		Pfam	protein	m	1	36	5.40E-07	+	.	Target=null 1 36;ID=match$2054_1_36;signature_desc=Lipoprotein;Name=PF01441;status=T;Dbxref="InterPro:IPR001800"
gene-bcCo53	001644	c	lp38	gene		Pfam	protein	m	47	216	1.50E-36	+	.	Target=null 47 216;ID=match$599_47_216;signature_desc=Lipoprotein;Name=PF01441;status=T;Dbxref="InterPro:IPR001800"
gene-bcCo53	001690	c	lp39	gene		Pfam	protein	m	5	95	2.20E-10	+	.	Target=null 5 95;ID=match$1138_5_95;signature_desc=Lipoprotein;Name=PF01441;status=T;Dbxref="InterPro:IPR001800"
Vlp					Subfamily									
gene-bcCo53	000002	c	chromosome	gene	beta	Pfam	protein	m	275	497	5.90E-27	+	.	Target=null 275 497;ID=match$2487_275_497;signature_desc=Borrelia lipoprotein;Name=PF00921;status=T;Dbxref="InterPro:IPR000680"
gene-bcCo53	001279	c	lp20	gene	beta	Pfam	protein	m	9	235	1.60E-19	+	.	Target=null 9 235;ID=match$2104_9_235;signature_desc=Borrelia lipoprotein;Name=PF00921;status=T;Dbxref="InterPro:IPR000680"
gene-bcCo53	001285	c	lp20	gene	delta	Pfam	protein	m	49	326	3.40E-73	+	.	Target=null 49 326;ID=match$1559_49_326;signature_desc=Borrelia lipoprotein;Name=PF00921;status=T;Dbxref="InterPro:IPR000680"
gene-bcCo53	001286	c	lp20	gene	gamma	Pfam	protein	m	49	325	2.60E-84	+	.	Target=null 49 325;ID=match$2448_49_325;signature_desc=Borrelia lipoprotein;Name=PF00921;status=T;Dbxref="InterPro:IPR000680"
gene-bcCo53	001287	c	lp20	gene	gamma	Pfam	protein	m	49	334	2.30E-79	+	.	Target=null 49 334;ID=match$586_49_334;signature_desc=Borrelia lipoprotein;Name=PF00921;status=T;Dbxref="InterPro:IPR000680"
gene-bcCo53	001288	c	lp20	gene	gamma	Pfam	protein	m	49	342	4.20E-85	+	.	Target=null 49 342;ID=match$992_49_342;signature_desc=Borrelia lipoprotein;Name=PF00921;status=T;Dbxref="InterPro:IPR000680"
gene-bcCo53	001289	c	lp20	gene	gamma	Pfam	protein	m	49	342	4.20E-85	+	.	Target=null 49 342;ID=match$3118_49_342;signature_desc=Borrelia lipoprotein;Name=PF00921;status=T;Dbxref="InterPro:IPR000680"
gene-bcCo53	001290	c	lp20	gene	delta	Pfam	protein	m	49	330	9.00E-88	+	.	Target=null 49 330;ID=match$1799_49_330;signature_desc=Borrelia lipoprotein;Name=PF00921;status=T;Dbxref="InterPro:IPR000680"
gene-bcCo53	001292	c	lp20	gene	delta	Pfam	protein	m	49	328	1.70E-80	+	.	Target=null 49 328;ID=match$2955_49_328;signature_desc=Borrelia lipoprotein;Name=PF00921;status=T;Dbxref="InterPro:IPR000680"
gene-bcCo53	001293	c	lp20	gene	gamma	Pfam	protein	m	109	180	5.30E-17	+	.	Target=null 109 180;ID=match$1320_109_180;signature_desc=Borrelia lipoprotein;Name=PF00921;status=T;Dbxref="InterPro:IPR000680"
gene-bcCo53	001293	c	lp20			Pfam	protein	m	1	100	1.30E-09	+	.	Target=null 1 100;ID=match$1320_1_100;signature_desc=Borrelia lipoprotein;Name=PF00921;status=T;Dbxref="InterPro:IPR000680"
gene-bcCo53	001294	c	lp20	gene	gamma	Pfam	protein	m	6	38	3.30E-11	+	.	Target=null 6 38;ID=match$1893_6_38;signature_desc=Borrelia lipoprotein;Name=PF00921;status=T;Dbxref="InterPro:IPR000680"
gene-bcCo53	001306	c	lp23	gene	gamma	Pfam	protein	m	49	328	2.70E-89	+	.	Target=null 49 328;ID=match$3338_49_328;signature_desc=Borrelia lipoprotein;Name=PF00921;status=T;Dbxref="InterPro:IPR000680"
gene-bcCo53	001307	c	lp23	gene	beta	Pfam	protein	m	1	164	2.10E-24	+	.	Target=null 1 164;ID=match$201_1_164;signature_desc=Borrelia lipoprotein;Name=PF00921;status=T;Dbxref="InterPro:IPR000680"
gene-bcCo53	001308	c	lp23	gene	beta	Pfam	protein	m	1	275	6.60E-37	+	.	Target=null 1 275;ID=match$1299_1_275;signature_desc=Borrelia lipoprotein;Name=PF00921;status=T;Dbxref="InterPro:IPR000680"
gene-bcCo53	001309	c	lp23	gene	gamma	Pfam	protein	m	51	338	2.70E-82	+	.	Target=null 51 338;ID=match$1653_51_338;signature_desc=Borrelia lipoprotein;Name=PF00921;status=T;Dbxref="InterPro:IPR000680"
gene-bcCo53	001310	c	lp23	gene	gamma	Pfam	protein	m	48	290	2.50E-52	+	.	Target=null 48 290;ID=match$2462_48_290;signature_desc=Borrelia lipoprotein;Name=PF00921;status=T;Dbxref="InterPro:IPR000680"
gene-bcCo53	001310	c	lp23			Pfam	protein	m	1	58	7.00E-17	+	.	Target=null 1 58;ID=match$2696_1_58;signature_desc=Borrelia lipoprotein;Name=PF00921;status=T;Dbxref="InterPro:IPR000680"
gene-bcCo53	001311	c	lp23	gene	alpha	Pfam	protein	m	42	338	1.60E-57	+	.	Target=null 42 338;ID=match$2076_42_338;signature_desc=Borrelia lipoprotein;Name=PF00921;status=T;Dbxref="InterPro:IPR000680"
gene-bcCo53	001312	c	lp23	gene	delta	Pfam	protein	m	49	333	6.20E-61	+	.	Target=null 49 333;ID=match$2868_49_333;signature_desc=Borrelia lipoprotein;Name=PF00921;status=T;Dbxref="InterPro:IPR000680"
gene-bcCo53	001313	c	lp23	pseudo	alpha	Pfam	protein	m	50	75	3.80E-06	+	.	Target=null 50 75;ID=match$1968_50_75;signature_desc=Borrelia lipoprotein;Name=PF00921;status=T;Dbxref="InterPro:IPR000680"
gene-bcCo53	001313	c	lp23			Pfam	protein	m	5	139	1.00E-14	+	.	Target=null 5 139;ID=match$1544_5_139;signature_desc=Borrelia lipoprotein;Name=PF00921;status=T;Dbxref="InterPro:IPR000680"
gene-bcCo53	001322	c	Lp24-1	gene	delta	Pfam	protein	m	44	74	2.30E-07	+	.	Target=null 44 74;ID=match$2193_44_74;signature_desc=Borrelia lipoprotein;Name=PF00921;status=T;Dbxref="InterPro:IPR000680"
gene-bcCo53	001323	c	Lp24-1	gene	beta	Pfam	protein	m	50	331	8.70E-48	+	.	Target=null 50 331;ID=match$2461_50_331;signature_desc=Borrelia lipoprotein;Name=PF00921;status=T;Dbxref="InterPro:IPR000680"
gene-bcCo53	001324	c	Lp24-1	gene	gamma	Pfam	protein	m	7	73	5.20E-20	+	.	Target=null 7 73;ID=match$2242_7_73;signature_desc=Borrelia lipoprotein;Name=PF00921;status=T;Dbxref="InterPro:IPR000680"
gene-bcCo53	001325	c	Lp24-1	gene	gamma	Pfam	protein	m	3	126	5.60E-25	+	.	Target=null 3 126;ID=match$1573_3_126;signature_desc=Borrelia lipoprotein;Name=PF00921;status=T;Dbxref="InterPro:IPR000680"
gene-bcCo53	001326	c	Lp24-1	pseudo	gamma	Pfam	protein	m	1	45	6.10E-09	+	.	Target=null 1 45;ID=match$2308_1_45;signature_desc=Borrelia lipoprotein;Name=PF00921;status=T;Dbxref="InterPro:IPR000680"
gene-bcCo53	001328	c	Lp24-1	gene	alpha	Pfam	protein	m	17	307	5.60E-66	+	.	Target=null 17 307;ID=match$2595_17_307;signature_desc=Borrelia lipoprotein;Name=PF00921;status=T;Dbxref="InterPro:IPR000680"
gene-bcCo53	001329	c	Lp24-1	gene	gamma	Pfam	protein	m	50	335	3.00E-75	+	.	Target=null 50 335;ID=match$2574_50_335;signature_desc=Borrelia lipoprotein;Name=PF00921;status=T;Dbxref="InterPro:IPR000680"
gene-bcCo53	001330	c	Lp24-1	pseudo	alpha	Pfam	protein	m	50	85	2.10E-08	+	.	Target=null 50 85;ID=match$2337_50_85;signature_desc=Borrelia lipoprotein;Name=PF00921;status=T;Dbxref="InterPro:IPR000680"
gene-bcCo53	001330	c	Lp24-1			Pfam	protein	m	3	173	4.00E-17	+	.	Target=null 3 173;ID=match$216_3_173;signature_desc=Borrelia lipoprotein;Name=PF00921;status=T;Dbxref="InterPro:IPR000680"
gene-bcCo53	001330	c	Lp24-1			Pfam	protein	m	8	84	6.50E-26	+	.	Target=null 8 84;ID=match$1507_8_84;signature_desc=Borrelia lipoprotein;Name=PF00921;status=T;Dbxref="InterPro:IPR000680"
gene-bcCo53	001331	c	Lp24-1	pseudo	beta	Pfam	protein	m	49	234	3.80E-21	+	.	Target=null 49 234;ID=match$1950_49_234;signature_desc=Borrelia lipoprotein;Name=PF00921;status=T;Dbxref="InterPro:IPR000680"
gene-bcCo53	001331	c	Lp24-1			Pfam	protein	m	2	72	1.30E-06	+	.	Target=null 2 72;ID=match$1709_2_72;signature_desc=Borrelia lipoprotein;Name=PF00921;status=T;Dbxref="InterPro:IPR000680"
gene-bcCo53	001331	c	Lp24-1			Pfam	protein	m	8	50	5.50E-12	+	.	Target=null 8 50;ID=match$182_8_50;signature_desc=Borrelia lipoprotein;Name=PF00921;status=T;Dbxref="InterPro:IPR000680"
gene-bcCo53	001332	c	Lp24-1	gene	beta	Pfam	protein	m	50	331	1.40E-40	+	.	Target=null 50 331;ID=match$534_50_331;signature_desc=Borrelia lipoprotein;Name=PF00921;status=T;Dbxref="InterPro:IPR000680"
gene-bcCo53	001333	c	Lp24-1	gene	gamma	Pfam	protein	m	50	329	5.20E-68	+	.	Target=null 50 329;ID=match$995_50_329;signature_desc=Borrelia lipoprotein;Name=PF00921;status=T;Dbxref="InterPro:IPR000680"
gene-bcCo53	001334	c	Lp24-1	gene	delta	Pfam	protein	m	49	330	2.10E-78	+	.	Target=null 49 330;ID=match$1698_49_330;signature_desc=Borrelia lipoprotein;Name=PF00921;status=T;Dbxref="InterPro:IPR000680"
gene-bcCo53	001350	c	Lp24-2	gene	delta	Pfam	protein	m	45	286	6.00E-62	+	.	Target=null 45 286;ID=match$912_45_286;signature_desc=Borrelia lipoprotein;Name=PF00921;status=T;Dbxref="InterPro:IPR000680"
gene-bcCo53	001351	c	Lp24-2	gene	gamma	Pfam	protein	m	18	48	1.60E-09	+	.	Target=null 18 48;ID=match$2636_18_48;signature_desc=Borrelia lipoprotein;Name=PF00921;status=T;Dbxref="InterPro:IPR000680"
gene-bcCo53	001352	c	Lp24-2	gene	gamma	Pfam	protein	m	6	70	2.70E-10	+	.	Target=null 6 70;ID=match$28_6_70;signature_desc=Borrelia lipoprotein;Name=PF00921;status=T;Dbxref="InterPro:IPR000680"
gene-bcCo53	001354	c	Lp24-2	gene	gamma	Pfam	protein	m	49	344	6.90E-78	+	.	Target=null 49 344;ID=match$1916_49_344;signature_desc=Borrelia lipoprotein;Name=PF00921;status=T;Dbxref="InterPro:IPR000680"
gene-bcCo53	001355	c	Lp24-2	pseudo	alpha	Pfam	protein	m	6	45	1.60E-09	+	.	Target=null 6 45;ID=match$8_6_45;signature_desc=Borrelia lipoprotein;Name=PF00921;status=T;Dbxref="InterPro:IPR000680"
gene-bcCo53	001355	c	Lp24-2			Pfam	protein	m	5	204	1.10E-38	+	.	Target=null 5 204;ID=match$2990_5_204;signature_desc=Borrelia lipoprotein;Name=PF00921;status=T;Dbxref="InterPro:IPR000680"
gene-bcCo53	001356	c	Lp24-2	gene	gamma	Pfam	protein	m	49	338	2.00E-77	+	.	Target=null 49 338;ID=match$2286_49_338;signature_desc=Borrelia lipoprotein;Name=PF00921;status=T;Dbxref="InterPro:IPR000680"
gene-bcCo53	001357	c	Lp24-2	gene	beta	Pfam	protein	m	50	334	8.80E-50	+	.	Target=null 50 334;ID=match$1958_50_334;signature_desc=Borrelia lipoprotein;Name=PF00921;status=T;Dbxref="InterPro:IPR000680"
gene-bcCo53	001359	c	Lp24-2	gene	beta	Pfam	protein	m	50	340	2.80E-51	+	.	Target=null 50 340;ID=match$3268_50_340;signature_desc=Borrelia lipoprotein;Name=PF00921;status=T;Dbxref="InterPro:IPR000680"
gene-bcCo53	001360	c	Lp24-2	gene	gamma	Pfam	protein	m	21	309	4.60E-86	+	.	Target=null 21 309;ID=match$1610_21_309;signature_desc=Borrelia lipoprotein;Name=PF00921;status=T;Dbxref="InterPro:IPR000680"
gene-bcCo53	001361	c	Lp24-2	gene	gamma	Pfam	protein	m	252	321	3.70E-27	+	.	Target=null 252 321;ID=match$2443_252_321;signature_desc=Borrelia lipoprotein;Name=PF00921;status=T;Dbxref="InterPro:IPR000680"
gene-bcCo53	001361	c	Lp24-2			Pfam	protein	m	52	249	2.50E-48	+	.	Target=null 52 249;ID=match$2443_52_249;signature_desc=Borrelia lipoprotein;Name=PF00921;status=T;Dbxref="InterPro:IPR000680"
gene-bcCo53	001362	c	Lp24-2	gene	alpha	Pfam	protein	m	49	345	7.20E-64	+	.	Target=null 49 345;ID=match$2405_49_345;signature_desc=Borrelia lipoprotein;Name=PF00921;status=T;Dbxref="InterPro:IPR000680"
gene-bcCo53	001399	c	Lp28-2	gene	beta	Pfam	protein	m	99	353	2.80E-20	+	.	Target=null 99 353;ID=match$1425_99_353;signature_desc=Borrelia lipoprotein;Name=PF00921;status=T;Dbxref="InterPro:IPR000680"
gene-bcCo53	001404	c	Lp28-2	gene	gamma	Pfam	protein	m	49	326	3.40E-73	+	.	Target=null 49 326;ID=match$1561_49_326;signature_desc=Borrelia lipoprotein;Name=PF00921;status=T;Dbxref="InterPro:IPR000680"
gene-bcCo53	001405	c	Lp28-2	gene	gamma	Pfam	protein	m	49	325	2.60E-84	+	.	Target=null 49 325;ID=match$2446_49_325;signature_desc=Borrelia lipoprotein;Name=PF00921;status=T;Dbxref="InterPro:IPR000680"
gene-bcCo53	001406	c	Lp28-2	gene	gamma	Pfam	protein	m	49	334	2.30E-79	+	.	Target=null 49 334;ID=match$588_49_334;signature_desc=Borrelia lipoprotein;Name=PF00921;status=T;Dbxref="InterPro:IPR000680"
gene-bcCo53	001407	c	Lp28-2	gene	gamma	Pfam	protein	m	49	342	4.20E-85	+	.	Target=null 49 342;ID=match$994_49_342;signature_desc=Borrelia lipoprotein;Name=PF00921;status=T;Dbxref="InterPro:IPR000680"
gene-bcCo53	001408	c	Lp28-2	gene	gamma	Pfam	protein	m	49	342	4.20E-85	+	.	Target=null 49 342;ID=match$3116_49_342;signature_desc=Borrelia lipoprotein;Name=PF00921;status=T;Dbxref="InterPro:IPR000680"
gene-bcCo53	001421	c	Lp28-2	gene	beta	Pfam	protein	m	275	497	5.90E-27	+	.	Target=null 275 497;ID=match$2485_275_497;signature_desc=Borrelia lipoprotein;Name=PF00921;status=T;Dbxref="InterPro:IPR000680"
gene-bcCo53	001452	c	Lp29-2	gene	beta	Pfam	protein	m	72	347	4.60E-27	+	.	Target=null 72 347;ID=match$1545_72_347;signature_desc=Borrelia lipoprotein;Name=PF00921;status=T;Dbxref="InterPro:IPR000680"
gene-bcCo53	001459	c	Lp29-2	gene	gamma	Pfam	protein	m	49	336	3.00E-78	+	.	Target=null 49 336;ID=match$1557_49_336;signature_desc=Borrelia lipoprotein;Name=PF00921;status=T;Dbxref="InterPro:IPR000680"
gene-bcCo53	001460	c	Lp29-2	gene	beta	Pfam	protein	m	38	323	2.10E-43	+	.	Target=null 38 323;ID=match$678_38_323;signature_desc=Borrelia lipoprotein;Name=PF00921;status=T;Dbxref="InterPro:IPR000680"
gene-bcCo53	001461	c	Lp29-2	gene	gamma	Pfam	protein	m	50	348	2.80E-74	+	.	Target=null 50 348;ID=match$211_50_348;signature_desc=Borrelia lipoprotein;Name=PF00921;status=T;Dbxref="InterPro:IPR000680"
gene-bcCo53	001462	c	Lp29-2	gene	delta	Pfam	protein	m	49	335	2.70E-83	+	.	Target=null 49 335;ID=match$123_49_335;signature_desc=Borrelia lipoprotein;Name=PF00921;status=T;Dbxref="InterPro:IPR000680"
gene-bcCo53	001463	c	Lp29-2	pseudo	beta	Pfam	protein	m	49	159	5.10E-13	+	.	Target=null 49 159;ID=match$2228_49_159;signature_desc=Borrelia lipoprotein;Name=PF00921;status=T;Dbxref="InterPro:IPR000680"
gene-bcCo53	001463	c	Lp29-2			Pfam	protein	m	23	200	8.50E-27	+	.	Target=null 23 200;ID=match$1422_23_200;signature_desc=Borrelia lipoprotein;Name=PF00921;status=T;Dbxref="InterPro:IPR000680"
gene-bcCo53	001464	c	Lp29-2	gene	delta	Pfam	protein	m	53	342	8.50E-76	+	.	Target=null 53 342;ID=match$328_53_342;signature_desc=Borrelia lipoprotein;Name=PF00921;status=T;Dbxref="InterPro:IPR000680"
gene-bcCo53	001465	c	Lp29-2	gene	gamma	Pfam	protein	m	49	338	2.70E-83	+	.	Target=null 49 338;ID=match$2997_49_338;signature_desc=Borrelia lipoprotein;Name=PF00921;status=T;Dbxref="InterPro:IPR000680"
gene-bcCo53	001466	c	Lp29-2	gene	delta	Pfam	protein	m	48	333	2.30E-59	+	.	Target=null 48 333;ID=match$1621_48_333;signature_desc=Borrelia lipoprotein;Name=PF00921;status=T;Dbxref="InterPro:IPR000680"
gene-bcCo53	001467	c	Lp29-2	gene	gamma	Pfam	protein	m	50	329	7.20E-77	+	.	Target=null 50 329;ID=match$390_50_329;signature_desc=Borrelia lipoprotein;Name=PF00921;status=T;Dbxref="InterPro:IPR000680"
gene-bcCo53	001468	c	Lp29-2	gene	delta	Pfam	protein	m	52	329	9.10E-83	+	.	Target=null 52 329;ID=match$1029_52_329;signature_desc=Borrelia lipoprotein;Name=PF00921;status=T;Dbxref="InterPro:IPR000680"
gene-bcCo53	001469	c	Lp29-2	gene	gamma	Pfam	protein	m	49	332	1.20E-66	+	.	Target=null 49 332;ID=match$3105_49_332;signature_desc=Borrelia lipoprotein;Name=PF00921;status=T;Dbxref="InterPro:IPR000680"
gene-bcCo53	001470	c	Lp29-2	gene	gamma	Pfam	protein	m	49	344	9.50E-82	+	.	Target=null 49 344;ID=match$150_49_344;signature_desc=Borrelia lipoprotein;Name=PF00921;status=T;Dbxref="InterPro:IPR000680"
gene-bcCo53	001477	c	lp30	gene	beta	Pfam	protein	m	75	332	1.40E-26	+	.	Target=null 75 332;ID=match$1108_75_332;signature_desc=Borrelia lipoprotein;Name=PF00921;status=T;Dbxref="InterPro:IPR000680"
gene-bcCo53	001486	c	lp30	pseudo	gamma	Pfam	protein	m	45	334	9.10E-80	+	.	Target=null 45 334;ID=match$1809_45_334;signature_desc=Borrelia lipoprotein;Name=PF00921;status=T;Dbxref="InterPro:IPR000680"
gene-bcCo53	001487	c	lp30	gene	gamma	Pfam	protein	m	49	334	3.70E-71	+	.	Target=null 49 334;ID=match$2004_49_334;signature_desc=Borrelia lipoprotein;Name=PF00921;status=T;Dbxref="InterPro:IPR000680"
gene-bcCo53	001488	c	lp30	gene	delta	Pfam	protein	m	53	341	4.30E-59	+	.	Target=null 53 341;ID=match$2949_53_341;signature_desc=Borrelia lipoprotein;Name=PF00921;status=T;Dbxref="InterPro:IPR000680"
gene-bcCo53	001489	c	lp30	gene	gamma	Pfam	protein	m	52	339	1.40E-71	+	.	Target=null 52 339;ID=match$311_52_339;signature_desc=Borrelia lipoprotein;Name=PF00921;status=T;Dbxref="InterPro:IPR000680"
gene-bcCo53	001490	c	lp30	gene	delta	Pfam	protein	m	50	335	8.30E-61	+	.	Target=null 50 335;ID=match$399_50_335;signature_desc=Borrelia lipoprotein;Name=PF00921;status=T;Dbxref="InterPro:IPR000680"
gene-bcCo53	001491	c	lp30	gene	gamma	Pfam	protein	m	44	335	9.10E-82	+	.	Target=null 44 335;ID=match$2655_44_335;signature_desc=Borrelia lipoprotein;Name=PF00921;status=T;Dbxref="InterPro:IPR000680"
gene-bcCo53	001492	c	lp30	pseudo	gamma	Pfam	protein	m	7	45	3.70E-13	+	.	Target=null 7 45;ID=match$3205_7_45;signature_desc=Borrelia lipoprotein;Name=PF00921;status=T;Dbxref="InterPro:IPR000680"
gene-bcCo53	001493	c	lp30	gene	delta	Pfam	protein	m	49	337	3.60E-58	+	.	Target=null 49 337;ID=match$2162_49_337;signature_desc=Borrelia lipoprotein;Name=PF00921;status=T;Dbxref="InterPro:IPR000680"
gene-bcCo53	001494	c	lp30	gene	delta	Pfam	protein	m	49	327	1.10E-82	+	.	Target=null 49 327;ID=match$298_49_327;signature_desc=Borrelia lipoprotein;Name=PF00921;status=T;Dbxref="InterPro:IPR000680"
gene-bcCo53	001495	c	lp30	gene	delta	Pfam	protein	m	49	336	6.30E-61	+	.	Target=null 49 336;ID=match$1303_49_336;signature_desc=Borrelia lipoprotein;Name=PF00921;status=T;Dbxref="InterPro:IPR000680"
gene-bcCo53	001496	c	lp30	gene	gamma	Pfam	protein	m	49	351	1.30E-70	+	.	Target=null 49 351;ID=match$1228_49_351;signature_desc=Borrelia lipoprotein;Name=PF00921;status=T;Dbxref="InterPro:IPR000680"
gene-bcCo53	001497	c	lp30	pseudo	gamma	Pfam	protein	m	49	93	1.00E-09	+	.	Target=null 49 93;ID=match$1555_49_93;signature_desc=Borrelia lipoprotein;Name=PF00921;status=T;Dbxref="InterPro:IPR000680"
gene-bcCo53	001497	c	lp30			Pfam	protein	m	1	257	1.50E-50	+	.	Target=null 1 257;ID=match$425_1_257;signature_desc=Borrelia lipoprotein;Name=PF00921;status=T;Dbxref="InterPro:IPR000680"
gene-bcCo53	001508	c	lp31	gene	alpha	Pfam	protein	m	5	59	8.30E-17	+	.	Target=null 5 59;ID=match$419_5_59;signature_desc=Borrelia lipoprotein;Name=PF00921;status=T;Dbxref="InterPro:IPR000680"
gene-bcCo53	001509	c	lp31	pseudo	alpha	Pfam	protein	m	3	89	1.80E-09	+	.	Target=null 3 89;ID=match$1868_3_89;signature_desc=Borrelia lipoprotein;Name=PF00921;status=T;Dbxref="InterPro:IPR000680"
gene-bcCo53	001510	c	lp31	gene	alpha	Pfam	protein	m	57	196	6.10E-19	+	.	Target=null 57 196;ID=match$2795_57_196;signature_desc=Borrelia lipoprotein;Name=PF00921;status=T;Dbxref="InterPro:IPR000680"
gene-bcCo53	001511	c	lp31	pseudo	beta	Pfam	protein	m	50	181	5.90E-15	+	.	Target=null 50 181;ID=match$706_50_181;signature_desc=Borrelia lipoprotein;Name=PF00921;status=T;Dbxref="InterPro:IPR000680"
gene-bcCo53	001511	c	lp31			Pfam	protein	m	26	153	1.30E-23	+	.	Target=null 26 153;ID=match$3042_26_153;signature_desc=Borrelia lipoprotein;Name=PF00921;status=T;Dbxref="InterPro:IPR000680"
gene-bcCo53	001512	c	lp31	gene	alpha	Pfam	protein	m	50	350	3.60E-61	+	.	Target=null 50 350;ID=match$1140_50_350;signature_desc=Borrelia lipoprotein;Name=PF00921;status=T;Dbxref="InterPro:IPR000680"
gene-bcCo53	001513	c	lp31	pseudo	beta	Pfam	protein	m	6	275	1.40E-36	+	.	Target=null 6 275;ID=match$3_6_275;signature_desc=Borrelia lipoprotein;Name=PF00921;status=T;Dbxref="InterPro:IPR000680"
gene-bcCo53	001514	c	lp31	gene	delta	Pfam	protein	m	49	329	1.40E-80	+	.	Target=null 49 329;ID=match$160_49_329;signature_desc=Borrelia lipoprotein;Name=PF00921;status=T;Dbxref="InterPro:IPR000680"
gene-bcCo53	001515	c	lp31	gene	beta	Pfam	protein	m	42	333	1.20E-45	+	.	Target=null 42 333;ID=match$2094_42_333;signature_desc=Borrelia lipoprotein;Name=PF00921;status=T;Dbxref="InterPro:IPR000680"
gene-bcCo53	001516	c	lp31	gene	delta	Pfam	protein	m	49	330	3.30E-78	+	.	Target=null 49 330;ID=match$571_49_330;signature_desc=Borrelia lipoprotein;Name=PF00921;status=T;Dbxref="InterPro:IPR000680"
gene-bcCo53	001517	c	lp31	gene	gamma	Pfam	protein	m	49	328	7.10E-85	+	.	Target=null 49 328;ID=match$466_49_328;signature_desc=Borrelia lipoprotein;Name=PF00921;status=T;Dbxref="InterPro:IPR000680"
gene-bcCo53	001518	c	lp31	gene	delta	Pfam	protein	m	49	327	4.20E-80	+	.	Target=null 49 327;ID=match$749_49_327;signature_desc=Borrelia lipoprotein;Name=PF00921;status=T;Dbxref="InterPro:IPR000680"
gene-bcCo53	001520	c	lp31	gene	gamma	Pfam	protein	m	31	328	1.70E-82	+	.	Target=null 31 328;ID=match$246_31_328;signature_desc=Borrelia lipoprotein;Name=PF00921;status=T;Dbxref="InterPro:IPR000680"
gene-bcCo53	001521	c	lp31	gene	delta	Pfam	protein	m	38	315	9.90E-72	+	.	Target=null 38 315;ID=match$55_38_315;signature_desc=Borrelia lipoprotein;Name=PF00921;status=T;Dbxref="InterPro:IPR000680"
gene-bcCo53	001527	c	lp31	gene	gamma	Pfam	protein	m	1	32	8.50E-08	+	.	Target=null 1 32;ID=match$2678_1_32;signature_desc=Borrelia lipoprotein;Name=PF00921;status=T;Dbxref="InterPro:IPR000680"
gene-bcCo53	001543	c	lp32	gene	gamma	Pfam	protein	m	49	340	1.00E-81	+	.	Target=null 49 340;ID=match$473_49_340;signature_desc=Borrelia lipoprotein;Name=PF00921;status=T;Dbxref="InterPro:IPR000680"
gene-bcCo53	001544	c	lp32	pseudo	beta	Pfam	protein	m	44	273	1.20E-19	+	.	Target=null 44 273;ID=match$2631_44_273;signature_desc=Borrelia lipoprotein;Name=PF00921;status=T;Dbxref="InterPro:IPR000680"
gene-bcCo53	001544	c	lp32			Pfam	protein	m	6	63	3.90E-17	+	.	Target=null 6 63;ID=match$463_6_63;signature_desc=Borrelia lipoprotein;Name=PF00921;status=T;Dbxref="InterPro:IPR000680"
gene-bcCo53	001545	c	lp32	gene	gamma	Pfam	protein	m	33	264	7.90E-51	+	.	Target=null 33 264;ID=match$1421_33_264;signature_desc=Borrelia lipoprotein;Name=PF00921;status=T;Dbxref="InterPro:IPR000680"
gene-bcCo53	001546	c	lp32	pseudo	alpha	Pfam	protein	m	1	50	2.80E-11	+	.	Target=null 1 50;ID=match$1873_1_50;signature_desc=Borrelia lipoprotein;Name=PF00921;status=T;Dbxref="InterPro:IPR000680"
gene-bcCo53	001547	c	lp32	pseudo	gamma	Pfam	protein	m	49	207	2.10E-41	+	.	Target=null 49 207;ID=match$2239_49_207;signature_desc=Borrelia lipoprotein;Name=PF00921;status=T;Dbxref="InterPro:IPR000680"
gene-bcCo53	001547	c	lp32			Pfam	protein	m	48	168	2.90E-37	+	.	Target=null 48 168;ID=match$1992_48_168;signature_desc=Borrelia lipoprotein;Name=PF00921;status=T;Dbxref="InterPro:IPR000680"
gene-bcCo53	001549	c	lp32	pseudo	delta	Pfam	protein	m	44	223	2.40E-43	+	.	Target=null 44 223;ID=match$2530_44_223;signature_desc=Borrelia lipoprotein;Name=PF00921;status=T;Dbxref="InterPro:IPR000680"
gene-bcCo53	001549	c	lp32			Pfam	protein	m	6	100	1.60E-30	+	.	Target=null 6 100;ID=match$3234_6_100;signature_desc=Borrelia lipoprotein;Name=PF00921;status=T;Dbxref="InterPro:IPR000680"
gene-bcCo53	001550	c	lp32	pseudo	beta	Pfam	protein	m	23	146	3.90E-20	+	.	Target=null 23 146;ID=match$647_23_146;signature_desc=Borrelia lipoprotein;Name=PF00921;status=T;Dbxref="InterPro:IPR000680"
gene-bcCo53	001552	c	lp32	pseudo	beta	Pfam	protein	m	10	300	8.60E-47	+	.	Target=null 10 300;ID=match$3133_10_300;signature_desc=Borrelia lipoprotein;Name=PF00921;status=T;Dbxref="InterPro:IPR000680"
gene-bcCo53	001555	c	lp32	pseudo	delta	Pfam	protein	m	32	309	3.00E-82	+	.	Target=null 32 309;ID=match$602_32_309;signature_desc=Borrelia lipoprotein;Name=PF00921;status=T;Dbxref="InterPro:IPR000680"
gene-bcCo53	001557	c	lp32	pseudo	gamma	Pfam	protein	m	1	241	6.60E-60	+	.	Target=null 1 241;ID=match$307_1_241;signature_desc=Borrelia lipoprotein;Name=PF00921;status=T;Dbxref="InterPro:IPR000680"
gene-bcCo53	001557	c	lp32			Pfam	protein	m	17	54	3.70E-13	+	.	Target=null 17 54;ID=match$1440_17_54;signature_desc=Borrelia lipoprotein;Name=PF00921;status=T;Dbxref="InterPro:IPR000680"
gene-bcCo53	001558	c	lp32	gene	beta	Pfam	protein	m	50	198	1.50E-19	+	.	Target=null 50 198;ID=match$1551_50_198;signature_desc=Borrelia lipoprotein;Name=PF00921;status=T;Dbxref="InterPro:IPR000680"
gene-bcCo53	001559	c	lp32	gene	beta	Pfam	protein	m	6	113	2.80E-10	+	.	Target=null 6 113;ID=match$3010_6_113;signature_desc=Borrelia lipoprotein;Name=PF00921;status=T;Dbxref="InterPro:IPR000680"
gene-bcCo53	001562	c	lp32	gene	beta	Pfam	protein	m	64	329	3.90E-23	+	.	Target=null 64 329;ID=match$108_64_329;signature_desc=Borrelia lipoprotein;Name=PF00921;status=T;Dbxref="InterPro:IPR000680"
gene-bcCo53	001589	c	lp33	gene	gamma	Pfam	protein	m	45	337	3.10E-88	+	.	Target=null 45 337;ID=match$469_45_337;signature_desc=Borrelia lipoprotein;Name=PF00921;status=T;Dbxref="InterPro:IPR000680"
gene-bcCo53	001597	c	lp34	gene	gamma	Pfam	protein	m	49	337	2.30E-67	+	.	Target=null 49 337;ID=match$2224_49_337;signature_desc=Borrelia lipoprotein;Name=PF00921;status=T;Dbxref="InterPro:IPR000680"
gene-bcCo53	001598	c	lp34	gene	gamma	Pfam	protein	m	49	351	1.30E-70	+	.	Target=null 49 351;ID=match$1226_49_351;signature_desc=Borrelia lipoprotein;Name=PF00921;status=T;Dbxref="InterPro:IPR000680"
gene-bcCo53	001599	c	lp34	gene	alpha	Pfam	protein	m	53	336	1.10E-43	+	.	Target=null 53 336;ID=match$1243_53_336;signature_desc=Borrelia lipoprotein;Name=PF00921;status=T;Dbxref="InterPro:IPR000680"
gene-bcCo53	001600	c	lp34	gene	gamma	Pfam	protein	m	49	329	3.10E-80	+	.	Target=null 49 329;ID=match$1271_49_329;signature_desc=Borrelia lipoprotein;Name=PF00921;status=T;Dbxref="InterPro:IPR000680"
gene-bcCo53	001601	c	lp34	gene	delta	Pfam	protein	m	50	334	4.30E-64	+	.	Target=null 50 334;ID=match$1751_50_334;signature_desc=Borrelia lipoprotein;Name=PF00921;status=T;Dbxref="InterPro:IPR000680"
gene-bcCo53	001602	c	lp34	pseudo	alpha	Pfam	protein	m	54	109	9.80E-07	+	.	Target=null 54 109;ID=match$1319_54_109;signature_desc=Borrelia lipoprotein;Name=PF00921;status=T;Dbxref="InterPro:IPR000680"
gene-bcCo53	001602	c	lp34			Pfam	protein	m	24	233	2.40E-28	+	.	Target=null 24 233;ID=match$1949_24_233;signature_desc=Borrelia lipoprotein;Name=PF00921;status=T;Dbxref="InterPro:IPR000680"
gene-bcCo53	001604	c	lp34	gene	gamma	Pfam	protein	m	49	329	2.30E-79	+	.	Target=null 49 329;ID=match$1127_49_329;signature_desc=Borrelia lipoprotein;Name=PF00921;status=T;Dbxref="InterPro:IPR000680"
gene-bcCo53	001605	c	lp34	gene	gamma	Pfam	protein	m	49	338	3.80E-83	+	.	Target=null 49 338;ID=match$2719_49_338;signature_desc=Borrelia lipoprotein;Name=PF00921;status=T;Dbxref="InterPro:IPR000680"
gene-bcCo53	001606	c	lp34	gene	gamma	Pfam	protein	m	49	344	2.20E-85	+	.	Target=null 49 344;ID=match$223_49_344;signature_desc=Borrelia lipoprotein;Name=PF00921;status=T;Dbxref="InterPro:IPR000680"
gene-bcCo53	001607	c	lp34	gene	gamma	Pfam	protein	m	49	341	4.90E-83	+	.	Target=null 49 341;ID=match$3276_49_341;signature_desc=Borrelia lipoprotein;Name=PF00921;status=T;Dbxref="InterPro:IPR000680"
gene-bcCo53	001608	c	lp34	gene	gamma	Pfam	protein	m	49	335	1.10E-81	+	.	Target=null 49 335;ID=match$2712_49_335;signature_desc=Borrelia lipoprotein;Name=PF00921;status=T;Dbxref="InterPro:IPR000680"
gene-bcCo53	001609	c	lp34	gene	beta	Pfam	protein	m	50	337	7.00E-45	+	.	Target=null 50 337;ID=match$1247_50_337;signature_desc=Borrelia lipoprotein;Name=PF00921;status=T;Dbxref="InterPro:IPR000680"
gene-bcCo53	001610	c	lp34	gene	gamma	Pfam	protein	m	1	56	3.20E-16	+	.	Target=null 1 56;ID=match$2876_1_56;signature_desc=Borrelia lipoprotein;Name=PF00921;status=T;Dbxref="InterPro:IPR000680"
gene-bcCo53	001611	c	lp34	gene	delta	Pfam	protein	m	50	335	1.90E-60	+	.	Target=null 50 335;ID=match$2155_50_335;signature_desc=Borrelia lipoprotein;Name=PF00921;status=T;Dbxref="InterPro:IPR000680"
gene-bcCo53	001612	c	lp34	gene	beta	Pfam	protein	m	51	339	1.40E-40	+	.	Target=null 51 339;ID=match$2755_51_339;signature_desc=Borrelia lipoprotein;Name=PF00921;status=T;Dbxref="InterPro:IPR000680"
gene-bcCo53	001626	c	lp38	pseudo	beta	Pfam	protein	m	33	173	6.60E-19	+	.	Target=null 33 173;ID=match$427_33_173;signature_desc=Borrelia lipoprotein;Name=PF00921;status=T;Dbxref="InterPro:IPR000680"
gene-bcCo53	001626	c	lp38			Pfam	protein	m	18	139	2.00E-23	+	.	Target=null 18 139;ID=match$3145_18_139;signature_desc=Borrelia lipoprotein;Name=PF00921;status=T;Dbxref="InterPro:IPR000680"
gene-bcCo53	001627	c	lp38	gene	delta	Pfam	protein	m	44	232	9.30E-41	+	.	Target=null 44 232;ID=match$146_44_232;signature_desc=Borrelia lipoprotein;Name=PF00921;status=T;Dbxref="InterPro:IPR000680"
gene-bcCo53	001629	c	lp38	gene	delta	Pfam	protein	m	49	326	4.00E-77	+	.	Target=null 49 326;ID=match$1169_49_326;signature_desc=Borrelia lipoprotein;Name=PF00921;status=T;Dbxref="InterPro:IPR000680"
gene-bcCo53	001630	c	lp38	gene	delta	Pfam	protein	m	49	329	1.20E-73	+	.	Target=null 49 329;ID=match$2916_49_329;signature_desc=Borrelia lipoprotein;Name=PF00921;status=T;Dbxref="InterPro:IPR000680"
gene-bcCo53	001631	c	lp38	pseudo	alpha	Pfam	protein	m	50	80	4.70E-06	+	.	Target=null 50 80;ID=match$1566_50_80;signature_desc=Borrelia lipoprotein;Name=PF00921;status=T;Dbxref="InterPro:IPR000680"
gene-bcCo53	001631	c	lp38			Pfam	protein	m	1	193	2.20E-22	+	.	Target=null 1 193;ID=match$1924_1_193;signature_desc=Borrelia lipoprotein;Name=PF00921;status=T;Dbxref="InterPro:IPR000680"
gene-bcCo53	001631	c	lp38			Pfam	protein	m	8	76	3.30E-19	+	.	Target=null 8 76;ID=match$1964_8_76;signature_desc=Borrelia lipoprotein;Name=PF00921;status=T;Dbxref="InterPro:IPR000680"
gene-bcCo53	001632	c	lp38	gene	gamma	Pfam	protein	m	49	329	3.80E-84	+	.	Target=null 49 329;ID=match$878_49_329;signature_desc=Borrelia lipoprotein;Name=PF00921;status=T;Dbxref="InterPro:IPR000680"
gene-bcCo53	001633	c	lp38	gene	alpha	Pfam	protein	m	50	346	1.70E-58	+	.	Target=null 50 346;ID=match$675_50_346;signature_desc=Borrelia lipoprotein;Name=PF00921;status=T;Dbxref="InterPro:IPR000680"
gene-bcCo53	001634	c	lp38	gene	delta	Pfam	protein	m	49	291	1.70E-45	+	.	Target=null 49 291;ID=match$1825_49_291;signature_desc=Borrelia lipoprotein;Name=PF00921;status=T;Dbxref="InterPro:IPR000680"
gene-bcCo53	001635	c	lp38	gene	delta	Pfam	protein	m	1	35	8.30E-08	+	.	Target=null 1 35;ID=match$157_1_35;signature_desc=Borrelia lipoprotein;Name=PF00921;status=T;Dbxref="InterPro:IPR000680"
gene-bcCo53	001636	c	lp38	gene	beta	Pfam	protein	m	50	338	7.40E-45	+	.	Target=null 50 338;ID=match$2096_50_338;signature_desc=Borrelia lipoprotein;Name=PF00921;status=T;Dbxref="InterPro:IPR000680"
gene-bcCo53	001637	c	lp38	gene	gamma	Pfam	protein	m	49	342	9.50E-80	+	.	Target=null 49 342;ID=match$1883_49_342;signature_desc=Borrelia lipoprotein;Name=PF00921;status=T;Dbxref="InterPro:IPR000680"
gene-bcCo53	001638	c	lp38	gene	gamma	Pfam	protein	m	49	333	2.20E-83	+	.	Target=null 49 333;ID=match$770_49_333;signature_desc=Borrelia lipoprotein;Name=PF00921;status=T;Dbxref="InterPro:IPR000680"
gene-bcCo53	001640	c	lp38	pseudo	delta	Pfam	protein	m	5	281	5.40E-77	+	.	Target=null 5 281;ID=match$978_5_281;signature_desc=Borrelia lipoprotein;Name=PF00921;status=T;Dbxref="InterPro:IPR000680"
gene-bcCo53	001641	c	lp38	pseudo	gamma	Pfam	protein	m	21	219	2.90E-46	+	.	Target=null 21 219;ID=match$1367_21_219;signature_desc=Borrelia lipoprotein;Name=PF00921;status=T;Dbxref="InterPro:IPR000680"
gene-bcCo53	001641	c	lp38			Pfam	protein	m	3	89	5.00E-25	+	.	Target=null 3 89;ID=match$2466_3_89;signature_desc=Borrelia lipoprotein;Name=PF00921;status=T;Dbxref="InterPro:IPR000680"
gene-bcCo53	001643	c	lp38	gene	gamma	Pfam	protein	m	53	290	6.90E-50	+	.	Target=null 53 290;ID=match$2750_53_290;signature_desc=Borrelia lipoprotein;Name=PF00921;status=T;Dbxref="InterPro:IPR000680"
gene-bcCo53	001645	c	lp38	gene	alpha	Pfam	protein	m	50	346	4.40E-62	+	.	Target=null 50 346;ID=match$2105_50_346;signature_desc=Borrelia lipoprotein;Name=PF00921;status=T;Dbxref="InterPro:IPR000680"
gene-bcCo53	001646	c	lp38	gene	beta	Pfam	protein	m	50	333	9.30E-41	+	.	Target=null 50 333;ID=match$1681_50_333;signature_desc=Borrelia lipoprotein;Name=PF00921;status=T;Dbxref="InterPro:IPR000680"
gene-bcCo53	001647	c	lp38	pseudo	gamma	Pfam	protein	m	52	221	2.40E-35	+	.	Target=null 52 221;ID=match$2192_52_221;signature_desc=Borrelia lipoprotein;Name=PF00921;status=T;Dbxref="InterPro:IPR000680"
gene-bcCo53	001647	c	lp38			Pfam	protein	m	1	65	9.50E-16	+	.	Target=null 1 65;ID=match$2378_1_65;signature_desc=Borrelia lipoprotein;Name=PF00921;status=T;Dbxref="InterPro:IPR000680"
gene-bcCo53	001647	c	lp38			Pfam	protein	m	25	63	7.80E-11	+	.	Target=null 25 63;ID=match$476_25_63;signature_desc=Borrelia lipoprotein;Name=PF00921;status=T;Dbxref="InterPro:IPR000680"
gene-bcCo53	001691	c	lp39	gene	delta	Pfam	protein	m	49	329	5.20E-78	+	.	Target=null 49 329;ID=match$3054_49_329;signature_desc=Borrelia lipoprotein;Name=PF00921;status=T;Dbxref="InterPro:IPR000680"
gene-bcCo53	001702	c	lp60	gene	gamma	Pfam	protein	m	49	337	2.30E-67	+	.	Target=null 49 337;ID=match$2226_49_337;signature_desc=Borrelia lipoprotein;Name=PF00921;status=T;Dbxref="InterPro:IPR000680"
gene-bcCo53	001703	c	lp60	gene	gamma	Pfam	protein	m	49	351	1.30E-70	+	.	Target=null 49 351;ID=match$1224_49_351;signature_desc=Borrelia lipoprotein;Name=PF00921;status=T;Dbxref="InterPro:IPR000680"
gene-bcCo53	001704	c	lp60	gene	delta	Pfam	protein	m	49	336	6.30E-61	+	.	Target=null 49 336;ID=match$1301_49_336;signature_desc=Borrelia lipoprotein;Name=PF00921;status=T;Dbxref="InterPro:IPR000680"
gene-bcCo53	001705	c	lp60	gene	delta	Pfam	protein	m	49	327	1.10E-82	+	.	Target=null 49 327;ID=match$296_49_327;signature_desc=Borrelia lipoprotein;Name=PF00921;status=T;Dbxref="InterPro:IPR000680"
gene-bcCo53	001706	c	lp60	gene	delta	Pfam	protein	m	49	337	5.70E-58	+	.	Target=null 49 337;ID=match$2458_49_337;signature_desc=Borrelia lipoprotein;Name=PF00921;status=T;Dbxref="InterPro:IPR000680"
gene-bcCo53	001707	c	lp60	pseudo	gamma	Pfam	protein	m	7	45	3.70E-13	+	.	Target=null 7 45;ID=match$3203_7_45;signature_desc=Borrelia lipoprotein;Name=PF00921;status=T;Dbxref="InterPro:IPR000680"
gene-bcCo53	001708	c	lp60	gene	gamma	Pfam	protein	m	44	335	9.10E-82	+	.	Target=null 44 335;ID=match$2653_44_335;signature_desc=Borrelia lipoprotein;Name=PF00921;status=T;Dbxref="InterPro:IPR000680"
gene-bcCo53	001709	c	lp60	gene	beta	Pfam	protein	m	50	333	6.70E-47	+	.	Target=null 50 333;ID=match$1374_50_333;signature_desc=Borrelia lipoprotein;Name=PF00921;status=T;Dbxref="InterPro:IPR000680"
gene-bcCo53	001710	c	lp60	gene	gamma	Pfam	protein	m	52	339	6.40E-82	+	.	Target=null 52 339;ID=match$3156_52_339;signature_desc=Borrelia lipoprotein;Name=PF00921;status=T;Dbxref="InterPro:IPR000680"
gene-bcCo53	001711	c	lp60	pseudo	beta	Pfam	protein	m	5	276	6.90E-40	+	.	Target=null 5 276;ID=match$3128_5_276;signature_desc=Borrelia lipoprotein;Name=PF00921;status=T;Dbxref="InterPro:IPR000680"
gene-bcCo53	001712	c	lp60	pseudo	delta	Pfam	protein	m	6	169	2.20E-40	+	.	Target=null 6 169;ID=match$1354_6_169;signature_desc=Borrelia lipoprotein;Name=PF00921;status=T;Dbxref="InterPro:IPR000680"
gene-bcCo53	001712	c	lp60			Pfam	protein	m	16	75	7.00E-16	+	.	Target=null 16 75;ID=match$1627_16_75;signature_desc=Borrelia lipoprotein;Name=PF00921;status=T;Dbxref="InterPro:IPR000680"
gene-bcCo53	001712	c	lp60			Pfam	protein	m	20	55	1.20E-10	+	.	Target=null 20 55;ID=match$1597_20_55;signature_desc=Borrelia lipoprotein;Name=PF00921;status=T;Dbxref="InterPro:IPR000680"
gene-bcCo53	001715	c	lp60	gene	gamma	Pfam	protein	m	50	331	5.50E-81	+	.	Target=null 50 331;ID=match$1584_50_331;signature_desc=Borrelia lipoprotein;Name=PF00921;status=T;Dbxref="InterPro:IPR000680"
gene-bcCo53	001724	c	lp60	gene	beta	Pfam	protein	m	95	353	4.80E-23	+	.	Target=null 95 353;ID=match$2981_95_353;signature_desc=Borrelia lipoprotein;Name=PF00921;status=T;Dbxref="InterPro:IPR000680"
gene-bcCo53	001733	c	lp60	gene	alpha	Pfam	protein	m	50	343	1.50E-51	+	.	Target=null 50 343;ID=match$653_50_343;signature_desc=Borrelia lipoprotein;Name=PF00921;status=T;Dbxref="InterPro:IPR000680"
gene-bcCo53	001734	c	lp60	gene	alpha	Pfam	protein	m	56	350	3.40E-46	+	.	Target=null 56 350;ID=match$1059_56_350;signature_desc=Borrelia lipoprotein;Name=PF00921;status=T;Dbxref="InterPro:IPR000680"
gene-bcCo53	001735	c	lp60	gene	gamma	Pfam	protein	m	49	337	1.00E-83	+	.	Target=null 49 337;ID=match$1813_49_337;signature_desc=Borrelia lipoprotein;Name=PF00921;status=T;Dbxref="InterPro:IPR000680"
gene-bcCo53	001736	c	lp60	gene	beta	Pfam	protein	m	50	335	2.60E-47	+	.	Target=null 50 335;ID=match$2518_50_335;signature_desc=Borrelia lipoprotein;Name=PF00921;status=T;Dbxref="InterPro:IPR000680"
gene-bcCo53	001737	c	lp60	gene	delta	Pfam	protein	m	49	327	1.10E-77	+	.	Target=null 49 327;ID=match$774_49_327;signature_desc=Borrelia lipoprotein;Name=PF00921;status=T;Dbxref="InterPro:IPR000680"
gene-bcCo53	001738	c	lp60	pseudo	beta	Pfam	protein	m	12	51	1.20E-07	+	.	Target=null 12 51;ID=match$143_12_51;signature_desc=Borrelia lipoprotein;Name=PF00921;status=T;Dbxref="InterPro:IPR000680"
gene-bcCo53	001739	c	lp60	gene	alpha	Pfam	protein	m	50	348	2.20E-66	+	.	Target=null 50 348;ID=match$2823_50_348;signature_desc=Borrelia lipoprotein;Name=PF00921;status=T;Dbxref="InterPro:IPR000680"
gene-bcCo53	001740	c	lp60	pseudo	gamma	Pfam	protein	m	49	261	1.30E-50	+	.	Target=null 49 261;ID=match$3231_49_261;signature_desc=Borrelia lipoprotein;Name=PF00921;status=T;Dbxref="InterPro:IPR000680"
gene-bcCo53	001740	c	lp60			Pfam	protein	m	5	70	1.10E-22	+	.	Target=null 5 70;ID=match$2541_5_70;signature_desc=Borrelia lipoprotein;Name=PF00921;status=T;Dbxref="InterPro:IPR000680"
gene-bcCo53	001741	c	lp60	gene	delta	Pfam	protein	m	49	338	1.30E-71	+	.	Target=null 49 338;ID=match$2188_49_338;signature_desc=Borrelia lipoprotein;Name=PF00921;status=T;Dbxref="InterPro:IPR000680"
gene-bcCo53	001742	c	lp60	pseudo	beta	Pfam	protein	m	50	330	4.80E-43	+	.	Target=null 50 330;ID=match$1954_50_330;signature_desc=Borrelia lipoprotein;Name=PF00921;status=T;Dbxref="InterPro:IPR000680"
gene-bcCo53	001743	c	lp60	gene	beta	Pfam	protein	m	50	341	1.40E-47	+	.	Target=null 50 341;ID=match$3047_50_341;signature_desc=Borrelia lipoprotein;Name=PF00921;status=T;Dbxref="InterPro:IPR000680"
gene-bcCo53	001744	c	lp60	gene	beta	Pfam	protein	m	50	332	7.40E-41	+	.	Target=null 50 332;ID=match$3002_50_332;signature_desc=Borrelia lipoprotein;Name=PF00921;status=T;Dbxref="InterPro:IPR000680"
gene-bcCo53	001745	c	lp60	gene	gamma	Pfam	protein	m	49	330	1.50E-83	+	.	Target=null 49 330;ID=match$2342_49_330;signature_desc=Borrelia lipoprotein;Name=PF00921;status=T;Dbxref="InterPro:IPR000680"
gene-bcCo53	001746	c	lp60	pseudo	delta	Pfam	protein	m	18	101	2.20E-24	+	.	Target=null 18 101;ID=match$442_18_101;signature_desc=Borrelia lipoprotein;Name=PF00921;status=T;Dbxref="InterPro:IPR000680"
gene-bcCo53	001746	c	lp60			Pfam	protein	m	17	209	1.30E-53	+	.	Target=null 17 209;ID=match$1959_17_209;signature_desc=Borrelia lipoprotein;Name=PF00921;status=T;Dbxref="InterPro:IPR000680"

Vsp	replicon	gene/pseudo									
gene-bpuSUM 001588	lp25	gene		Pfam	protein matc	39	204	1E-51	+	.	Target=null 39 204;ID=match$1794_39_204;signature_desc=Lipoprotein;Name=PF01441;status=T;Dbxref="InterPro:IPR001800"
gene-bpuSUM 001664	lp35	gene		Pfam	protein matc	40	209	1.9E-49	+	.	Target=null 40 209;ID=match$2768_40_209;signature_desc=Lipoprotein;Name=PF01441;status=T;Dbxref="InterPro:IPR001800"
gene-bpuSUM 001668	lp35	gene		Pfam	protein matc	39	213	6.6E-52	+	.	Target=null 39 213;ID=match$333_39_213;signature_desc=Lipoprotein;Name=PF01441;status=T;Dbxref="InterPro:IPR001800"
gene-bpuSUM 001683	lp35	gene		Pfam	protein matc	39	206	9.3E-50	+	.	Target=null 39 206;ID=match$1302_39_206;signature_desc=Lipoprotein;Name=PF01441;status=T;Dbxref="InterPro:IPR001800"
gene-bpuSUM 001706	lp39	gene		Pfam	protein matc	39	208	2.7E-55	+	.	Target=null 39 208;ID=match$3336_39_208;signature_desc=Lipoprotein;Name=PF01441;status=T;Dbxref="InterPro:IPR001800"
gene-bpuSUM 001711	lp39	gene		Pfam	protein matc	39	206	7.2E-40	+	.	Target=null 39 206;ID=match$3437_39_206;signature_desc=Lipoprotein;Name=PF01441;status=T;Dbxref="InterPro:IPR001800"
gene-bpuSUM 001713	lp39	pseudo		Pfam	protein matc	28	79	1.1E-18	+	.	Target=null 28 79;ID=match$2180_28_79;signature_desc=Lipoprotein;Name=PF01441;status=T;Dbxref="InterPro:IPR001800"
gene-bpuSUM 001713	lp39	pseudo		Pfam	protein matc	1	119	1.1E-27	+	.	Target=null 1 119;ID=match$2197_1_119;signature_desc=Lipoprotein;Name=PF01441;status=T;Dbxref="InterPro:IPR001800"
gene-bpuSUM 001717	lp39	gene		Pfam	protein matc	39	207	2.5E-60	+	.	Target=null 39 207;ID=match$2090_39_207;signature_desc=Lipoprotein;Name=PF01441;status=T;Dbxref="InterPro:IPR001800"
gene-bpuSUM 001722	lp39	gene		Pfam	protein matc	39	211	6.7E-47	+	.	Target=null 39 211;ID=match$6_39_211;signature_desc=Lipoprotein;Name=PF01441;status=T;Dbxref="InterPro:IPR001800"
gene-bpuSUM 001750	lp41	gene		Pfam	protein matc	39	201	7.5E-49	+	.	Target=null 39 201;ID=match$110_39_201;signature_desc=Lipoprotein;Name=PF01441;status=T;Dbxref="InterPro:IPR001800"
gene-bpuSUM 001764	lp41	gene		Pfam	protein matc	40	209	1.9E-49	+	.	Target=null 40 209;ID=match$2770_40_209;signature_desc=Lipoprotein;Name=PF01441;status=T;Dbxref="InterPro:IPR001800"
gene-bpuSUM 001778	lp46	gene		Pfam	protein matc	39	207	1.6E-58	+	.	Target=null 39 207;ID=match$2332_39_207;signature_desc=Lipoprotein;Name=PF01441;status=T;Dbxref="InterPro:IPR001800"
gene-bpuSUM 001781	lp46	pseudo		Pfam	protein matc	39	145	3.2E-31	+	.	Target=null 39 145;ID=match$1777_39_145;signature_desc=Lipoprotein;Name=PF01441;status=T;Dbxref="InterPro:IPR001800"
gene-bpuSUM 001781	lp46	pseudo		Pfam	protein matc	11	74	2.7E-16	+	.	Target=null 11 74;ID=match$2312_11_74;signature_desc=Lipoprotein;Name=PF01441;status=T;Dbxref="InterPro:IPR001800"
gene-bpuSUM 001784	lp46	pseudo		Pfam	protein matc	39	147	1.8E-36	+	.	Target=null 39 147;ID=match$2721_39_147;signature_desc=Lipoprotein;Name=PF01441;status=T;Dbxref="InterPro:IPR001800"
gene-bpuSUM 001784	lp46	pseudo		Pfam	protein matc	2	61	3.7E-13	+	.	Target=null 2 61;ID=match$3611_2_61;signature_desc=Lipoprotein;Name=PF01441;status=T;Dbxref="InterPro:IPR001800"
gene-bpuSUM 001787	lp46	gene		Pfam	protein matc	39	202	6E-42	+	.	Target=null 39 202;ID=match$1316_39_202;signature_desc=Lipoprotein;Name=PF01441;status=T;Dbxref="InterPro:IPR001800"
gene-bpuSUM 001795	lp46	gene		Pfam	protein matc	38	205	6E-65	+	.	Target=null 38 205;ID=match$3743_38_205;signature_desc=Lipoprotein;Name=PF01441;status=T;Dbxref="InterPro:IPR001800"
gene-bpuSUM 001801	lp46	gene		Pfam	protein matc	39	206	3.4E-65	+	.	Target=null 39 206;ID=match$2638_39_206;signature_desc=Lipoprotein;Name=PF01441;status=T;Dbxref="InterPro:IPR001800"
gene-bpuSUM 001840	lp47	gene		Pfam	protein matc	39	202	6E-42	+	.	Target=null 39 202;ID=match$1314_39_202;signature_desc=Lipoprotein;Name=PF01441;status=T;Dbxref="InterPro:IPR001800"
gene-bpuSUM 001843	lp47	pseudo		Pfam	protein matc	39	147	1.8E-36	+	.	Target=null 39 147;ID=match$2719_39_147;signature_desc=Lipoprotein;Name=PF01441;status=T;Dbxref="InterPro:IPR001800"
gene-bpuSUM 001843	lp47	pseudo		Pfam	protein matc	2	61	3.7E-13	+	.	Target=null 2 61;ID=match$3607_2_61;signature_desc=Lipoprotein;Name=PF01441;status=T;Dbxref="InterPro:IPR001800"
gene-bpuSUM 001846	lp47	pseudo		Pfam	protein matc	39	145	3.2E-31	+	.	Target=null 39 145;ID=match$1775_39_145;signature_desc=Lipoprotein;Name=PF01441;status=T;Dbxref="InterPro:IPR001800"
gene-bpuSUM 001846	lp47	pseudo		Pfam	protein matc	11	74	2.7E-16	+	.	Target=null 11 74;ID=match$2314_11_74;signature_desc=Lipoprotein;Name=PF01441;status=T;Dbxref="InterPro:IPR001800"
gene-bpuSUM 001849	lp47	gene		Pfam	protein matc	39	207	1.6E-58	+	.	Target=null 39 207;ID=match$2330_39_207;signature_desc=Lipoprotein;Name=PF01441;status=T;Dbxref="InterPro:IPR001800"
gene-bpuSUM 001862	lp57	gene		Pfam	protein matc	39	207	1.6E-58	+	.	Target=null 39 207;ID=match$2334_39_207;signature_desc=Lipoprotein;Name=PF01441;status=T;Dbxref="InterPro:IPR001800"
gene-bpuSUM 001864	lp57	gene		Pfam	protein matc	39	207	2.4E-65	+	.	Target=null 39 207;ID=match$83_39_207;signature_desc=Lipoprotein;Name=PF01441;status=T;Dbxref="InterPro:IPR001800"
gene-bpuSUM 001873	lp57	gene		Pfam	protein matc	39	211	6.1E-49	+	.	Target=null 39 211;ID=match$3568_39_211;signature_desc=Lipoprotein;Name=PF01441;status=T;Dbxref="InterPro:IPR001800"
gene-bpuSUM 001894	lp57	gene		Pfam	protein matc	39	206	2.3E-54	+	.	Target=null 39 206;ID=match$1408_39_206;signature_desc=Lipoprotein;Name=PF01441;status=T;Dbxref="InterPro:IPR001800"
gene-bpuSUM 001896	lp57	gene		Pfam	protein matc	28	196	2.7E-55	+	.	Target=null 28 196;ID=match$3575_28_196;signature_desc=Lipoprotein;Name=PF01441;status=T;Dbxref="InterPro:IPR001800"
gene-bpuSUM 001899	lp57	gene		Pfam	protein matc	38	206	1.6E-63	+	.	Target=null 38 206;ID=match$214_38_206;signature_desc=Lipoprotein;Name=PF01441;status=T;Dbxref="InterPro:IPR001800"
gene-bpuSUM 001905	lp57	gene		Pfam	protein matc	39	211	6.7E-47	+	.	Target=null 39 211;ID=match$4_39_211;signature_desc=Lipoprotein;Name=PF01441;status=T;Dbxref="InterPro:IPR001800"
gene-bpuSUM 001918	lp65	gene		Pfam	protein matc	28	196	3.8E-62	+	.	Target=null 28 196;ID=match$2605_28_196;signature_desc=Lipoprotein;Name=PF01441;status=T;Dbxref="InterPro:IPR001800"
gene-bpuSUM 001930	lp65	gene		Pfam	protein matc	39	206	8.7E-61	+	.	Target=null 39 206;ID=match$1157_39_206;signature_desc=Lipoprotein;Name=PF01441;status=T;Dbxref="InterPro:IPR001800"
gene-bpuSUM 001935	lp65	gene		Pfam	protein matc	40	206	1.9E-63	+	.	Target=null 40 206;ID=match$2241_40_206;signature_desc=Lipoprotein;Name=PF01441;status=T;Dbxref="InterPro:IPR001800"
gene-bpuSUM 001937	lp65	gene		Pfam	protein matc	39	206	1.2E-56	+	.	Target=null 39 206;ID=match$923_39_206;signature_desc=Lipoprotein;Name=PF01441;status=T;Dbxref="InterPro:IPR001800"
gene-bpuSUM 001952	lp65	gene		Pfam	protein matc	40	208	4.1E-63	+	.	Target=null 40 208;ID=match$3700_40_208;signature_desc=Lipoprotein;Name=PF01441;status=T;Dbxref="InterPro:IPR001800"
gene-bpuSUM 001956	lp65	gene		Pfam	protein matc	38	205	6E-65	+	.	Target=null 38 205;ID=match$3745_38_205;signature_desc=Lipoprotein;Name=PF01441;status=T;Dbxref="InterPro:IPR001800"
gene-bpuSUM 001964	lp65	gene		Pfam	protein matc	39	202	6E-42	+	.	Target=null 39 202;ID=match$1318_39_202;signature_desc=Lipoprotein;Name=PF01441;status=T;Dbxref="InterPro:IPR001800"
gene-bpuSUM 001967	lp65	pseudo		Pfam	protein matc	39	147	1.8E-36	+	.	Target=null 39 147;ID=match$2723_39_147;signature_desc=Lipoprotein;Name=PF01441;status=T;Dbxref="InterPro:IPR001800"
gene-bpuSUM 001967	lp65	pseudo		Pfam	protein matc	2	61	3.7E-13	+	.	Target=null 2 61;ID=match$3609_2_61;signature_desc=Lipoprotein;Name=PF01441;status=T;Dbxref="InterPro:IPR001800"
gene-bpuSUM 001970	lp65	gene		Pfam	protein matc	40	208	1.1E-49	+	.	Target=null 40 208;ID=match$1149_40_208;signature_desc=Lipoprotein;Name=PF01441;status=T;Dbxref="InterPro:IPR001800"
gene-bpuSUM 001973	lp65	gene		Pfam	protein matc	39	207	1.6E-58	+	.	Target=null 39 207;ID=match$2336_39_207;signature_desc=Lipoprotein;Name=PF01441;status=T;Dbxref="InterPro:IPR001800"
Vlp			Subfamily								
gene-bpuSUM 001573	lp25	pseudo	beta	Pfam	protein matc	2	116	3.6E-11	+	.	Target=null 2 116;ID=match$422_2_116;signature_desc=Borrelia lipoprotein;Name=PF00921;status=T;Dbxref="InterPro:IPR000680"
gene-bpuSUM 001573	lp25	pseudo		Pfam	protein matc	109	159	9.8E-11	+	.	Target=null 109 159;ID=match$3635_109_159;signature_desc=Borrelia lipoprotein;Name=PF00921;status=T;Dbxref="InterPro:IPR000680"
gene-bpuSUM 001573	lp25	pseudo		Pfam	protein matc	33	110	0.000019	+	.	Target=null 33 110;ID=match$3635_33_110;signature_desc=Borrelia lipoprotein;Name=PF00921;status=T;Dbxref="InterPro:IPR000680"
gene-bpuSUM 001574	lp25	gene	alpha	Pfam	protein matc	58	347	1.4E-68	+	.	Target=null 58 347;ID=match$2290_58_347;signature_desc=Borrelia lipoprotein;Name=PF00921;status=T;Dbxref="InterPro:IPR000680"
gene-bpuSUM 001586	lp25	gene	gamma	Pfam	protein matc	1	270	2.6E-81	+	.	Target=null 1 270;ID=match$2519_1_270;signature_desc=Borrelia lipoprotein;Name=PF00921;status=T;Dbxref="InterPro:IPR000680"
gene-bpuSUM 001589	lp25	gene	delta	Pfam	protein matc	19	298	2.2E-85	+	.	Target=null 19 298;ID=match$660_19_298;signature_desc=Borrelia lipoprotein;Name=PF00921;status=T;Dbxref="InterPro:IPR000680"
gene-bpuSUM 001590	lp25	gene	gamma	Pfam	protein matc	213	269	2.7E-18	+	.	Target=null 213 269;ID=match$1873_213_269;signature_desc=Borrelia lipoprotein;Name=PF00921;status=T;Dbxref="InterPro:IPR000680"
gene-bpuSUM 001590	lp25	gene		Pfam	protein matc	34	215	5.2E-44	+	.	Target=null 34 215;ID=match$1873_34_215;signature_desc=Borrelia lipoprotein;Name=PF00921;status=T;Dbxref="InterPro:IPR000680"
gene-bpuSUM 001661	lp35	pseudo	gamma	Pfam	protein matc	34	82	2.6E-16	+	.	Target=null 34 82;ID=match$3347_34_82;signature_desc=Borrelia lipoprotein;Name=PF00921;status=T;Dbxref="InterPro:IPR000680"
gene-bpuSUM 001661	lp35	pseudo		Pfam	protein matc	3	46	0.00000052	+	.	Target=null 3 46;ID=match$542_3_46;signature_desc=Borrelia lipoprotein;Name=PF00921;status=T;Dbxref="InterPro:IPR000680"
gene-bpuSUM 001661	lp35	pseudo		Pfam	protein matc	19	197	4.4E-45	+	.	Target=null 19 197;ID=match$882_19_197;signature_desc=Borrelia lipoprotein;Name=PF00921;status=T;Dbxref="InterPro:IPR000680"
gene-bpuSUM 001662	lp35	pseudo	delta	Pfam	protein matc	14	288	2.7E-83	+	.	Target=null 14 288;ID=match$1031_14_288;signature_desc=Borrelia lipoprotein;Name=PF00921;status=T;Dbxref="InterPro:IPR000680"
gene-bpuSUM 001663	lp35	pseudo	alpha	Pfam	protein matc	56	305	6E-55	+	.	Target=null 56 305;ID=match$2669_56_305;signature_desc=Borrelia lipoprotein;Name=PF00921;status=T;Dbxref="InterPro:IPR000680"
gene-bpuSUM 001663	lp35	pseudo		Pfam	protein matc	2	34	0.00000061	+	.	Target=null 2 34;ID=match$1458_2_34;signature_desc=Borrelia lipoprotein;Name=PF00921;status=T;Dbxref="InterPro:IPR000680"
gene-bpuSUM 001665	lp35	gene	gamma	Pfam	protein matc	34	307	2.6E-83	+	.	Target=null 34 307;ID=match$1755_34_307;signature_desc=Borrelia lipoprotein;Name=PF00921;status=T;Dbxref="InterPro:IPR000680"
gene-bpuSUM 001667	lp35	pseudo	beta	Pfam	protein matc	19	60	0.000065	+	.	Target=null 19 60;ID=match$1282_19_60;signature_desc=Borrelia lipoprotein;Name=PF00921;status=T;Dbxref="InterPro:IPR000680"
gene-bpuSUM 001667	lp35	pseudo		Pfam	protein matc	27	155	3.1E-25	+	.	Target=null 27 155;ID=match$2921_27_155;signature_desc=Borrelia lipoprotein;Name=PF00921;status=T;Dbxref="InterPro:IPR000680"
gene-bpuSUM 001669	lp35	pseudo	delta	Pfam	protein matc	44	120	4.5E-21	+	.	Target=null 44 120;ID=match$2034_44_120;signature_desc=Borrelia lipoprotein;Name=PF00921;status=T;Dbxref="InterPro:IPR000680"
gene-bpuSUM 001669	lp35	pseudo		Pfam	protein matc	8	209	2.2E-56	+	.	Target=null 8 209;ID=match$3039_8_209;signature_desc=Borrelia lipoprotein;Name=PF00921;status=T;Dbxref="InterPro:IPR000680"
gene-bpuSUM 001672	lp35	gene	delta	Pfam	protein matc	44	324	1.5E-85	+	.	Target=null 44 324;ID=match$2227_44_324;signature_desc=Borrelia lipoprotein;Name=PF00921;status=T;Dbxref="InterPro:IPR000680"
gene-bpuSUM 001673	lp35	gene	beta	Pfam	protein matc	44	333	2E-46	+	.	Target=null 44 333;ID=match$270_44_333;signature_desc=Borrelia lipoprotein;Name=PF00921;status=T;Dbxref="InterPro:IPR000680"
gene-bpuSUM 001682	lp35	pseudo	delta	Pfam	protein matc	34	155	7.9E-37	+	.	Target=null 34 155;ID=match$1056_34_155;signature_desc=Borrelia lipoprotein;Name=PF00921;status=T;Dbxref="InterPro:IPR000680"
gene-bpuSUM 001682	lp35	pseudo		Pfam	protein matc	2	147	4.7E-39	+	.	Target=null 2 147;ID=match$1243_2_147;signature_desc=Borrelia lipoprotein;Name=PF00921;status=T;Dbxref="InterPro:IPR000680"
gene-bpuSUM 001684	lp35	gene	alpha	Pfam	protein matc	7	303	3.3E-68	+	.	Target=null 7 303;ID=match$3708_7_303;signature_desc=Borrelia lipoprotein;Name=PF00921;status=T;Dbxref="InterPro:IPR000680"
gene-bpuSUM 001685	lp35	pseudo	beta	Pfam	protein matc	14	260	3.7E-35	+	.	Target=null 14 260;ID=match$2337_14_260;signature_desc=Borrelia lipoprotein;Name=PF00921;status=T;Dbxref="InterPro:IPR000680"
gene-bpuSUM 001685	lp35	pseudo		Pfam	protein matc	2	38	2.7E-09	+	.	Target=null 2 38;ID=match$3350_2_38;signature_desc=Borrelia lipoprotein;Name=PF00921;status=T;Dbxref="InterPro:IPR000680"
gene-bpuSUM 001686	lp35	gene	alpha	Pfam	protein matc	49	341	6.6E-71	+	.	Target=null 49 341;ID=match$1292_49_341;signature_desc=Borrelia lipoprotein;Name=PF00921;status=T;Dbxref="InterPro:IPR000680"
gene-bpuSUM 001687	lp35	pseudo	beta	Pfam	protein matc	9	292	4E-47	+	.	Target=null 9 292;ID=match$1190_9_292;signature_desc=Borrelia lipoprotein;Name=PF00921;status=T;Dbxref="InterPro:IPR000680"
gene-bpuSUM 001691	lp39	pseudo	delta	Pfam	protein matc	34	60	0.000014	+	.	Target=null 34 60;ID=match$639_34_60;signature_desc=Borrelia lipoprotein;Name=PF00921;status=T;Dbxref="InterPro:IPR000680"
gene-bpuSUM 001691	lp39	pseudo		Pfam	protein matc	1	268	1.2E-75	+	.	Target=null 1 268;ID=match$1624_1_268;signature_desc=Borrelia lipoprotein;Name=PF00921;status=T;Dbxref="InterPro:IPR000680"
gene-bpuSUM 001704	lp39	pseudo	gamma	Pfam	protein matc	2	51	3.1E-10	+	.	Target=null 2 51;ID=match$1740_2_51;signature_desc=Borrelia lipoprotein;Name=PF00921;status=T;Dbxref="InterPro:IPR000680"
gene-bpuSUM 001704	lp39	pseudo		Pfam	protein matc	2	110	9.6E-19	+	.	Target=null 2 110;ID=match$2501_2_110;signature_desc=Borrelia lipoprotein;Name=PF00921;status=T;Dbxref="InterPro:IPR000680"
gene-bpuSUM 001705	lp39	gene	delta	Pfam	protein matc	1	270	6.8E-80	+	.	Target=null 1 270;ID=match$2737_1_270;signature_desc=Borrelia lipoprotein;Name=PF00921;status=T;Dbxref="InterPro:IPR000680"
gene-bpuSUM 001707	lp39	gene	delta	Pfam	protein matc	32	166	3.7E-36	+	.	Target=null 32 166;ID=match$3007_32_166;signature_desc=Borrelia lipoprotein;Name=PF00921;status=T;Dbxref="InterPro:IPR000680"
gene-bpuSUM 001709	lp39	gene	alpha	Pfam	protein matc	52	344	3.9E-70	+	.	Target=null 52 344;ID=match$2879_52_344;signature_desc=Borrelia lipoprotein;Name=PF00921;status=T;Dbxref="InterPro:IPR000680"
gene-bpuSUM 001710	lp39	gene	beta	Pfam	protein matc	23	304	3.1E-48	+	.	Target=null 23 304;ID=match$2043_23_304;signature_desc=Borrelia lipoprotein;Name=PF00921;status=T;Dbxref="InterPro:IPR000680"
gene-bpuSUM 001712	lp39	pseudo	delta	Pfam	protein matc	8	115	7.6E-26	+	.	Target=null 8 115;ID=match$1102_8_115;signature_desc=Borrelia lipoprotein;Name=PF00921;status=T;Dbxref="InterPro:IPR000680"
gene-bpuSUM 001712	lp39	pseudo		Pfam	protein matc	4	164	1.5E-37	+	.	Target=null 4 164;ID=match$505_4_164;signature_desc=Borrelia lipoprotein;Name=PF00921;status=T;Dbxref="InterPro:IPR000680"
gene-bpuSUM 001714	lp39	gene	gamma	Pfam	protein matc	34	308	1.6E-83	+	.	Target=null 34 308;ID=match$2038_34_308;signature_desc=Borrelia lipoprotein;Name=PF00921;status=T;Dbxref="InterPro:IPR000680"
gene-bpuSUM 001715	lp39	pseudo	beta	Pfam	protein matc	34	92	0.00000079	+	.	Target=null 34 92;ID=match$213_34_92;signature_desc=Borrelia lipoprotein;Name=PF00921;status=T;Dbxref="InterPro:IPR000680"
gene-bpuSUM 001715	lp39	pseudo		Pfam	protein matc	5	236	2.1E-29	+	.	Target=null 5 236;ID=match$3767_5_236;signature_desc=Borrelia lipoprotein;Name=PF00921;status=T;Dbxref="InterPro:IPR000680"
gene-bpuSUM 001716	lp39	gene	gamma	Pfam	protein matc	34	313	9E-89	+	.	Target=null 34 313;ID=match$1387_34_313;signature_desc=Borrelia lipoprotein;Name=PF00921;status=T;Dbxref="InterPro:IPR000680"
gene-bpuSUM 001718	lp39	pseudo	delta	Pfam	protein matc	19	288	8.5E-77	+	.	Target=null 19 288;ID=match$586_19_288;signature_desc=Borrelia lipoprotein;Name=PF00921;status=T;Dbxref="InterPro:IPR000680"
gene-bpuSUM 001720	lp39	gene	delta	Pfam	protein matc	44	334	2.8E-88	+	.	Target=null 44 334;ID=match$510_44_334;signature_desc=Borrelia lipoprotein;Name=PF00921;status=T;Dbxref="InterPro:IPR000680"
gene-bpuSUM 001721	lp39	gene	gamma	Pfam	protein matc	44	318	2.1E-81	+	.	Target=null 44 318;ID=match$188_44_318;signature_desc=Borrelia lipoprotein;Name=PF00921;status=T;Dbxref="InterPro:IPR000680"
gene-bpuSUM 001723	lp39	gene	delta	Pfam	protein matc	33	306	5E-85	+	.	Target=null 33 306;ID=match$3192_33_306;signature_desc=Borrelia lipoprotein;Name=PF00921;status=T;Dbxref="InterPro:IPR000680"
gene-bpuSUM 001726	lp39	gene	gamma	Pfam	protein matc	44	330	4.6E-91	+	.	Target=null 44 330;ID=match$3180_44_330;signature_desc=Borrelia lipoprotein;Name=PF00921;status=T;Dbxref="InterPro:IPR000680"
gene-bpuSUM 001727	lp39	pseudo	alpha	Pfam	protein matc	53	335	7E-69	+	.	Target=null 53 335;ID=match$1219_53_335;signature_desc=Borrelia lipoprotein;Name=PF00921;status=T;Dbxref="InterPro:IPR000680"
gene-bpuSUM 001741	lp41	pseudo	gamma	Pfam	protein matc	34	121	3.6E-26	+	.	Target=null 34 121;ID=match$325_34_121;signature_desc=Borrelia lipoprotein;Name=PF00921;status=T;Dbxref="InterPro:IPR000680"
gene-bpuSUM 001741	lp41	pseudo		Pfam	protein matc	12	61	3.9E-09	+	.	Target=null 12 61;ID=match$1681_12_61;signature_desc=Borrelia lipoprotein;Name=PF00921;status=T;Dbxref="InterPro:IPR000680"
gene-bpuSUM 001741	lp41	pseudo		Pfam	protein matc	22	150	2.4E-35	+	.	Target=null 22 150;ID=match$857_22_150;signature_desc=Borrelia lipoprotein;Name=PF00921;status=T;Dbxref="InterPro:IPR000680"
gene-bpuSUM 001748	lp41	pseudo	delta	Pfam	protein matc	34	235	1.1E-48	+	.	Target=null 34 235;ID=match$267_34_235;signature_desc=Borrelia lipoprotein;Name=PF00921;status=T;Dbxref="InterPro:IPR000680"
gene-bpuSUM 001748	lp41	pseudo		Pfam	protein matc	3	45	2E-13	+	.	Target=null 3 45;ID=match$3086_3_45;signature_desc=Borrelia lipoprotein;Name=PF00921;status=T;Dbxref="InterPro:IPR000680"
gene-bpuSUM 001749	lp41	gene	gamma	Pfam	protein matc	44	326	4.8E-87	+	.	Target=null 44 326;ID=match$965_44_326;signature_desc=Borrelia lipoprotein;Name=PF00921;status=T;Dbxref="InterPro:IPR000680"
gene-bpuSUM 001751	lp41	pseudo	alpha	Pfam	protein matc	47	79	0.00000084	+	.	Target=null 47 79;ID=match$2029_47_79;signature_desc=Borrelia lipoprotein;Name=PF00921;status=T;Dbxref="InterPro:IPR000680"
gene-bpuSUM 001751	lp41	pseudo		Pfam	protein matc	5	266	1.6E-52	+	.	Target=null 5 266;ID=match$11_5_266;signature_desc=Borrelia lipoprotein;Name=PF00921;status=T;Dbxref="InterPro:IPR000680"
gene-bpuSUM 001752	lp41	gene	delta	Pfam	protein matc	1	250	4E-69	+	.	Target=null 1 250;ID=match$1918_1_250;signature_desc=Borrelia lipoprotein;Name=PF00921;status=T;Dbxref="InterPro:IPR000680"
gene-bpuSUM 001753	lp41	gene	gamma	Pfam	protein matc	34	319	1E-83	+	.	Target=null 34 319;ID=match$2298_34_319;signature_desc=Borrelia lipoprotein;Name=PF00921;status=T;Dbxref="InterPro:IPR000680"
gene-bpuSUM 001757	lp41	gene	beta	Pfam	protein matc	44	331	1E-46	+	.	Target=null 44 331;ID=match$1726_44_331;signature_desc=Borrelia lipoprotein;Name=PF00921;status=T;Dbxref="InterPro:IPR000680"
gene-bpuSUM 001758	lp41	gene	delta	Pfam	protein matc	45	323	1.2E-88	+	.	Target=null 45 323;ID=match$2596_45_323;signature_desc=Borrelia lipoprotein;Name=PF00921;status=T;Dbxref="InterPro:IPR000680"
gene-bpuSUM 001759	lp41	gene	alpha	Pfam	protein matc	42	333	2.6E-70	+	.	Target=null 42 333;ID=match$2110_42_333;signature_desc=Borrelia lipoprotein;Name=PF00921;status=T;Dbxref="InterPro:IPR000680"
gene-bpuSUM 001760	lp41	gene	beta	Pfam	protein matc	13	301	4.2E-50	+	.	Target=null 13 301;ID=match$1695_13_301;signature_desc=Borrelia lipoprotein;Name=PF00921;status=T;Dbxref="InterPro:IPR000680"
gene-bpuSUM 001761	lp41	gene	gamma	Pfam	protein matc	44	318	1.9E-83	+	.	Target=null 44 318;ID=match$2141_44_318;signature_desc=Borrelia lipoprotein;Name=PF00921;status=T;Dbxref="InterPro:IPR000680"
gene-bpuSUM 001762	lp41	gene	alpha	Pfam	protein matc	52	344	2.2E-70	+	.	Target=null 52 344;ID=match$3505_52_344;signature_desc=Borrelia lipoprotein;Name=PF00921;status=T;Dbxref="InterPro:IPR000680"
gene-bpuSUM 001763	lp41	pseudo	beta	Pfam	protein matc	23	178	2.2E-15	+	.	Target=null 23 178;ID=match$2199_23_178;signature_desc=Borrelia lipoprotein;Name=PF00921;status=T;Dbxref="InterPro:IPR000680"
gene-bpuSUM 001763	lp41	pseudo		Pfam	protein matc	3	124	1.8E-25	+	.	Target=null 3 124;ID=match$1978_3_124;signature_desc=Borrelia lipoprotein;Name=PF00921;status=T;Dbxref="InterPro:IPR000680"
gene-bpuSUM 001765	lp41	pseudo	alpha	Pfam	protein matc	56	305	6E-55	+	.	Target=null 56 305;ID=match$2667_56_305;signature_desc=Borrelia lipoprotein;Name=PF00921;status=T;Dbxref="InterPro:IPR000680"
gene-bpuSUM 001765	lp41	pseudo		Pfam	protein matc	2	34	0.00000061	+	.	Target=null 2 34;ID=match$1456_2_34;signature_desc=Borrelia lipoprotein;Name=PF00921;status=T;Dbxref="InterPro:IPR000680"
gene-bpuSUM 001766	lp41	pseudo	delta	Pfam	protein matc	1	265	7.3E-77	+	.	Target=null 1 265;ID=match$265_1_265;signature_desc=Borrelia lipoprotein;Name=PF00921;status=T;Dbxref="InterPro:IPR000680"
gene-bpuSUM 001767	lp41	pseudo	gamma	Pfam	protein matc	34	82	2.6E-16	+	.	Target=null 34 82;ID=match$3349_34_82;signature_desc=Borrelia lipoprotein;Name=PF00921;status=T;Dbxref="InterPro:IPR000680"
gene-bpuSUM 001767	lp41	pseudo		Pfam	protein matc	3	46	0.00000052	+	.	Target=null 3 46;ID=match$541_3_46;signature_desc=Borrelia lipoprotein;Name=PF00921;status=T;Dbxref="InterPro:IPR000680"
gene-bpuSUM 001767	lp41	pseudo		Pfam	protein matc	19	197	4.4E-45	+	.	Target=null 19 197;ID=match$884_19_197;signature_desc=Borrelia lipoprotein;Name=PF00921;status=T;Dbxref="InterPro:IPR000680"
gene-bpuSUM 001775	lp46	pseudo	alpha	Pfam	protein matc	8	62	2.2E-13	+	.	Target=null 8 62;ID=match$2316_8_62;signature_desc=Borrelia lipoprotein;Name=PF00921;status=T;Dbxref="InterPro:IPR000680"
gene-bpuSUM 001775	lp46	pseudo		Pfam	protein matc	11	245	5.1E-51	+	.	Target=null 11 245;ID=match$2701_11_245;signature_desc=Borrelia lipoprotein;Name=PF00921;status=T;Dbxref="InterPro:IPR000680"
gene-bpuSUM 001776	lp46	pseudo	gamma	Pfam	protein matc	19	150	3.4E-44	+	.	Target=null 19 150;ID=match$1917_19_150;signature_desc=Borrelia lipoprotein;Name=PF00921;status=T;Dbxref="InterPro:IPR000680"
gene-bpuSUM 001776	lp46	pseudo		Pfam	protein matc	5	163	6.6E-42	+	.	Target=null 5 163;ID=match$1951_5_163;signature_desc=Borrelia lipoprotein;Name=PF00921;status=T;Dbxref="InterPro:IPR000680"
gene-bpuSUM 001777	lp46	gene	delta	Pfam	protein matc	30	315	7E-85	+	.	Target=null 30 315;ID=match$1685_30_315;signature_desc=Borrelia lipoprotein;Name=PF00921;status=T;Dbxref="InterPro:IPR000680"
gene-bpuSUM 001779	lp46	gene	delta	Pfam	protein matc	45	329	3.6E-85	+	.	Target=null 45 329;ID=match$104_45_329;signature_desc=Borrelia lipoprotein;Name=PF00921;status=T;Dbxref="InterPro:IPR000680"
gene-bpuSUM 001780	lp46	gene	delta	Pfam	protein matc	19	309	4.5E-88	+	.	Target=null 19 309;ID=match$1596_19_309;signature_desc=Borrelia lipoprotein;Name=PF00921;status=T;Dbxref="InterPro:IPR000680"
gene-bpuSUM 001782	lp46	gene	delta	Pfam	protein matc	45	323	2.4E-76	+	.	Target=null 45 323;ID=match$3100_45_323;signature_desc=Borrelia lipoprotein;Name=PF00921;status=T;Dbxref="InterPro:IPR000680"
gene-bpuSUM 001783	lp46	pseudo	gamma	Pfam	protein matc	34	191	6.4E-43	+	.	Target=null 34 191;ID=match$1584_34_191;signature_desc=Borrelia lipoprotein;Name=PF00921;status=T;Dbxref="InterPro:IPR000680"
gene-bpuSUM 001783	lp46	pseudo		Pfam	protein matc	3	133	2.8E-38	+	.	Target=null 3 133;ID=match$1439_3_133;signature_desc=Borrelia lipoprotein;Name=PF00921;status=T;Dbxref="InterPro:IPR000680"
gene-bpuSUM 001785	lp46	gene	beta	Pfam	protein matc	20	309	1.9E-42	+	.	Target=null 20 309;ID=match$2954_20_309;signature_desc=Borrelia lipoprotein;Name=PF00921;status=T;Dbxref="InterPro:IPR000680"
gene-bpuSUM 001786	lp46	gene	alpha	Pfam	protein matc	7	302	9.9E-69	+	.	Target=null 7 302;ID=match$1453_7_302;signature_desc=Borrelia lipoprotein;Name=PF00921;status=T;Dbxref="InterPro:IPR000680"
gene-bpuSUM 001790	lp46	gene	delta	Pfam	protein matc	45	327	6.8E-86	+	.	Target=null 45 327;ID=match$2127_45_327;signature_desc=Borrelia lipoprotein;Name=PF00921;status=T;Dbxref="InterPro:IPR000680"
gene-bpuSUM 001791	lp46	gene	beta	Pfam	protein matc	34	321	2.1E-43	+	.	Target=null 34 321;ID=match$1084_34_321;signature_desc=Borrelia lipoprotein;Name=PF00921;status=T;Dbxref="InterPro:IPR000680"
gene-bpuSUM 001792	lp46	gene	alpha	Pfam	protein matc	49	345	9.2E-72	+	.	Target=null 49 345;ID=match$1442_49_345;signature_desc=Borrelia lipoprotein;Name=PF00921;status=T;Dbxref="InterPro:IPR000680"
gene-bpuSUM 001793	lp46	gene	beta	Pfam	protein matc	56	344	3.8E-42	+	.	Target=null 56 344;ID=match$1277_56_344;signature_desc=Borrelia lipoprotein;Name=PF00921;status=T;Dbxref="InterPro:IPR000680"
gene-bpuSUM 001794	lp46	pseudo	beta	Pfam	protein matc	19	61	0.0000031	+	.	Target=null 19 61;ID=match$933_19_61;signature_desc=Borrelia lipoprotein;Name=PF00921;status=T;Dbxref="InterPro:IPR000680"
gene-bpuSUM 001794	lp46	pseudo		Pfam	protein matc	11	197	1.1E-31	+	.	Target=null 11 197;ID=match$2499_11_197;signature_desc=Borrelia lipoprotein;Name=PF00921;status=T;Dbxref="InterPro:IPR000680"
gene-bpuSUM 001798	lp46	pseudo	gamma	Pfam	protein matc	51	100	3.7E-08	+	.	Target=null 51 100;ID=match$1796_51_100;signature_desc=Borrelia lipoprotein;Name=PF00921;status=T;Dbxref="InterPro:IPR000680"
gene-bpuSUM 001800	lp46	gene	alpha	Pfam	protein matc	7	301	1.4E-71	+	.	Target=null 7 301;ID=match$2463_7_301;signature_desc=Borrelia lipoprotein;Name=PF00921;status=T;Dbxref="InterPro:IPR000680"
gene-bpuSUM 001803	lp46	pseudo	gamma	Pfam	protein matc	45	178	1E-43	+	.	Target=null 45 178;ID=match$57_45_178;signature_desc=Borrelia lipoprotein;Name=PF00921;status=T;Dbxref="InterPro:IPR000680"
gene-bpuSUM 001803	lp46	pseudo		Pfam	protein matc	13	161	1.9E-35	+	.	Target=null 13 161;ID=match$3110_13_161;signature_desc=Borrelia lipoprotein;Name=PF00921;status=T;Dbxref="InterPro:IPR000680"
gene-bpuSUM 001804	lp46	gene	beta	Pfam	protein matc	45	331	2E-47	+	.	Target=null 45 331;ID=match$2111_45_331;signature_desc=Borrelia lipoprotein;Name=PF00921;status=T;Dbxref="InterPro:IPR000680"
gene-bpuSUM 001806	lp46	gene	delta	Pfam	protein matc	1	61	1.1E-17	+	.	Target=null 1 61;ID=match$697_1_61;signature_desc=Borrelia lipoprotein;Name=PF00921;status=T;Dbxref="InterPro:IPR000680"
gene-bpuSUM 001812	lp46	gene	delta	Pfam	protein matc	8	42	0.0000034	+	.	Target=null 8 42;ID=match$3148_8_42;signature_desc=Borrelia lipoprotein;Name=PF00921;status=T;Dbxref="InterPro:IPR000680"
gene-bpuSUM 001841	lp47	gene	alpha	Pfam	protein matc	7	302	9.9E-69	+	.	Target=null 7 302;ID=match$1451_7_302;signature_desc=Borrelia lipoprotein;Name=PF00921;status=T;Dbxref="InterPro:IPR000680"
gene-bpuSUM 001842	lp47	gene	beta	Pfam	protein matc	20	309	1.9E-42	+	.	Target=null 20 309;ID=match$2956_20_309;signature_desc=Borrelia lipoprotein;Name=PF00921;status=T;Dbxref="InterPro:IPR000680"
gene-bpuSUM 001844	lp47	pseudo	gamma	Pfam	protein matc	34	191	6.4E-43	+	.	Target=null 34 191;ID=match$1586_34_191;signature_desc=Borrelia lipoprotein;Name=PF00921;status=T;Dbxref="InterPro:IPR000680"
gene-bpuSUM 001844	lp47	pseudo		Pfam	protein matc	3	133	2.8E-38	+	.	Target=null 3 133;ID=match$1441_3_133;signature_desc=Borrelia lipoprotein;Name=PF00921;status=T;Dbxref="InterPro:IPR000680"
gene-bpuSUM 001845	lp47	gene	delta	Pfam	protein matc	45	323	2.4E-76	+	.	Target=null 45 323;ID=match$3098_45_323;signature_desc=Borrelia lipoprotein;Name=PF00921;status=T;Dbxref="InterPro:IPR000680"
gene-bpuSUM 001847	lp47	gene	delta	Pfam	protein matc	19	309	4.5E-88	+	.	Target=null 19 309;ID=match$1598_19_309;signature_desc=Borrelia lipoprotein;Name=PF00921;status=T;Dbxref="InterPro:IPR000680"
gene-bpuSUM 001848	lp47	gene	delta	Pfam	protein matc	45	329	3.6E-85	+	.	Target=null 45 329;ID=match$106_45_329;signature_desc=Borrelia lipoprotein;Name=PF00921;status=T;Dbxref="InterPro:IPR000680"
gene-bpuSUM 001850	lp47	gene	delta	Pfam	protein matc	30	315	7E-85	+	.	Target=null 30 315;ID=match$1691_30_315;signature_desc=Borrelia lipoprotein;Name=PF00921;status=T;Dbxref="InterPro:IPR000680"
gene-bpuSUM 001851	lp47	gene	gamma	Pfam	protein matc	44	330	4.6E-91	+	.	Target=null 44 330;ID=match$3184_44_330;signature_desc=Borrelia lipoprotein;Name=PF00921;status=T;Dbxref="InterPro:IPR000680"
gene-bpuSUM 001852	lp47	pseudo	alpha	Pfam	protein matc	53	335	7E-69	+	.	Target=null 53 335;ID=match$1215_53_335;signature_desc=Borrelia lipoprotein;Name=PF00921;status=T;Dbxref="InterPro:IPR000680"
gene-bpuSUM 001859	lp57	pseudo	alpha	Pfam	protein matc	53	335	7E-69	+	.	Target=null 53 335;ID=match$1217_53_335;signature_desc=Borrelia lipoprotein;Name=PF00921;status=T;Dbxref="InterPro:IPR000680"
gene-bpuSUM 001860	lp57	gene	gamma	Pfam	protein matc	44	330	4.6E-91	+	.	Target=null 44 330;ID=match$3182_44_330;signature_desc=Borrelia lipoprotein;Name=PF00921;status=T;Dbxref="InterPro:IPR000680"
gene-bpuSUM 001861	lp57	gene	delta	Pfam	protein matc	30	315	7E-85	+	.	Target=null 30 315;ID=match$1689_30_315;signature_desc=Borrelia lipoprotein;Name=PF00921;status=T;Dbxref="InterPro:IPR000680"
gene-bpuSUM 001863	lp57	gene	delta	Pfam	protein matc	19	303	1.4E-87	+	.	Target=null 19 303;ID=match$2287_19_303;signature_desc=Borrelia lipoprotein;Name=PF00921;status=T;Dbxref="InterPro:IPR000680"
gene-bpuSUM 001865	lp57	gene	beta	Pfam	protein matc	61	349	2E-45	+	.	Target=null 61 349;ID=match$635_61_349;signature_desc=Borrelia lipoprotein;Name=PF00921;status=T;Dbxref="InterPro:IPR000680"
gene-bpuSUM 001866	lp57	gene	beta	Pfam	protein matc	5	294	3.8E-41	+	.	Target=null 5 294;ID=match$1968_5_294;signature_desc=Borrelia lipoprotein;Name=PF00921;status=T;Dbxref="InterPro:IPR000680"
gene-bpuSUM 001867	lp57	gene	alpha	Pfam	protein matc	22	311	1E-74	+	.	Target=null 22 311;ID=match$331_22_311;signature_desc=Borrelia lipoprotein;Name=PF00921;status=T;Dbxref="InterPro:IPR000680"
gene-bpuSUM 001868	lp57	gene	gamma	Pfam	protein matc	44	326	3.5E-91	+	.	Target=null 44 326;ID=match$1104_44_326;signature_desc=Borrelia lipoprotein;Name=PF00921;status=T;Dbxref="InterPro:IPR000680"
gene-bpuSUM 001871	lp57	gene	gamma	Pfam	protein matc	44	323	6.8E-87	+	.	Target=null 44 323;ID=match$2188_44_323;signature_desc=Borrelia lipoprotein;Name=PF00921;status=T;Dbxref="InterPro:IPR000680"
gene-bpuSUM 001872	lp57	pseudo	delta	Pfam	protein matc	19	167	1.5E-38	+	.	Target=null 19 167;ID=match$2238_19_167;signature_desc=Borrelia lipoprotein;Name=PF00921;status=T;Dbxref="InterPro:IPR000680"
gene-bpuSUM 001872	lp57	pseudo		Pfam	protein matc	2	114	1.4E-30	+	.	Target=null 2 114;ID=match$2893_2_114;signature_desc=Borrelia lipoprotein;Name=PF00921;status=T;Dbxref="InterPro:IPR000680"
gene-bpuSUM 001874	lp57	gene	delta	Pfam	protein matc	45	324	8.7E-87	+	.	Target=null 45 324;ID=match$1230_45_324;signature_desc=Borrelia lipoprotein;Name=PF00921;status=T;Dbxref="InterPro:IPR000680"
gene-bpuSUM 001876	lp57	gene	delta	Pfam	protein matc	34	315	1.5E-84	+	.	Target=null 34 315;ID=match$1654_34_315;signature_desc=Borrelia lipoprotein;Name=PF00921;status=T;Dbxref="InterPro:IPR000680"
gene-bpuSUM 001890	lp57	gene	gamma	Pfam	protein matc	19	295	3.2E-83	+	.	Target=null 19 295;ID=match$2896_19_295;signature_desc=Borrelia lipoprotein;Name=PF00921;status=T;Dbxref="InterPro:IPR000680"
gene-bpuSUM 001891	lp57	gene	gamma	Pfam	protein matc	38	328	3.6E-92	+	.	Target=null 38 328;ID=match$3420_38_328;signature_desc=Borrelia lipoprotein;Name=PF00921;status=T;Dbxref="InterPro:IPR000680"
gene-bpuSUM 001892	lp57	gene	delta	Pfam	protein matc	19	292	3.5E-80	+	.	Target=null 19 292;ID=match$3472_19_292;signature_desc=Borrelia lipoprotein;Name=PF00921;status=T;Dbxref="InterPro:IPR000680"
gene-bpuSUM 001893	lp57	gene	beta	Pfam	protein matc	45	333	3E-44	+	.	Target=null 45 333;ID=match$508_45_333;signature_desc=Borrelia lipoprotein;Name=PF00921;status=T;Dbxref="InterPro:IPR000680"
gene-bpuSUM 001895	lp57	pseudo	gamma	Pfam	protein matc	1	268	3.9E-78	+	.	Target=null 1 268;ID=match$2469_1_268;signature_desc=Borrelia lipoprotein;Name=PF00921;status=T;Dbxref="InterPro:IPR000680"
gene-bpuSUM 001897	lp57	gene	alpha	Pfam	protein matc	7	300	1.7E-70	+	.	Target=null 7 300;ID=match$1488_7_300;signature_desc=Borrelia lipoprotein;Name=PF00921;status=T;Dbxref="InterPro:IPR000680"
gene-bpuSUM 001898	lp57	gene	delta	Pfam	protein matc	34	317	4.7E-85	+	.	Target=null 34 317;ID=match$2446_34_317;signature_desc=Borrelia lipoprotein;Name=PF00921;status=T;Dbxref="InterPro:IPR000680"
gene-bpuSUM 001900	lp57	gene	alpha	Pfam	protein matc	7	301	5E-67	+	.	Target=null 7 301;ID=match$385_7_301;signature_desc=Borrelia lipoprotein;Name=PF00921;status=T;Dbxref="InterPro:IPR000680"
gene-bpuSUM 001901	lp57	gene	beta	Pfam	protein matc	35	321	4.5E-49	+	.	Target=null 35 321;ID=match$3657_35_321;signature_desc=Borrelia lipoprotein;Name=PF00921;status=T;Dbxref="InterPro:IPR000680"
gene-bpuSUM 001902	lp57	gene	alpha	Pfam	protein matc	42	337	2.2E-65	+	.	Target=null 42 337;ID=match$1328_42_337;signature_desc=Borrelia lipoprotein;Name=PF00921;status=T;Dbxref="InterPro:IPR000680"
gene-bpuSUM 001903	lp57	gene	beta	Pfam	protein matc	20	307	1.2E-51	+	.	Target=null 20 307;ID=match$3222_20_307;signature_desc=Borrelia lipoprotein;Name=PF00921;status=T;Dbxref="InterPro:IPR000680"
gene-bpuSUM 001904	lp57	gene	gamma	Pfam	protein matc	34	308	1.9E-81	+	.	Target=null 34 308;ID=match$2448_34_308;signature_desc=Borrelia lipoprotein;Name=PF00921;status=T;Dbxref="InterPro:IPR000680"
gene-bpuSUM 001906	lp57	gene	delta	Pfam	protein matc	33	306	5E-85	+	.	Target=null 33 306;ID=match$3190_33_306;signature_desc=Borrelia lipoprotein;Name=PF00921;status=T;Dbxref="InterPro:IPR000680"
gene-bpuSUM 001909	lp57	gene	gamma	Pfam	protein matc	44	330	4.6E-91	+	.	Target=null 44 330;ID=match$3176_44_330;signature_desc=Borrelia lipoprotein;Name=PF00921;status=T;Dbxref="InterPro:IPR000680"
gene-bpuSUM 001910	lp57	pseudo	alpha	Pfam	protein matc	53	107	9.8E-13	+	.	Target=null 53 107;ID=match$21_53_107;signature_desc=Borrelia lipoprotein;Name=PF00921;status=T;Dbxref="InterPro:IPR000680"
gene-bpuSUM 001910	lp57	pseudo		Pfam	protein matc	11	245	5.1E-51	+	.	Target=null 11 245;ID=match$2699_11_245;signature_desc=Borrelia lipoprotein;Name=PF00921;status=T;Dbxref="InterPro:IPR000680"
gene-bpuSUM 001917	lp65	pseudo	gamma	Pfam	protein matc	35	223	1.3E-54	+	.	Target=null 35 223;ID=match$2864_35_223;signature_desc=Borrelia lipoprotein;Name=PF00921;status=T;Dbxref="InterPro:IPR000680"
gene-bpuSUM 001917	lp65	pseudo		Pfam	protein matc	8	89	2.3E-27	+	.	Target=null 8 89;ID=match$253_8_89;signature_desc=Borrelia lipoprotein;Name=PF00921;status=T;Dbxref="InterPro:IPR000680"
gene-bpuSUM 001919	lp65	gene	delta	Pfam	protein matc	45	326	1.4E-88	+	.	Target=null 45 326;ID=match$3067_45_326;signature_desc=Borrelia lipoprotein;Name=PF00921;status=T;Dbxref="InterPro:IPR000680"
gene-bpuSUM 001922	lp65	gene	delta	Pfam	protein matc	45	325	1.1E-82	+	.	Target=null 45 325;ID=match$2992_45_325;signature_desc=Borrelia lipoprotein;Name=PF00921;status=T;Dbxref="InterPro:IPR000680"
gene-bpuSUM 001923	lp65	gene	alpha	Pfam	protein matc	39	328	5.5E-68	+	.	Target=null 39 328;ID=match$849_39_328;signature_desc=Borrelia lipoprotein;Name=PF00921;status=T;Dbxref="InterPro:IPR000680"
gene-bpuSUM 001924	lp65	gene	delta	Pfam	protein matc	44	333	1.6E-86	+	.	Target=null 44 333;ID=match$3143_44_333;signature_desc=Borrelia lipoprotein;Name=PF00921;status=T;Dbxref="InterPro:IPR000680"
gene-bpuSUM 001926	lp65	pseudo	delta	Pfam	protein matc	45	129	2.7E-26	+	.	Target=null 45 129;ID=match$2847_45_129;signature_desc=Borrelia lipoprotein;Name=PF00921;status=T;Dbxref="InterPro:IPR000680"
gene-bpuSUM 001926	lp65	pseudo		Pfam	protein matc	16	209	1.3E-55	+	.	Target=null 16 209;ID=match$517_16_209;signature_desc=Borrelia lipoprotein;Name=PF00921;status=T;Dbxref="InterPro:IPR000680"
gene-bpuSUM 001927	lp65	gene	beta	Pfam	protein matc	45	328	5.1E-48	+	.	Target=null 45 328;ID=match$3378_45_328;signature_desc=Borrelia lipoprotein;Name=PF00921;status=T;Dbxref="InterPro:IPR000680"
gene-bpuSUM 001928	lp65	pseudo	beta	Pfam	protein matc	20	107	1.5E-09	+	.	Target=null 20 107;ID=match$514_20_107;signature_desc=Borrelia lipoprotein;Name=PF00921;status=T;Dbxref="InterPro:IPR000680"
gene-bpuSUM 001929	lp65	pseudo		Pfam	protein matc	7	85	8.1E-16	+	.	Target=null 7 85;ID=match$274_7_85;signature_desc=Borrelia lipoprotein;Name=PF00921;status=T;Dbxref="InterPro:IPR000680"
gene-bpuSUM 001929	lp65	pseudo	alpha	Pfam	protein matc	6	206	4.5E-47	+	.	Target=null 6 206;ID=match$2407_6_206;signature_desc=Borrelia lipoprotein;Name=PF00921;status=T;Dbxref="InterPro:IPR000680"
gene-bpuSUM 001931	lp65	pseudo	beta	Pfam	protein matc	16	150	3.4E-19	+	.	Target=null 16 150;ID=match$646_16_150;signature_desc=Borrelia lipoprotein;Name=PF00921;status=T;Dbxref="InterPro:IPR000680"
gene-bpuSUM 001932	lp65	gene	alpha	Pfam	protein matc	49	345	6.6E-71	+	.	Target=null 49 345;ID=match$2495_49_345;signature_desc=Borrelia lipoprotein;Name=PF00921;status=T;Dbxref="InterPro:IPR000680"
gene-bpuSUM 001934	lp65	gene	delta	Pfam	protein matc	30	316	1.4E-89	+	.	Target=null 30 316;ID=match$2135_30_316;signature_desc=Borrelia lipoprotein;Name=PF00921;status=T;Dbxref="InterPro:IPR000680"
gene-bpuSUM 001936	lp65	pseudo	gamma	Pfam	protein matc	21	233	4.9E-59	+	.	Target=null 21 233;ID=match$1790_21_233;signature_desc=Borrelia lipoprotein;Name=PF00921;status=T;Dbxref="InterPro:IPR000680"
gene-bpuSUM 001936	lp65	pseudo		Pfam	protein matc	24	87	2E-24	+	.	Target=null 24 87;ID=match$2168_24_87;signature_desc=Borrelia lipoprotein;Name=PF00921;status=T;Dbxref="InterPro:IPR000680"
gene-bpuSUM 001938	lp65	gene	delta	Pfam	protein matc	8	298	1.6E-83	+	.	Target=null 8 298;ID=match$1261_8_298;signature_desc=Borrelia lipoprotein;Name=PF00921;status=T;Dbxref="InterPro:IPR000680"
gene-bpuSUM 001953	lp65	gene	delta	Pfam	protein matc	42	326	1.6E-83	+	.	Target=null 42 326;ID=match$2827_42_326;signature_desc=Borrelia lipoprotein;Name=PF00921;status=T;Dbxref="InterPro:IPR000680"
gene-bpuSUM 001957	lp65	pseudo	beta	Pfam	protein matc	19	61	0.0000031	+	.	Target=null 19 61;ID=match$931_19_61;signature_desc=Borrelia lipoprotein;Name=PF00921;status=T;Dbxref="InterPro:IPR000680"
gene-bpuSUM 001957	lp65	pseudo		Pfam	protein matc	11	197	1.1E-31	+	.	Target=null 11 197;ID=match$2497_11_197;signature_desc=Borrelia lipoprotein;Name=PF00921;status=T;Dbxref="InterPro:IPR000680"
gene-bpuSUM 001958	lp65	gene	beta	Pfam	protein matc	56	344	3.8E-42	+	.	Target=null 56 344;ID=match$1279_56_344;signature_desc=Borrelia lipoprotein;Name=PF00921;status=T;Dbxref="InterPro:IPR000680"
gene-bpuSUM 001959	lp65	gene	alpha	Pfam	protein matc	49	345	9.2E-72	+	.	Target=null 49 345;ID=match$1444_49_345;signature_desc=Borrelia lipoprotein;Name=PF00921;status=T;Dbxref="InterPro:IPR000680"
gene-bpuSUM 001960	lp65	gene	beta	Pfam	protein matc	37	324	2.2E-43	+	.	Target=null 37 324;ID=match$3311_37_324;signature_desc=Borrelia lipoprotein;Name=PF00921;status=T;Dbxref="InterPro:IPR000680"
gene-bpuSUM 001961	lp65	gene	delta	Pfam	protein matc	45	327	6.8E-86	+	.	Target=null 45 327;ID=match$2129_45_327;signature_desc=Borrelia lipoprotein;Name=PF00921;status=T;Dbxref="InterPro:IPR000680"
gene-bpuSUM 001965	lp65	gene	alpha	Pfam	protein matc	7	302	9.9E-69	+	.	Target=null 7 302;ID=match$1449_7_302;signature_desc=Borrelia lipoprotein;Name=PF00921;status=T;Dbxref="InterPro:IPR000680"
gene-bpuSUM 001966	lp65	gene	beta	Pfam	protein matc	20	309	1.9E-42	+	.	Target=null 20 309;ID=match$2958_20_309;signature_desc=Borrelia lipoprotein;Name=PF00921;status=T;Dbxref="InterPro:IPR000680"
gene-bpuSUM 001968	lp65	pseudo	gamma	Pfam	protein matc	34	191	6.4E-43	+	.	Target=null 34 191;ID=match$1588_34_191;signature_desc=Borrelia lipoprotein;Name=PF00921;status=T;Dbxref="InterPro:IPR000680"
gene-bpuSUM 001968	lp65	pseudo		Pfam	protein matc	3	133	2.8E-38	+	.	Target=null 3 133;ID=match$1437_3_133;signature_desc=Borrelia lipoprotein;Name=PF00921;status=T;Dbxref="InterPro:IPR000680"
gene-bpuSUM 001969	lp65	gene	delta	Pfam	protein matc	45	323	2.4E-76	+	.	Target=null 45 323;ID=match$3102_45_323;signature_desc=Borrelia lipoprotein;Name=PF00921;status=T;Dbxref="InterPro:IPR000680"
gene-bpuSUM 001971	lp65	gene	delta	Pfam	protein matc	19	309	4.5E-88	+	.	Target=null 19 309;ID=match$1594_19_309;signature_desc=Borrelia lipoprotein;Name=PF00921;status=T;Dbxref="InterPro:IPR000680"
gene-bpuSUM 001972	lp65	gene	delta	Pfam	protein matc	45	329	3.6E-85	+	.	Target=null 45 329;ID=match$108_45_329;signature_desc=Borrelia lipoprotein;Name=PF00921;status=T;Dbxref="InterPro:IPR000680"
gene-bpuSUM 001974	lp65	gene	delta	Pfam	protein matc	30	315	7E-85	+	.	Target=null 30 315;ID=match$1687_30_315;signature_desc=Borrelia lipoprotein;Name=PF00921;status=T;Dbxref="InterPro:IPR000680"
gene-bpuSUM 001975	lp65	gene	gamma	Pfam	protein matc	44	330	4.6E-91	+	.	Target=null 44 330;ID=match$3178_44_330;signature_desc=Borrelia lipoprotein;Name=PF00921;status=T;Dbxref="InterPro:IPR000680"
gene-bpuSUM 001976	lp65	pseudo	alpha	Pfam	protein matc	53	335	7E-69	+	.	Target=null 53 335;ID=match$1213_53_335;signature_desc=Borrelia lipoprotein;Name=PF00921;status=T;Dbxref="InterPro:IPR000680"

Vsp	replicon	gene/pseudo									
gene-bpSLO 001244	lp28	gene	Pfam		protein matc	39	206	1.7E-67	+	.	Target=null 39 206;ID=match$2082_39_206;signature_desc=Lipoprotein;Name=PF01441;status=T;Dbxref="InterPro:IPR001800"
gene-bpSLO 001281	Lp30-1	gene	Pfam		protein matc	29	195	7.8E-56	+	.	Target=null 29 195;ID=match$1518_29_195;signature_desc=Lipoprotein;Name=PF01441;status=T;Dbxref="InterPro:IPR001800"
gene-bpSLO 001340	lp32	gene	Pfam		protein matc	40	205	3E-63	+	.	Target=null 40 205;ID=match$1120_40_205;signature_desc=Lipoprotein;Name=PF01441;status=T;Dbxref="InterPro:IPR001800"
gene-bpSLO 001360	Lp34-1	gene	Pfam		protein matc	40	209	1.4E-58	+	.	Target=null 40 209;ID=match$2441_40_209;signature_desc=Lipoprotein;Name=PF01441;status=T;Dbxref="InterPro:IPR001800"
gene-bpSLO 001374	Lp34-1	gene	Pfam		protein matc	39	205	1.6E-58	+	.	Target=null 39 205;ID=match$1090_39_205;signature_desc=Lipoprotein;Name=PF01441;status=T;Dbxref="InterPro:IPR001800"
gene-bpSLO 001378	Lp34-1	gene	Pfam		protein matc	40	208	3.7E-56	+	.	Target=null 40 208;ID=match$1034_40_208;signature_desc=Lipoprotein;Name=PF01441;status=T;Dbxref="InterPro:IPR001800"
gene-bpSLO 001412	Lp34-2	gene	Pfam		protein matc	40	209	1.4E-58	+	.	Target=null 40 209;ID=match$2445_40_209;signature_desc=Lipoprotein;Name=PF01441;status=T;Dbxref="InterPro:IPR001800"
gene-bpSLO 001429	lp42	gene	Pfam		protein matc	39	205	9.9E-58	+	.	Target=null 39 205;ID=match$1133_39_205;signature_desc=Lipoprotein;Name=PF01441;status=T;Dbxref="InterPro:IPR001800"
gene-bpSLO 001434	lp42	gene	Pfam		protein matc	40	207	2.3E-69	+	.	Target=null 40 207;ID=match$1707_40_207;signature_desc=Lipoprotein;Name=PF01441;status=T;Dbxref="InterPro:IPR001800"
gene-bpSLO 001438	lp42	gene	Pfam		protein matc	40	208	1.3E-54	+	.	Target=null 40 208;ID=match$1082_40_208;signature_desc=Lipoprotein;Name=PF01441;status=T;Dbxref="InterPro:IPR001800"
gene-bpSLO 001475	lp58	gene	Pfam		protein matc	39	207	2.8E-64	+	.	Target=null 39 207;ID=match$1030_39_207;signature_desc=Lipoprotein;Name=PF01441;status=T;Dbxref="InterPro:IPR001800"
gene-bpSLO 001486	lp58	gene	Pfam		protein matc	39	206	7.7E-65	+	.	Target=null 39 206;ID=match$2188_39_206;signature_desc=Lipoprotein;Name=PF01441;status=T;Dbxref="InterPro:IPR001800"
gene-bpSLO 001512	lp58	gene	Pfam		protein matc	40	204	5.9E-51	+	.	Target=null 40 204;ID=match$803_40_204;signature_desc=Lipoprotein;Name=PF01441;status=T;Dbxref="InterPro:IPR001800"
gene-bpSLO 001530	lp60	gene	Pfam		protein matc	40	209	1.4E-58	+	.	Target=null 40 209;ID=match$2443_40_209;signature_desc=Lipoprotein;Name=PF01441;status=T;Dbxref="InterPro:IPR001800"
gene-bpSLO 001533	lp60	gene	Pfam		protein matc	39	205	3.9E-49	+	.	Target=null 39 205;ID=match$2012_39_205;signature_desc=Lipoprotein;Name=PF01441;status=T;Dbxref="InterPro:IPR001800"
gene-bpSLO 001538	lp60	gene	Pfam		protein matc	40	208	7.8E-62	+	.	Target=null 40 208;ID=match$2837_40_208;signature_desc=Lipoprotein;Name=PF01441;status=T;Dbxref="InterPro:IPR001800"
gene-bpSLO 001541	lp60	gene	Pfam		protein matc	40	205	4.4E-58	+	.	Target=null 40 205;ID=match$1305_40_205;signature_desc=Lipoprotein;Name=PF01441;status=T;Dbxref="InterPro:IPR001800"
gene-bpSLO 001546	lp60	gene	Pfam		protein matc	38	206	3.9E-64	+	.	Target=null 38 206;ID=match$1640_38_206;signature_desc=Lipoprotein;Name=PF01441;status=T;Dbxref="InterPro:IPR001800"
gene-bpSLO 001567	lp60	gene	Pfam		protein matc	23	192	6.1E-52	+	.	Target=null 23 192;ID=match$383_23_192;signature_desc=Lipoprotein;Name=PF01441;status=T;Dbxref="InterPro:IPR001800"
gene-bpSLO 001569	lp60	gene	Pfam		protein matc	39	206	2E-64	+	.	Target=null 39 206;ID=match$725_39_206;signature_desc=Lipoprotein;Name=PF01441;status=T;Dbxref="InterPro:IPR001800"
gene-bpSLO 001579	lp60	gene	Pfam		protein matc	39	211	1.6E-46	+	.	Target=null 39 211;ID=match$2817_39_211;signature_desc=Lipoprotein;Name=PF01441;status=T;Dbxref="InterPro:IPR001800"
gene-bpSLO 001585	lp60	gene	Pfam		protein matc	39	207	1.8E-54	+	.	Target=null 39 207;ID=match$1058_39_207;signature_desc=Lipoprotein;Name=PF01441;status=T;Dbxref="InterPro:IPR001800"
Vlp				Subfamily							
gene-bpSLO 001190	lp18	pseudo	Pfam	beta	protein matc	7	292	2.8E-46	+	.	Target=null 7 292;ID=match$1742_7_292;signature_desc=Borrelia lipoprotein;Name=PF00921;status=T;Dbxref="InterPro:IPR000680"
gene-bpSLO 001191	lp18	gene	Pfam	beta	protein matc	48	331	3.3E-45	+	.	Target=null 48 331;ID=match$1797_48_331;signature_desc=Borrelia lipoprotein;Name=PF00921;status=T;Dbxref="InterPro:IPR000680"
gene-bpSLO 001204	lp18	gene	Pfam	gamma	protein matc	1	278	4.2E-83	+	.	Target=null 1 278;ID=match$1146_1_278;signature_desc=Borrelia lipoprotein;Name=PF00921;status=T;Dbxref="InterPro:IPR000680"
gene-bpSLO 001209	lp24	gene	Pfam	alpha	protein matc	51	334	1.3E-64	+	.	Target=null 51 334;ID=match$2455_51_334;signature_desc=Borrelia lipoprotein;Name=PF00921;status=T;Dbxref="InterPro:IPR000680"
gene-bpSLO 001211	lp24	gene	Pfam	beta	protein matc	48	338	2.5E-48	+	.	Target=null 48 338;ID=match$2797_48_338;signature_desc=Borrelia lipoprotein;Name=PF00921;status=T;Dbxref="InterPro:IPR000680"
gene-bpSLO 001213	lp24	gene	Pfam	gamma	protein matc	34	318	2.6E-86	+	.	Target=null 34 318;ID=match$2297_34_318;signature_desc=Borrelia lipoprotein;Name=PF00921;status=T;Dbxref="InterPro:IPR000680"
gene-bpSLO 001214	lp24	gene	Pfam	delta	protein matc	45	325	1.9E-83	+	.	Target=null 45 325;ID=match$2027_45_325;signature_desc=Borrelia lipoprotein;Name=PF00921;status=T;Dbxref="InterPro:IPR000680"
gene-bpSLO 001215	lp24	gene	Pfam	delta	protein matc	34	317	1.9E-85	+	.	Target=null 34 317;ID=match$1323_34_317;signature_desc=Borrelia lipoprotein;Name=PF00921;status=T;Dbxref="InterPro:IPR000680"
gene-bpSLO 001216	lp24	gene	Pfam	delta	protein matc	44	332	1.5E-81	+	.	Target=null 44 332;ID=match$2626_44_332;signature_desc=Borrelia lipoprotein;Name=PF00921;status=T;Dbxref="InterPro:IPR000680"
gene-bpSLO 001217	lp24	gene	Pfam	delta	protein matc	44	329	1.6E-78	+	.	Target=null 44 329;ID=match$2972_44_329;signature_desc=Borrelia lipoprotein;Name=PF00921;status=T;Dbxref="InterPro:IPR000680"
gene-bpSLO 001218	lp24	gene	Pfam	gamma	protein matc	34	330	2.8E-86	+	.	Target=null 34 330;ID=match$1174_34_330;signature_desc=Borrelia lipoprotein;Name=PF00921;status=T;Dbxref="InterPro:IPR000680"
gene-bpSLO 001219	lp24	gene	Pfam	delta	protein matc	45	326	5E-83	+	.	Target=null 45 326;ID=match$995_45_326;signature_desc=Borrelia lipoprotein;Name=PF00921;status=T;Dbxref="InterPro:IPR000680"
gene-bpSLO 001220	lp24	gene	Pfam	beta	protein matc	20	309	1E-47	+	.	Target=null 20 309;ID=match$1177_20_309;signature_desc=Borrelia lipoprotein;Name=PF00921;status=T;Dbxref="InterPro:IPR000680"
gene-bpSLO 001221	lp24	gene	Pfam	alpha	protein matc	52	347	2.1E-71	+	.	Target=null 52 347;ID=match$1467_52_347;signature_desc=Borrelia lipoprotein;Name=PF00921;status=T;Dbxref="InterPro:IPR000680"
gene-bpSLO 001222	lp24	gene	Pfam	gamma	protein matc	1	55	2.9E-14	+	.	Target=null 1 55;ID=match$677_1_55;signature_desc=Borrelia lipoprotein;Name=PF00921;status=T;Dbxref="InterPro:IPR000680"
gene-bpSLO 001228	lp24	gene	Pfam	delta	protein matc	6	39	7.6E-08	+	.	Target=null 6 39;ID=match$575_6_39;signature_desc=Borrelia lipoprotein;Name=PF00921;status=T;Dbxref="InterPro:IPR000680"
gene-bpSLO 001233	lp28	gene	Pfam	alpha	protein matc	51	334	1.3E-64	+	.	Target=null 51 334;ID=match$2453_51_334;signature_desc=Borrelia lipoprotein;Name=PF00921;status=T;Dbxref="InterPro:IPR000680"
gene-bpSLO 001235	lp28	gene	Pfam	beta	protein matc	49	338	2E-46	+	.	Target=null 49 338;ID=match$2177_49_338;signature_desc=Borrelia lipoprotein;Name=PF00921;status=T;Dbxref="InterPro:IPR000680"
gene-bpSLO 001236	lp28	gene	Pfam	delta	protein matc	45	325	1.9E-84	+	.	Target=null 45 325;ID=match$1067_45_325;signature_desc=Borrelia lipoprotein;Name=PF00921;status=T;Dbxref="InterPro:IPR000680"
gene-bpSLO 001237	lp28	gene	Pfam	beta	protein matc	20	302	8.4E-45	+	.	Target=null 20 302;ID=match$1420_20_302;signature_desc=Borrelia lipoprotein;Name=PF00921;status=T;Dbxref="InterPro:IPR000680"
gene-bpSLO 001238	lp28	gene	Pfam	alpha	protein matc	54	349	3.1E-69	+	.	Target=null 54 349;ID=match$1422_54_349;signature_desc=Borrelia lipoprotein;Name=PF00921;status=T;Dbxref="InterPro:IPR000680"
gene-bpSLO 001239	lp28	gene	Pfam	beta	protein matc	49	337	2.2E-44	+	.	Target=null 49 337;ID=match$2187_49_337;signature_desc=Borrelia lipoprotein;Name=PF00921;status=T;Dbxref="InterPro:IPR000680"
gene-bpSLO 001240	lp28	gene	Pfam	delta	protein matc	44	333	1E-82	+	.	Target=null 44 333;ID=match$1953_44_333;signature_desc=Borrelia lipoprotein;Name=PF00921;status=T;Dbxref="InterPro:IPR000680"
gene-bpSLO 001243	lp28	gene	Pfam	delta	protein matc	38	322	4.1E-85	+	.	Target=null 38 322;ID=match$2128_38_322;signature_desc=Borrelia lipoprotein;Name=PF00921;status=T;Dbxref="InterPro:IPR000680"
gene-bpSLO 001246	lp28	gene	Pfam	gamma	protein matc	54	122	6.1E-26	+	.	Target=null 54 122;ID=match$2466_54_122;signature_desc=Borrelia lipoprotein;Name=PF00921;status=T;Dbxref="InterPro:IPR000680"
gene-bpSLO 001253	lp28	gene	Pfam	gamma	protein matc	1	28	1.1E-07	+	.	Target=null 1 28;ID=match$2517_1_28;signature_desc=Borrelia lipoprotein;Name=PF00921;status=T;Dbxref="InterPro:IPR000680"
gene-bpSLO 001254	lp28	gene	Pfam	gamma	protein matc	7	31	1.2E-07	+	.	Target=null 7 31;ID=match$359_7_31;signature_desc=Borrelia lipoprotein;Name=PF00921;status=T;Dbxref="InterPro:IPR000680"
gene-bpSLO 001257	lp30-1	gene	Pfam	alpha	protein matc	51	334	1.3E-64	+	.	Target=null 51 334;ID=match$2451_51_334;signature_desc=Borrelia lipoprotein;Name=PF00921;status=T;Dbxref="InterPro:IPR000680"
gene-bpSLO 001258	lp30-1	gene	Pfam	delta	protein matc	45	329	4.9E-86	+	.	Target=null 45 329;ID=match$2340_45_329;signature_desc=Borrelia lipoprotein;Name=PF00921;status=T;Dbxref="InterPro:IPR000680"
gene-bpSLO 001260	lp30-1	gene	Pfam	delta	protein matc	34	319	6.5E-86	+	.	Target=null 34 319;ID=match$674_34_319;signature_desc=Borrelia lipoprotein;Name=PF00921;status=T;Dbxref="InterPro:IPR000680"
gene-bpSLO 001261	lp30-1	gene	Pfam	gamma	protein matc	34	334	1.7E-79	+	.	Target=null 34 334;ID=match$694_34_334;signature_desc=Borrelia lipoprotein;Name=PF00921;status=T;Dbxref="InterPro:IPR000680"
gene-bpSLO 001282	lp30-1	gene	Pfam	beta	protein matc	20	303	5E-48	+	.	Target=null 20 303;ID=match$798_20_303;signature_desc=Borrelia lipoprotein;Name=PF00921;status=T;Dbxref="InterPro:IPR000680"
gene-bpSLO 001283	lp30-1	gene	Pfam	delta	protein matc	45	334	9E-82	+	.	Target=null 45 334;ID=match$2483_45_334;signature_desc=Borrelia lipoprotein;Name=PF00921;status=T;Dbxref="InterPro:IPR000680"
gene-bpSLO 001287	lp30-1	gene	Pfam	delta	protein matc	45	312	1.4E-79	+	.	Target=null 45 312;ID=match$2970_45_312;signature_desc=Borrelia lipoprotein;Name=PF00921;status=T;Dbxref="InterPro:IPR000680"
gene-bpSLO 001288	lp30-1	gene	Pfam	beta	protein matc	14	305	1.7E-50	+	.	Target=null 14 305;ID=match$2376_14_305;signature_desc=Borrelia lipoprotein;Name=PF00921;status=T;Dbxref="InterPro:IPR000680"
gene-bpSLO 001325	lp32	gene	Pfam	beta	protein matc	49	332	1.9E-45	+	.	Target=null 49 332;ID=match$2854_49_332;signature_desc=Borrelia lipoprotein;Name=PF00921;status=T;Dbxref="InterPro:IPR000680"
gene-bpSLO 001326	lp32	pseudo	Pfam	beta	protein matc	17	305	2.5E-50	+	.	Target=null 17 305;ID=match$2795_17_305;signature_desc=Borrelia lipoprotein;Name=PF00921;status=T;Dbxref="InterPro:IPR000680"
gene-bpSLO 001339	lp32	gene	Pfam	delta	protein matc	34	314	5.9E-87	+	.	Target=null 34 314;ID=match$2772_34_314;signature_desc=Borrelia lipoprotein;Name=PF00921;status=T;Dbxref="InterPro:IPR000680"
gene-bpSLO 001341	lp32	gene	Pfam	alpha	protein matc	43	338	8.5E-69	+	.	Target=null 43 338;ID=match$1136_43_338;signature_desc=Borrelia lipoprotein;Name=PF00921;status=T;Dbxref="InterPro:IPR000680"
gene-bpSLO 001342	lp32	gene	Pfam	beta	protein matc	20	306	9.9E-48	+	.	Target=null 20 306;ID=match$2370_20_306;signature_desc=Borrelia lipoprotein;Name=PF00921;status=T;Dbxref="InterPro:IPR000680"
gene-bpSLO 001343	lp32	gene	Pfam	gamma	protein matc	34	316	2.1E-87	+	.	Target=null 34 316;ID=match$3038_34_316;signature_desc=Borrelia lipoprotein;Name=PF00921;status=T;Dbxref="InterPro:IPR000680"
gene-bpSLO 001344	lp32	gene	Pfam	delta	protein matc	44	333	2.7E-80	+	.	Target=null 44 333;ID=match$1038_44_333;signature_desc=Borrelia lipoprotein;Name=PF00921;status=T;Dbxref="InterPro:IPR000680"
gene-bpSLO 001345	lp32	gene	Pfam	delta	protein matc	44	328	7.2E-87	+	.	Target=null 44 328;ID=match$1934_44_328;signature_desc=Borrelia lipoprotein;Name=PF00921;status=T;Dbxref="InterPro:IPR000680"
gene-bpSLO 001346	lp32	gene	Pfam	beta	protein matc	49	338	2.4E-46	+	.	Target=null 49 338;ID=match$439_49_338;signature_desc=Borrelia lipoprotein;Name=PF00921;status=T;Dbxref="InterPro:IPR000680"
gene-bpSLO 001347	lp32	gene	Pfam	delta	protein matc	45	326	7.9E-86	+	.	Target=null 45 326;ID=match$2405_45_326;signature_desc=Borrelia lipoprotein;Name=PF00921;status=T;Dbxref="InterPro:IPR000680"
gene-bpSLO 001348	lp32	gene	Pfam	gamma	protein matc	34	314	6.1E-92	+	.	Target=null 34 314;ID=match$369_34_314;signature_desc=Borrelia lipoprotein;Name=PF00921;status=T;Dbxref="InterPro:IPR000680"
gene-bpSLO 001351	lp32	gene	Pfam	gamma	protein matc	34	319	1.8E-84	+	.	Target=null 34 319;ID=match$301_34_319;signature_desc=Borrelia lipoprotein;Name=PF00921;status=T;Dbxref="InterPro:IPR000680"
gene-bpSLO 001352	lp32	gene	Pfam	alpha	protein matc	51	336	6E-65	+	.	Target=null 51 336;ID=match$2424_51_336;signature_desc=Borrelia lipoprotein;Name=PF00921;status=T;Dbxref="InterPro:IPR000680"
gene-bpSLO 001357	lp34-1	gene	Pfam	gamma	protein matc	14	301	6.2E-48	+	.	Target=null 14 301;ID=match$1018_14_301;signature_desc=Borrelia lipoprotein;Name=PF00921;status=T;Dbxref="InterPro:IPR000680"
gene-bpSLO 001358	lp34-1	gene	Pfam	alpha	protein matc	43	340	2.1E-59	+	.	Target=null 43 340;ID=match$2890_43_340;signature_desc=Borrelia lipoprotein;Name=PF00921;status=T;Dbxref="InterPro:IPR000680"
gene-bpSLO 001359	lp34-1	gene	Pfam	gamma	protein matc	178	268	2.1E-27	+	.	Target=null 178 268;ID=match$678_178_268;signature_desc=Borrelia lipoprotein;Name=PF00921;status=T;Dbxref="InterPro:IPR000680"
gene-bpSLO 001359	lp34-1	gene	Pfam		protein matc	28	198	7E-47	+	.	Target=null 28 198;ID=match$678_28_198;signature_desc=Borrelia lipoprotein;Name=PF00921;status=T;Dbxref="InterPro:IPR000680"
gene-bpSLO 001372	lp34-1	gene	Pfam	gamma	protein matc	1	273	2.3E-79	+	.	Target=null 1 273;ID=match$152_1_273;signature_desc=Borrelia lipoprotein;Name=PF00921;status=T;Dbxref="InterPro:IPR000680"
gene-bpSLO 001373	lp34-1	gene	Pfam	delta	protein matc	44	321	3.1E-86	+	.	Target=null 44 321;ID=match$1334_44_321;signature_desc=Borrelia lipoprotein;Name=PF00921;status=T;Dbxref="InterPro:IPR000680"
gene-bpSLO 001375	lp34-1	gene	Pfam	gamma	protein matc	7	287	2.6E-79	+	.	Target=null 7 287;ID=match$137_7_287;signature_desc=Borrelia lipoprotein;Name=PF00921;status=T;Dbxref="InterPro:IPR000680"
gene-bpSLO 001376	lp34-1	gene	Pfam	alpha	protein matc	55	354	5.6E-70	+	.	Target=null 55 354;ID=match$1529_55_354;signature_desc=Borrelia lipoprotein;Name=PF00921;status=T;Dbxref="InterPro:IPR000680"
gene-bpSLO 001377	lp34-1	gene	Pfam	beta	protein matc	14	301	1.2E-44	+	.	Target=null 14 301;ID=match$17_14_301;signature_desc=Borrelia lipoprotein;Name=PF00921;status=T;Dbxref="InterPro:IPR000680"
gene-bpSLO 001379	lp34-1	gene	Pfam	gamma	protein matc	1	278	5.6E-82	+	.	Target=null 1 278;ID=match$176_1_278;signature_desc=Borrelia lipoprotein;Name=PF00921;status=T;Dbxref="InterPro:IPR000680"
gene-bpSLO 001380	lp34-1	gene	Pfam	alpha	protein matc	45	341	1.8E-73	+	.	Target=null 45 341;ID=match$242_45_341;signature_desc=Borrelia lipoprotein;Name=PF00921;status=T;Dbxref="InterPro:IPR000680"
gene-bpSLO 001381	lp34-1	gene	Pfam	beta	protein matc	20	305	5.7E-46	+	.	Target=null 20 305;ID=match$2734_20_305;signature_desc=Borrelia lipoprotein;Name=PF00921;status=T;Dbxref="InterPro:IPR000680"
gene-bpSLO 001382	lp34-1	gene	Pfam	delta	protein matc	45	335	1.6E-84	+	.	Target=null 45 335;ID=match$1711_45_335;signature_desc=Borrelia lipoprotein;Name=PF00921;status=T;Dbxref="InterPro:IPR000680"
gene-bpSLO 001384	lp34-1	gene	Pfam	gamma	protein matc	34	319	4.8E-93	+	.	Target=null 34 319;ID=match$1340_34_319;signature_desc=Borrelia lipoprotein;Name=PF00921;status=T;Dbxref="InterPro:IPR000680"
gene-bpSLO 001385	lp34-1	gene	Pfam	delta	protein matc	45	333	1.6E-86	+	.	Target=null 45 333;ID=match$1559_45_333;signature_desc=Borrelia lipoprotein;Name=PF00921;status=T;Dbxref="InterPro:IPR000680"
gene-bpSLO 001386	lp34-1	gene	Pfam	alpha	protein matc	55	338	4.9E-63	+	.	Target=null 55 338;ID=match$2802_55_338;signature_desc=Borrelia lipoprotein;Name=PF00921;status=T;Dbxref="InterPro:IPR000680"
gene-bpSLO 001389	lp34-2	gene	Pfam	alpha	protein matc	5	191	8.7E-43	+	.	Target=null 5 191;ID=match$1254_5_191;signature_desc=Borrelia lipoprotein;Name=PF00921;status=T;Dbxref="InterPro:IPR000680"
gene-bpSLO 001413	lp34-2	gene	Pfam	gamma	protein matc	178	268	2.1E-27	+	.	Target=null 178 268;ID=match$680_178_268;signature_desc=Borrelia lipoprotein;Name=PF00921;status=T;Dbxref="InterPro:IPR000680"
gene-bpSLO 001413	lp34-2	gene	Pfam		protein matc	28	198	7E-47	+	.	Target=null 28 198;ID=match$680_28_198;signature_desc=Borrelia lipoprotein;Name=PF00921;status=T;Dbxref="InterPro:IPR000680"
gene-bpSLO 001414	lp34-2	gene	Pfam	alpha	protein matc	43	340	2.1E-59	+	.	Target=null 43 340;ID=match$2886_43_340;signature_desc=Borrelia lipoprotein;Name=PF00921;status=T;Dbxref="InterPro:IPR000680"
gene-bpSLO 001415	lp34-2	gene	Pfam	beta	protein matc	14	301	6.2E-48	+	.	Target=null 14 301;ID=match$1020_14_301;signature_desc=Borrelia lipoprotein;Name=PF00921;status=T;Dbxref="InterPro:IPR000680"
gene-bpSLO 001420	lp42	gene	Pfam	alpha	protein matc	51	336	6E-65	+	.	Target=null 51 336;ID=match$2422_51_336;signature_desc=Borrelia lipoprotein;Name=PF00921;status=T;Dbxref="InterPro:IPR000680"
gene-bpSLO 001421	lp42	gene	Pfam	gamma	protein matc	34	319	1.8E-84	+	.	Target=null 34 319;ID=match$303_34_319;signature_desc=Borrelia lipoprotein;Name=PF00921;status=T;Dbxref="InterPro:IPR000680"
gene-bpSLO 001424	lp42	gene	Pfam	gamma	protein matc	34	314	6.1E-92	+	.	Target=null 34 314;ID=match$365_34_314;signature_desc=Borrelia lipoprotein;Name=PF00921;status=T;Dbxref="InterPro:IPR000680"
gene-bpSLO 001425	lp42	gene	Pfam	delta	protein matc	45	326	7.9E-86	+	.	Target=null 45 326;ID=match$2407_45_326;signature_desc=Borrelia lipoprotein;Name=PF00921;status=T;Dbxref="InterPro:IPR000680"
gene-bpSLO 001426	lp42	gene	Pfam	beta	protein matc	49	338	2.4E-46	+	.	Target=null 49 338;ID=match$441_49_338;signature_desc=Borrelia lipoprotein;Name=PF00921;status=T;Dbxref="InterPro:IPR000680"
gene-bpSLO 001427	lp42	gene	Pfam	delta	protein matc	44	323	1.4E-87	+	.	Target=null 44 323;ID=match$2512_44_323;signature_desc=Borrelia lipoprotein;Name=PF00921;status=T;Dbxref="InterPro:IPR000680"
gene-bpSLO 001428	lp42	gene	Pfam	delta	protein matc	8	291	2.1E-83	+	.	Target=null 8 291;ID=match$2224_8_291;signature_desc=Borrelia lipoprotein;Name=PF00921;status=T;Dbxref="InterPro:IPR000680"
gene-bpSLO 001430	lp42	gene	Pfam	delta	protein matc	34	324	7E-85	+	.	Target=null 34 324;ID=match$1519_34_324;signature_desc=Borrelia lipoprotein;Name=PF00921;status=T;Dbxref="InterPro:IPR000680"
gene-bpSLO 001431	lp42	gene	Pfam	beta	protein matc	20	310	1.8E-49	+	.	Target=null 20 310;ID=match$2500_20_310;signature_desc=Borrelia lipoprotein;Name=PF00921;status=T;Dbxref="InterPro:IPR000680"
gene-bpSLO 001432	lp42	gene	Pfam	alpha	protein matc	7	304	1.2E-72	+	.	Target=null 7 304;ID=match$380_7_304;signature_desc=Borrelia lipoprotein;Name=PF00921;status=T;Dbxref="InterPro:IPR000680"
gene-bpSLO 001433	lp42	pseudo	Pfam	delta	protein matc	19	62	1.2E-13	+	.	Target=null 19 62;ID=match$2218_19_62;signature_desc=Borrelia lipoprotein;Name=PF00921;status=T;Dbxref="InterPro:IPR000680"
gene-bpSLO 001435	lp42	gene	Pfam	delta	protein matc	34	319	3.3E-89	+	.	Target=null 34 319;ID=match$200_34_319;signature_desc=Borrelia lipoprotein;Name=PF00921;status=T;Dbxref="InterPro:IPR000680"
gene-bpSLO 001437	lp42	gene	Pfam	delta	protein matc	8	144	1.7E-37	+	.	Target=null 8 144;ID=match$2793_8_144;signature_desc=Borrelia lipoprotein;Name=PF00921;status=T;Dbxref="InterPro:IPR000680"
gene-bpSLO 001439	lp42	gene	Pfam	gamma	protein matc	11	50	1.5E-10	+	.	Target=null 11 50;ID=match$1894_11_50;signature_desc=Borrelia lipoprotein;Name=PF00921;status=T;Dbxref="InterPro:IPR000680"
gene-bpSLO 001445	lp42	gene	Pfam	beta	protein matc	49	335	5.8E-51	+	.	Target=null 49 335;ID=match$2640_49_335;signature_desc=Borrelia lipoprotein;Name=PF00921;status=T;Dbxref="InterPro:IPR000680"
gene-bpSLO 001448	lp42	pseudo	Pfam	beta	protein matc	48	187	1.5E-22	+	.	Target=null 48 187;ID=match$2645_48_187;signature_desc=Borrelia lipoprotein;Name=PF00921;status=T;Dbxref="InterPro:IPR000680"
gene-bpSLO 001448	lp42	pseudo	Pfam		protein matc	22	137	1.4E-19	+	.	Target=null 22 137;ID=match$1819_22_137;signature_desc=Borrelia lipoprotein;Name=PF00921;status=T;Dbxref="InterPro:IPR000680"
gene-bpSLO 001450	lp42	pseudo	Pfam	gamma	protein matc	124	197	1.4E-17	+	.	Target=null 124 197;ID=match$1052_124_197;signature_desc=Borrelia lipoprotein;Name=PF00921;status=T;Dbxref="InterPro:IPR000680"
gene-bpSLO 001450	lp42	pseudo	Pfam		protein matc	34	132	2.7E-30	+	.	Target=null 34 132;ID=match$1052_34_132;signature_desc=Borrelia lipoprotein;Name=PF00921;status=T;Dbxref="InterPro:IPR000680"
gene-bpSLO 001462	lp58	gene	Pfam	alpha	protein matc	51	336	6E-65	+	.	Target=null 51 336;ID=match$2420_51_336;signature_desc=Borrelia lipoprotein;Name=PF00921;status=T;Dbxref="InterPro:IPR000680"
gene-bpSLO 001463	lp58	gene	Pfam	gamma	protein matc	34	319	1.8E-84	+	.	Target=null 34 319;ID=match$299_34_319;signature_desc=Borrelia lipoprotein;Name=PF00921;status=T;Dbxref="InterPro:IPR000680"
gene-bpSLO 001466	lp58	gene	Pfam	gamma	protein matc	34	314	6.1E-92	+	.	Target=null 34 314;ID=match$367_34_314;signature_desc=Borrelia lipoprotein;Name=PF00921;status=T;Dbxref="InterPro:IPR000680"
gene-bpSLO 001467	lp58	gene	Pfam	delta	protein matc	45	326	7.9E-86	+	.	Target=null 45 326;ID=match$2409_45_326;signature_desc=Borrelia lipoprotein;Name=PF00921;status=T;Dbxref="InterPro:IPR000680"
gene-bpSLO 001468	lp58	gene	Pfam	beta	protein matc	49	338	2.4E-46	+	.	Target=null 49 338;ID=match$443_49_338;signature_desc=Borrelia lipoprotein;Name=PF00921;status=T;Dbxref="InterPro:IPR000680"
gene-bpSLO 001469	lp58	gene	Pfam	delta	protein matc	44	323	1.4E-87	+	.	Target=null 44 323;ID=match$2514_44_323;signature_desc=Borrelia lipoprotein;Name=PF00921;status=T;Dbxref="InterPro:IPR000680"
gene-bpSLO 001470	lp58	gene	Pfam	delta	protein matc	45	331	2.1E-83	+	.	Target=null 45 331;ID=match$822_45_331;signature_desc=Borrelia lipoprotein;Name=PF00921;status=T;Dbxref="InterPro:IPR000680"
gene-bpSLO 001474	lp58	gene	Pfam	delta	protein matc	38	159	1.3E-34	+	.	Target=null 38 159;ID=match$1845_38_159;signature_desc=Borrelia lipoprotein;Name=PF00921;status=T;Dbxref="InterPro:IPR000680"
gene-bpSLO 001476	lp58	gene	Pfam	beta	protein matc	29	318	4.9E-46	+	.	Target=null 29 318;ID=match$943_29_318;signature_desc=Borrelia lipoprotein;Name=PF00921;status=T;Dbxref="InterPro:IPR000680"
gene-bpSLO 001477	lp58	gene	Pfam	alpha	protein matc	7	303	3.6E-71	+	.	Target=null 7 303;ID=match$522_7_303;signature_desc=Borrelia lipoprotein;Name=PF00921;status=T;Dbxref="InterPro:IPR000680"
gene-bpSLO 001478	lp58	gene	Pfam	gamma	protein matc	34	325	4.6E-87	+	.	Target=null 34 325;ID=match$834_34_325;signature_desc=Borrelia lipoprotein;Name=PF00921;status=T;Dbxref="InterPro:IPR000680"
gene-bpSLO 001479	lp58	gene	Pfam	delta	protein matc	44	334	8.7E-86	+	.	Target=null 44 334;ID=match$2022_44_334;signature_desc=Borrelia lipoprotein;Name=PF00921;status=T;Dbxref="InterPro:IPR000680"
gene-bpSLO 001482	lp58	gene	Pfam	delta	protein matc	34	315	6.2E-88	+	.	Target=null 34 315;ID=match$3003_34_315;signature_desc=Borrelia lipoprotein;Name=PF00921;status=T;Dbxref="InterPro:IPR000680"
gene-bpSLO 001484	lp58	pseudo	Pfam	beta	protein matc	3	171	2.2E-15	+	.	Target=null 3 171;ID=match$88_3_171;signature_desc=Borrelia lipoprotein;Name=PF00921;status=T;Dbxref="InterPro:IPR000680"
gene-bpSLO 001484	lp58	pseudo	Pfam		protein matc	165	212	7.7E-09	+	.	Target=null 165 212;ID=match$88_165_212;signature_desc=Borrelia lipoprotein;Name=PF00921;status=T;Dbxref="InterPro:IPR000680"
gene-bpSLO 001485	lp58	gene	Pfam	gamma	protein matc	117	246	3.4E-35	+	.	Target=null 117 246;ID=match$2893_117_246;signature_desc=Borrelia lipoprotein;Name=PF00921;status=T;Dbxref="InterPro:IPR000680"
gene-bpSLO 001485	lp58	gene	Pfam		protein matc	28	119	1.8E-33	+	.	Target=null 28 119;ID=match$2893_28_119;signature_desc=Borrelia lipoprotein;Name=PF00921;status=T;Dbxref="InterPro:IPR000680"
gene-bpSLO 001489	lp58	gene	Pfam	gamma	protein matc	7	173	1.8E-44	+	.	Target=null 7 173;ID=match$2150_7_173;signature_desc=Borrelia lipoprotein;Name=PF00921;status=T;Dbxref="InterPro:IPR000680"
gene-bpSLO 001507	lp58	gene	Pfam	delta	protein matc	45	244	5.4E-51	+	.	Target=null 45 244;ID=match$390_45_244;signature_desc=Borrelia lipoprotein;Name=PF00921;status=T;Dbxref="InterPro:IPR000680"
gene-bpSLO 001507	lp58	gene	Pfam		protein matc	239	280	4.3E-10	+	.	Target=null 239 280;ID=match$390_239_280;signature_desc=Borrelia lipoprotein;Name=PF00921;status=T;Dbxref="InterPro:IPR000680"
gene-bpSLO 001510	lp58	gene	Pfam	delta	protein matc	45	329	2.8E-85	+	.	Target=null 45 329;ID=match$1092_45_329;signature_desc=Borrelia lipoprotein;Name=PF00921;status=T;Dbxref="InterPro:IPR000680"
gene-bpSLO 001513	lp58	pseudo	Pfam	gamma	protein matc	43	165	6.7E-39	+	.	Target=null 43 165;ID=match$2098_43_165;signature_desc=Borrelia lipoprotein;Name=PF00921;status=T;Dbxref="InterPro:IPR000680"
gene-bpSLO 001513	lp58	pseudo	Pfam		protein matc	4	166	6.7E-47	+	.	Target=null 4 166;ID=match$1537_4_166;signature_desc=Borrelia lipoprotein;Name=PF00921;status=T;Dbxref="InterPro:IPR000680"
gene-bpSLO 001516	lp58	gene	Pfam	alpha	protein matc	57	357	3.1E-69	+	.	Target=null 57 357;ID=match$3196_57_357;signature_desc=Borrelia lipoprotein;Name=PF00921;status=T;Dbxref="InterPro:IPR000680"
gene-bpSLO 001517	lp58	pseudo	Pfam	beta	protein matc	14	154	1.1E-18	+	.	Target=null 14 154;ID=match$2502_14_154;signature_desc=Borrelia lipoprotein;Name=PF00921;status=T;Dbxref="InterPro:IPR000680"
gene-bpSLO 001518	lp58	gene	Pfam	delta	protein matc	34	320	1.4E-88	+	.	Target=null 34 320;ID=match$2786_34_320;signature_desc=Borrelia lipoprotein;Name=PF00921;status=T;Dbxref="InterPro:IPR000680"
gene-bpSLO 001520	lp58	gene	Pfam	gamma	protein matc	34	319	4.8E-93	+	.	Target=null 34 319;ID=match$1342_34_319;signature_desc=Borrelia lipoprotein;Name=PF00921;status=T;Dbxref="InterPro:IPR000680"
gene-bpSLO 001521	lp58	gene	Pfam	delta	protein matc	44	332	1.8E-86	+	.	Target=null 44 332;ID=match$655_44_332;signature_desc=Borrelia lipoprotein;Name=PF00921;status=T;Dbxref="InterPro:IPR000680"
gene-bpSLO 001522	lp58	gene	Pfam	alpha	protein matc	55	338	4.9E-63	+	.	Target=null 55 338;ID=match$2800_55_338;signature_desc=Borrelia lipoprotein;Name=PF00921;status=T;Dbxref="InterPro:IPR000680"
gene-bpSLO 001527	lp60	gene	Pfam	beta	protein matc	14	301	6.2E-48	+	.	Target=null 14 301;ID=match$1016_14_301;signature_desc=Borrelia lipoprotein;Name=PF00921;status=T;Dbxref="InterPro:IPR000680"
gene-bpSLO 001528	lp60	gene	Pfam	alpha	protein matc	43	340	2.1E-59	+	.	Target=null 43 340;ID=match$2888_43_340;signature_desc=Borrelia lipoprotein;Name=PF00921;status=T;Dbxref="InterPro:IPR000680"
gene-bpSLO 001529	lp60	gene	Pfam	gamma	protein matc	178	268	2.1E-27	+	.	Target=null 178 268;ID=match$682_178_268;signature_desc=Borrelia lipoprotein;Name=PF00921;status=T;Dbxref="InterPro:IPR000680"
gene-bpSLO 001529	lp60	gene	Pfam		protein matc	28	198	7E-47	+	.	Target=null 28 198;ID=match$682_28_198;signature_desc=Borrelia lipoprotein;Name=PF00921;status=T;Dbxref="InterPro:IPR000680"
gene-bpSLO 001531	lp60	gene	Pfam	gamma	protein matc	44	319	2.6E-86	+	.	Target=null 44 319;ID=match$107_44_319;signature_desc=Borrelia lipoprotein;Name=PF00921;status=T;Dbxref="InterPro:IPR000680"
gene-bpSLO 001532	lp60	gene	Pfam	delta	protein matc	74	358	5E-86	+	.	Target=null 74 358;ID=match$2869_74_358;signature_desc=Borrelia lipoprotein;Name=PF00921;status=T;Dbxref="InterPro:IPR000680"
gene-bpSLO 001534	lp60	gene	Pfam	beta	protein matc	115	244	4.9E-24	+	.	Target=null 115 244;ID=match$3055_115_244;signature_desc=Borrelia lipoprotein;Name=PF00921;status=T;Dbxref="InterPro:IPR000680"
gene-bpSLO 001534	lp60	gene	Pfam		protein matc	14	110	2.3E-10	+	.	Target=null 14 110;ID=match$3055_14_110;signature_desc=Borrelia lipoprotein;Name=PF00921;status=T;Dbxref="InterPro:IPR000680"
gene-bpSLO 001535	lp60	gene	Pfam	alpha	protein matc	55	354	1.3E-71	+	.	Target=null 55 354;ID=match$2268_55_354;signature_desc=Borrelia lipoprotein;Name=PF00921;status=T;Dbxref="InterPro:IPR000680"
gene-bpSLO 001537	lp60	gene	Pfam	gamma	protein matc	1	284	4.7E-85	+	.	Target=null 1 284;ID=match$191_1_284;signature_desc=Borrelia lipoprotein;Name=PF00921;status=T;Dbxref="InterPro:IPR000680"
gene-bpSLO 001540	lp60	gene	Pfam	gamma	protein matc	1	279	2E-83	+	.	Target=null 1 279;ID=match$1281_1_279;signature_desc=Borrelia lipoprotein;Name=PF00921;status=T;Dbxref="InterPro:IPR000680"
gene-bpSLO 001544	lp60	gene	Pfam	delta	protein matc	45	324	1.1E-84	+	.	Target=null 45 324;ID=match$2619_45_324;signature_desc=Borrelia lipoprotein;Name=PF00921;status=T;Dbxref="InterPro:IPR000680"
gene-bpSLO 001545	lp60	pseudo	Pfam	delta	protein matc	13	88	1.6E-22	+	.	Target=null 13 88;ID=match$1850_13_88;signature_desc=Borrelia lipoprotein;Name=PF00921;status=T;Dbxref="InterPro:IPR000680"
gene-bpSLO 001545	lp60	pseudo	Pfam		protein matc	12	72	1.1E-08	+	.	Target=null 12 72;ID=match$2815_12_72;signature_desc=Borrelia lipoprotein;Name=PF00921;status=T;Dbxref="InterPro:IPR000680"
gene-bpSLO 001545	lp60	pseudo	Pfam		protein matc	74	141	1.4E-28	+	.	Target=null 74 141;ID=match$2815_74_141;signature_desc=Borrelia lipoprotein;Name=PF00921;status=T;Dbxref="InterPro:IPR000680"
gene-bpSLO 001568	lp60	gene	Pfam	delta	protein matc	29	313	6.3E-80	+	.	Target=null 29 313;ID=match$1862_29_313;signature_desc=Borrelia lipoprotein;Name=PF00921;status=T;Dbxref="InterPro:IPR000680"
gene-bpSLO 001570	lp60	gene	Pfam	alpha	protein matc	54	350	6.4E-68	+	.	Target=null 54 350;ID=match$760_54_350;signature_desc=Borrelia lipoprotein;Name=PF00921;status=T;Dbxref="InterPro:IPR000680"
gene-bpSLO 001571	lp60	pseudo	Pfam	beta	protein matc	20	270	6.3E-31	+	.	Target=null 20 270;ID=match$2951_20_270;signature_desc=Borrelia lipoprotein;Name=PF00921;status=T;Dbxref="InterPro:IPR000680"
gene-bpSLO 001571	lp60	pseudo	Pfam		protein matc	12	50	4.4E-11	+	.	Target=null 12 50;ID=match$1553_12_50;signature_desc=Borrelia lipoprotein;Name=PF00921;status=T;Dbxref="InterPro:IPR000680"
gene-bpSLO 001572	lp60	gene	Pfam	gamma	protein matc	35	313	1.7E-86	+	.	Target=null 35 313;ID=match$695_35_313;signature_desc=Borrelia lipoprotein;Name=PF00921;status=T;Dbxref="InterPro:IPR000680"
gene-bpSLO 001573	lp60	pseudo	Pfam	delta	protein matc	44	250	6.9E-53	+	.	Target=null 44 250;ID=match$1054_44_250;signature_desc=Borrelia lipoprotein;Name=PF00921;status=T;Dbxref="InterPro:IPR000680"
gene-bpSLO 001573	lp60	pseudo	Pfam		protein matc	5	77	5.5E-28	+	.	Target=null 5 77;ID=match$2068_5_77;signature_desc=Borrelia lipoprotein;Name=PF00921;status=T;Dbxref="InterPro:IPR000680"
gene-bpSLO 001574	lp60	gene	Pfam	delta	protein matc	45	324	7.8E-82	+	.	Target=null 45 324;ID=match$612_45_324;signature_desc=Borrelia lipoprotein;Name=PF00921;status=T;Dbxref="InterPro:IPR000680"
gene-bpSLO 001575	lp60	gene	Pfam	gamma	protein matc	34	319	1.2E-84	+	.	Target=null 34 319;ID=match$2417_34_319;signature_desc=Borrelia lipoprotein;Name=PF00921;status=T;Dbxref="InterPro:IPR000680"
gene-bpSLO 001578	lp60	gene	Pfam	delta	protein matc	44	330	2.3E-87	+	.	Target=null 44 330;ID=match$567_44_330;signature_desc=Borrelia lipoprotein;Name=PF00921;status=T;Dbxref="InterPro:IPR000680"
gene-bpSLO 001580	lp60	gene	Pfam	gamma	protein matc	43	335	1.9E-91	+	.	Target=null 43 335;ID=match$2282_43_335;signature_desc=Borrelia lipoprotein;Name=PF00921;status=T;Dbxref="InterPro:IPR000680"
gene-bpSLO 001584	lp60	gene	Pfam	delta	protein matc	44	330	1.9E-88	+	.	Target=null 44 330;ID=match$1111_44_330;signature_desc=Borrelia lipoprotein;Name=PF00921;status=T;Dbxref="InterPro:IPR000680"
gene-bpSLO 001586	lp60	pseudo	Pfam	gamma	protein matc	36	73	3.8E-13	+	.	Target=null 36 73;ID=match$437_36_73;signature_desc=Borrelia lipoprotein;Name=PF00921;status=T;Dbxref="InterPro:IPR000680"
gene-bpSLO 001586	lp60	pseudo	Pfam		protein matc	2	245	7.9E-67	+	.	Target=null 2 245;ID=match$820_2_245;signature_desc=Borrelia lipoprotein;Name=PF00921;status=T;Dbxref="InterPro:IPR000680"
gene-bpSLO 001587	lp60	gene	Pfam	delta	protein matc	44	323	1.9E-85	+	.	Target=null 44 323;ID=match$1431_44_323;signature_desc=Borrelia lipoprotein;Name=PF00921;status=T;Dbxref="InterPro:IPR000680"
gene-bpSLO 001590	lp60	gene	Pfam	beta	protein matc	48	334	2.7E-51	+	.	Target=null 48 334;ID=match$762_48_334;signature_desc=Borrelia lipoprotein;Name=PF00921;status=T;Dbxref="InterPro:IPR000680"

Vsp	replicon	gene/pseudo									
gene-bvRMA01 001093	lp25	gene		Pfam	protein matc	39	210	1.2E-46	+	.	Target=null 39 210;ID=match$653_39_210;signature_desc=Lipoprotein;Name=PF01441;status=T;Dbxref="InterPro:IPR001800"
gene-bvRMA01 001109	lp30	gene		Pfam	protein matc	39	209	2E-58	+	.	Target=null 39 209;ID=match$1736_39_209;signature_desc=Lipoprotein;Name=PF01441;status=T;Dbxref="InterPro:IPR001800"
gene-bvRMA01 001121	lp30	gene		Pfam	protein matc	38	204	1E-62	+	.	Target=null 38 204;ID=match$1966_38_204;signature_desc=Lipoprotein;Name=PF01441;status=T;Dbxref="InterPro:IPR001800"
gene-bvRMA01 001128	lp30	gene		Pfam	protein matc	39	208	1.7E-65	+	.	Target=null 39 208;ID=match$2240_39_208;signature_desc=Lipoprotein;Name=PF01441;status=T;Dbxref="InterPro:IPR001800"
gene-bvRMA01 001142	lp31	gene		Pfam	protein matc	39	206	8.2E-60	+	.	Target=null 39 206;ID=match$114_39_206;signature_desc=Lipoprotein;Name=PF01441;status=T;Dbxref="InterPro:IPR001800"
gene-bvRMA01 001144	lp31	gene		Pfam	protein matc	39	207	3.1E-53	+	.	Target=null 39 207;ID=match$218_39_207;signature_desc=Lipoprotein;Name=PF01441;status=T;Dbxref="InterPro:IPR001800"
gene-bvRMA01 001160	lp31	gene		Pfam	protein matc	39	206	7.7E-54	+	.	Target=null 39 206;ID=match$2686_39_206;signature_desc=Lipoprotein;Name=PF01441;status=T;Dbxref="InterPro:IPR001800"
gene-bvRMA01 001196	lp35	gene		Pfam	protein matc	39	209	2E-58	+	.	Target=null 39 209;ID=match$1734_39_209;signature_desc=Lipoprotein;Name=PF01441;status=T;Dbxref="InterPro:IPR001800"
gene-bvRMA01 001228	lp37	pseudo		Pfam	protein matc	38	117	5.1E-35	+	.	Target=null 38 117;ID=match$613_38_117;signature_desc=Lipoprotein;Name=PF01441;status=T;Dbxref="InterPro:IPR001800"
gene-bvRMA01 001228	lp37	pseudo		Pfam	protein matc	3	94	1.3E-27	+	.	Target=null 3 94;ID=match$1272_3_94;signature_desc=Lipoprotein;Name=PF01441;status=T;Dbxref="InterPro:IPR001800"

Vlp			Subfamily								
gene-bvRMA01 001081	lp17-2	gene	delta	Pfam	protein matc	34	316	1E-86	+	.	Target=null 34 316;ID=match$2672_34_316;signature_desc=Borrelia lipoprotein;Name=PF00921;status=T;Dbxref="InterPro:IPR000680"
gene-bvRMA01 001087	lp25	gene	alpha	Pfam	protein matc	52	343	2E-70	+	.	Target=null 52 343;ID=match$999_52_343;signature_desc=Borrelia lipoprotein;Name=PF00921;status=T;Dbxref="InterPro:IPR000680"
gene-bvRMA01 001088	lp25	gene	gamma	Pfam	protein matc	34	309	2E-86	+	.	Target=null 34 309;ID=match$335_34_309;signature_desc=Borrelia lipoprotein;Name=PF00921;status=T;Dbxref="InterPro:IPR000680"
gene-bvRMA01 001089	lp25	gene	delta	Pfam	protein matc	39	163	1.1E-40	+	.	Target=null 39 163;ID=match$2251_39_163;signature_desc=Borrelia lipoprotein;Name=PF00921;status=T;Dbxref="InterPro:IPR000680"
gene-bvRMA01 001090	lp25	gene	alpha	Pfam	protein matc	52	344	5.4E-69	+	.	Target=null 52 344;ID=match$295_52_344;signature_desc=Borrelia lipoprotein;Name=PF00921;status=T;Dbxref="InterPro:IPR000680"
gene-bvRMA01 001092	lp25	gene	delta	Pfam	protein matc	48	325	2.9E-89	+	.	Target=null 48 325;ID=match$1979_48_325;signature_desc=Borrelia lipoprotein;Name=PF00921;status=T;Dbxref="InterPro:IPR000680"
gene-bvRMA01 001096	lp25	gene	delta	Pfam	protein matc	9	61	2.8E-18	+	.	Target=null 9 61;ID=match$2624_9_61;signature_desc=Borrelia lipoprotein;Name=PF00921;status=T;Dbxref="InterPro:IPR000680"
gene-bvRMA01 001102	lp25	gene	gamma	Pfam	protein matc	6	42	0.0000015	+	.	Target=null 6 42;ID=match$367_6_42;signature_desc=Borrelia lipoprotein;Name=PF00921;status=T;Dbxref="InterPro:IPR000680"
gene-bvRMA01 001107	lp30	gene	beta	Pfam	protein matc	45	326	7.5E-45	+	.	Target=null 45 326;ID=match$2712_45_326;signature_desc=Borrelia lipoprotein;Name=PF00921;status=T;Dbxref="InterPro:IPR000680"
gene-bvRMA01 001108	lp30	gene	gamma	Pfam	protein matc	44	319	1.8E-85	+	.	Target=null 44 319;ID=match$8_44_319;signature_desc=Borrelia lipoprotein;Name=PF00921;status=T;Dbxref="InterPro:IPR000680"
gene-bvRMA01_001110	lp30	gene	gamma	Pfam	protein_matc	34	312	4.9E-90	+	.	Target=null 34 312;ID=match$1078_34_312;signature_desc=Borrelia lipoprotein;Name=PF00921;status=T;Dbxref="InterPro:IPR000680"
gene-bvRMA01 001120	lp30	gene	delta	Pfam	protein matc	45	330	5.5E-88	+	.	Target=null 45 330;ID=match$2261_45_330;signature_desc=Borrelia lipoprotein;Name=PF00921;status=T;Dbxref="InterPro:IPR000680"
gene-bvRMA01 001122	lp30	pseudo	alpha	Pfam	protein matc	7	185	1.4E-32	+	.	Target=null 7 185;ID=match$1297_7_185;signature_desc=Borrelia lipoprotein;Name=PF00921;status=T;Dbxref="InterPro:IPR000680"
gene-bvRMA01 001122	lp30	pseudo		Pfam	protein matc	1	117	3E-33	+	.	Target=null 1 117;ID=match$2150_1_117;signature_desc=Borrelia lipoprotein;Name=PF00921;status=T;Dbxref="InterPro:IPR000680"
gene-bvRMA01 001125	lp30	pseudo	beta	Pfam	protein matc	51	100	2.3E-08	+	.	Target=null 51 100;ID=match$2674_51_100;signature_desc=Borrelia lipoprotein;Name=PF00921;status=T;Dbxref="InterPro:IPR000680"
gene-bvRMA01 001129	lp30	gene	alpha	Pfam	protein matc	7	299	3.3E-69	+	.	Target=null 7 299;ID=match$1634_7_299;signature_desc=Borrelia lipoprotein;Name=PF00921;status=T;Dbxref="InterPro:IPR000680"
gene-bvRMA01 001130	lp30	gene	delta	Pfam	protein matc	39	163	1.1E-40	+	.	Target=null 39 163;ID=match$2249_39_163;signature_desc=Borrelia lipoprotein;Name=PF00921;status=T;Dbxref="InterPro:IPR000680"
gene-bvRMA01 001131	lp30	gene	gamma	Pfam	protein matc	34	309	2E-86	+	.	Target=null 34 309;ID=match$333_34_309;signature_desc=Borrelia lipoprotein;Name=PF00921;status=T;Dbxref="InterPro:IPR000680"
gene-bvRMA01 001132	lp30	gene	alpha	Pfam	protein matc	52	343	2E-70	+	.	Target=null 52 343;ID=match$997_52_343;signature_desc=Borrelia lipoprotein;Name=PF00921;status=T;Dbxref="InterPro:IPR000680"
gene-bvRMA01 001138	lp31	gene	beta	Pfam	protein matc	45	326	7.5E-45	+	.	Target=null 45 326;ID=match$2714_45_326;signature_desc=Borrelia lipoprotein;Name=PF00921;status=T;Dbxref="InterPro:IPR000680"
gene-bvRMA01 001139	lp31	gene	gamma	Pfam	protein matc	34	313	1.7E-87	+	.	Target=null 34 313;ID=match$1195_34_313;signature_desc=Borrelia lipoprotein;Name=PF00921;status=T;Dbxref="InterPro:IPR000680"
gene-bvRMA01 001140	lp31	gene	delta	Pfam	protein matc	45	306	5.5E-78	+	.	Target=null 45 306;ID=match$1081_45_306;signature_desc=Borrelia lipoprotein;Name=PF00921;status=T;Dbxref="InterPro:IPR000680"
gene-bvRMA01 001141	lp31	gene	beta	Pfam	protein matc	34	319	6.1E-46	+	.	Target=null 34 319;ID=match$2746_34_319;signature_desc=Borrelia lipoprotein;Name=PF00921;status=T;Dbxref="InterPro:IPR000680"
gene-bvRMA01 001143	lp31	gene	gamma	Pfam	protein matc	31	310	1.8E-78	+	.	Target=null 31 310;ID=match$2416_31_310;signature_desc=Borrelia lipoprotein;Name=PF00921;status=T;Dbxref="InterPro:IPR000680"
gene-bvRMA01 001159	lp31	pseudo	gamma	Pfam	protein matc	1	162	1E-34	+	.	Target=null 1 162;ID=match$1208_1_162;signature_desc=Borrelia lipoprotein;Name=PF00921;status=T;Dbxref="InterPro:IPR000680"
gene-bvRMA01 001159	lp31	pseudo		Pfam	protein matc	160	232	1.2E-25	+	.	Target=null 160 232;ID=match$1208_160_232;signature_desc=Borrelia lipoprotein;Name=PF00921;status=T;Dbxref="InterPro:IPR000680"
gene-bvRMA01 001161	lp31	pseudo	alpha	Pfam	protein matc	3	75	3.5E-10	+	.	Target=null 3 75;ID=match$42_3_75;signature_desc=Borrelia lipoprotein;Name=PF00921;status=T;Dbxref="InterPro:IPR000680"
gene-bvRMA01 001161	lp31	pseudo		Pfam	protein matc	113	168	2.8E-10	+	.	Target=null 113 168;ID=match$964_113_168;signature_desc=Borrelia lipoprotein;Name=PF00921;status=T;Dbxref="InterPro:IPR000680"
gene-bvRMA01 001161	lp31	pseudo		Pfam	protein matc	7	125	5.4E-15	+	.	Target=null 7 125;ID=match$964_7_125;signature_desc=Borrelia lipoprotein;Name=PF00921;status=T;Dbxref="InterPro:IPR000680"
gene-bvRMA01 001162	lp31	gene	beta	Pfam	protein matc	14	197	3E-14	+	.	Target=null 14 197;ID=match$2345_14_197;signature_desc=Borrelia lipoprotein;Name=PF00921;status=T;Dbxref="InterPro:IPR000680"
gene-bvRMA01 001162	lp31	gene		Pfam	protein matc	198	383	2.2E-29	+	.	Target=null 198 383;ID=match$2345_198_383;signature_desc=Borrelia lipoprotein;Name=PF00921;status=T;Dbxref="InterPro:IPR000680"
gene-bvRMA01 001165	lp31	gene	gamma	Pfam	protein matc	45	326	2.2E-90	+	.	Target=null 45 326;ID=match$545_45_326;signature_desc=Borrelia lipoprotein;Name=PF00921;status=T;Dbxref="InterPro:IPR000680"
gene-bvRMA01 001166	lp31	pseudo	alpha	Pfam	protein matc	58	272	3.8E-39	+	.	Target=null 58 272;ID=match$2557_58_272;signature_desc=Borrelia lipoprotein;Name=PF00921;status=T;Dbxref="InterPro:IPR000680"
gene-bvRMA01 001166	lp31	pseudo		Pfam	protein matc	13	95	7.6E-30	+	.	Target=null 13 95;ID=match$1449_13_95;signature_desc=Borrelia lipoprotein;Name=PF00921;status=T;Dbxref="InterPro:IPR000680"
gene-bvRMA01 001167	lp31	gene	beta	Pfam	protein matc	55	342	8.2E-48	+	.	Target=null 55 342;ID=match$657_55_342;signature_desc=Borrelia lipoprotein;Name=PF00921;status=T;Dbxref="InterPro:IPR000680"
gene-bvRMA01_001197	lp35	gene	gamma	Pfam	protein_matc	44	319	1.8E-85	+	.	Target=null 44 319;ID=match$6_44_319;signature_desc=Borrelia lipoprotein;Name=PF00921;status=T;Dbxref="InterPro:IPR000680"
gene-bvRMA01 001198	lp35	gene	beta	Pfam	protein matc	45	326	7.5E-45	+	.	Target=null 45 326;ID=match$2710_45_326;signature_desc=Borrelia lipoprotein;Name=PF00921;status=T;Dbxref="InterPro:IPR000680"
gene-bvRMA01 001229	lp37	gene	alpha	Pfam	protein matc	7	299	3.3E-69	+	.	Target=null 7 299;ID=match$1632_7_299;signature_desc=Borrelia lipoprotein;Name=PF00921;status=T;Dbxref="InterPro:IPR000680"
gene-bvRMA01 001230	lp37	gene	delta	Pfam	protein matc	39	163	1.1E-40	+	.	Target=null 39 163;ID=match$2253_39_163;signature_desc=Borrelia lipoprotein;Name=PF00921;status=T;Dbxref="InterPro:IPR000680"
gene-bvRMA01 001231	lp37	gene	gamma	Pfam	protein matc	34	309	2E-86	+	.	Target=null 34 309;ID=match$337_34_309;signature_desc=Borrelia lipoprotein;Name=PF00921;status=T;Dbxref="InterPro:IPR000680"
gene-bvRMA01 001232	lp37	gene	alpha	Pfam	protein matc	52	343	2E-70	+	.	Target=null 52 343;ID=match$1001_52_343;signature_desc=Borrelia lipoprotein;Name=PF00921;status=T;Dbxref="InterPro:IPR000680"

Vsp	replicon	gene/pseudo									
gene-bt91E135 001351	lp38	gene		Pfam	protein matc	39	211	4.9E-48	+	.	Target=null 39 211;ID=match$1758 39 211;signature desc=Lipoprotein;Name=PF01441;status=T;Dbxref="InterPro:IPR001800"
gene-bt91E135 001353	lp38	gene		Pfam	protein matc	29	194	1.2E-57	+	.	Target=null 29 194;ID=match$2858 29 194;signature desc=Lipoprotein;Name=PF01441;status=T;Dbxref="InterPro:IPR001800"
gene-bt91E135 001361	lp38	gene		Pfam	protein matc	30	196	2.4E-39	+	.	Target=null 30 196;ID=match$3081 30 196;signature desc=Lipoprotein;Name=PF01441;status=T;Dbxref="InterPro:IPR001800"
gene-bt91E135 001369	lp38	gene		Pfam	protein matc	2	170	2.4E-52	+	.	Target=null 2 170;ID=match$2165 2 170;signature desc=Lipoprotein;Name=PF01441;status=T;Dbxref="InterPro:IPR001800"
gene-bt91E135 001372	lp38	gene		Pfam	protein matc	38	206	9.4E-61	+	.	Target=null 38 206;ID=match$456 38 206;signature desc=Lipoprotein;Name=PF01441;status=T;Dbxref="InterPro:IPR001800"
gene-bt91E135 001407	lp40	gene		Pfam	protein matc	39	211	4.9E-48	+	.	Target=null 39 211;ID=match$1756 39 211;signature desc=Lipoprotein;Name=PF01441;status=T;Dbxref="InterPro:IPR001800"
gene-bt91E135 001429	lp43	pseudo		Pfam	protein matc	23	159	5.6E-51	+	.	Target=null 23 159;ID=match$1807 23 159;signature desc=Lipoprotein;Name=PF01441;status=T;Dbxref="InterPro:IPR001800"
gene-bt91E135 001442	lp43	gene		Pfam	protein matc	38	208	9.3E-50	+	.	Target=null 38 208;ID=match$2569 38 208;signature desc=Lipoprotein;Name=PF01441;status=T;Dbxref="InterPro:IPR001800"
gene-bt91E135 001447	lp43	gene		Pfam	protein matc	38	204	1.2E-56	+	.	Target=null 38 204;ID=match$568 38 204;signature desc=Lipoprotein;Name=PF01441;status=T;Dbxref="InterPro:IPR001800"
gene-bt91E135 001469	lp48	gene		Pfam	protein matc	38	205	2.2E-55	+	.	Target=null 38 205;ID=match$452 38 205;signature desc=Lipoprotein;Name=PF01441;status=T;Dbxref="InterPro:IPR001800"
gene-bt91E135 001473	lp48	gene		Pfam	protein matc	37	206	2.8E-54	+	.	Target=null 37 206;ID=match$2827 37 206;signature desc=Lipoprotein;Name=PF01441;status=T;Dbxref="InterPro:IPR001800"
gene-bt91E135 001475	lp48	gene		Pfam	protein matc	38	207	5.4E-53	+	.	Target=null 38 207;ID=match$453 38 207;signature desc=Lipoprotein;Name=PF01441;status=T;Dbxref="InterPro:IPR001800"
gene-bt91E135 001480	lp48	gene		Pfam	protein matc	38	206	1.1E-59	+	.	Target=null 38 206;ID=match$465 38 206;signature desc=Lipoprotein;Name=PF01441;status=T;Dbxref="InterPro:IPR001800"
gene-bt91E135 001483	lp48	gene		Pfam	protein matc	38	212	1.8E-47	+	.	Target=null 38 212;ID=match$1315 38 212;signature desc=Lipoprotein;Name=PF01441;status=T;Dbxref="InterPro:IPR001800"
gene-bt91E135 001525	lp53	gene		Pfam	protein matc	39	205	1.2E-56	+	.	Target=null 39 205;ID=match$508 39 205;signature desc=Lipoprotein;Name=PF01441;status=T;Dbxref="InterPro:IPR001800"
gene-bt91E135 001536	lp53	pseudo		Pfam	protein matc	29	106	4.4E-31	+	.	Target=null 29 106;ID=match$1622 29 106;signature desc=Lipoprotein;Name=PF01441;status=T;Dbxref="InterPro:IPR001800"
gene-bt91E135 001536	lp53	pseudo		Pfam	protein matc	3	92	1.6E-25	+	.	Target=null 3 92;ID=match$314 3 92;signature desc=Lipoprotein;Name=PF01441;status=T;Dbxref="InterPro:IPR001800"
gene-bt91E135 001538	lp53	gene		Pfam	protein matc	38	208	1.5E-47	+	.	Target=null 38 208;ID=match$1775 38 208;signature desc=Lipoprotein;Name=PF01441;status=T;Dbxref="InterPro:IPR001800"
gene-bt91E135 001542	lp53	gene		Pfam	protein matc	38	206	2.5E-58	+	.	Target=null 38 206;ID=match$1095 38 206;signature desc=Lipoprotein;Name=PF01441;status=T;Dbxref="InterPro:IPR001800"
Vlp			Subfamily								
gene-bt91E135 001241	lp19	gene	gamma	Pfam	protein matc	51	331	2.5E-88	+	.	Target=null 51 331;ID=match$2504 51 331;signature desc=Borrelia lipoprotein;Name=PF00921;status=T;Dbxref="InterPro:IPR000680"
gene-bt91E135 001242	lp19	gene	alpha	Pfam	protein matc	41	341	4.9E-68	+	.	Target=null 41 341;ID=match$2697 41 341;signature desc=Borrelia lipoprotein;Name=PF00921;status=T;Dbxref="InterPro:IPR000680"
gene-bt91E135_001243_	lp19	gene	beta	Pfam	protein_matc	20	304	2.1E-49	+	.	Target=null 20 304;ID=match$762_20_304;signature_desc=Borrelia lipoprotein;Name=PF00921;status=T;Dbxref="InterPro:IPR000680"
gene-bt91E135 001308	lp36	pseudo	beta	Pfam	protein matc	16	304	3.2E-47	+	.	Target=null 16 304;ID=match$1629 16 304;signature desc=Borrelia lipoprotein;Name=PF00921;status=T;Dbxref="InterPro:IPR000680"
gene-bt91E135 001309	lp36	gene	alpha	Pfam	protein matc	58	355	2.2E-71	+	.	Target=null 58 355;ID=match$2642 58 355;signature desc=Borrelia lipoprotein;Name=PF00921;status=T;Dbxref="InterPro:IPR000680"
gene-bt91E135 001310	lp36	gene	beta	Pfam	protein matc	20	309	2.4E-49	+	.	Target=null 20 309;ID=match$2371 20 309;signature desc=Borrelia lipoprotein;Name=PF00921;status=T;Dbxref="InterPro:IPR000680"
gene-bt91E135 001311	lp36	gene	alpha	Pfam	protein matc	57	345	1E-67	+	.	Target=null 57 345;ID=match$1838 57 345;signature desc=Borrelia lipoprotein;Name=PF00921;status=T;Dbxref="InterPro:IPR000680"
gene-bt91E135 001312	lp36	gene	alpha	Pfam	protein matc	57	352	9.3E-71	+	.	Target=null 57 352;ID=match$2543 57 352;signature desc=Borrelia lipoprotein;Name=PF00921;status=T;Dbxref="InterPro:IPR000680"
gene-bt91E135 001314	lp36	gene	beta	Pfam	protein matc	55	338	7.4E-47	+	.	Target=null 55 338;ID=match$1554 55 338;signature desc=Borrelia lipoprotein;Name=PF00921;status=T;Dbxref="InterPro:IPR000680"
gene-bt91E135 001315	lp36	pseudo	beta	Pfam	protein matc	23	314	7.4E-47	+	.	Target=null 23 314;ID=match$713 23 314;signature desc=Borrelia lipoprotein;Name=PF00921;status=T;Dbxref="InterPro:IPR000680"
gene-bt91E135 001316	lp36	gene	alpha	Pfam	protein matc	58	355	1.4E-69	+	.	Target=null 58 355;ID=match$2656 58 355;signature desc=Borrelia lipoprotein;Name=PF00921;status=T;Dbxref="InterPro:IPR000680"
gene-bt91E135 001319	lp36	gene	beta	Pfam	protein matc	20	302	2.1E-44	+	.	Target=null 20 302;ID=match$630 20 302;signature desc=Borrelia lipoprotein;Name=PF00921;status=T;Dbxref="InterPro:IPR000680"
gene-bt91E135 001320	lp36	gene	alpha	Pfam	protein matc	57	353	2E-67	+	.	Target=null 57 353;ID=match$2430 57 353;signature desc=Borrelia lipoprotein;Name=PF00921;status=T;Dbxref="InterPro:IPR000680"
gene-bt91E135 001323	lp36	gene	gamma	Pfam	protein matc	34	317	1.1E-87	+	.	Target=null 34 317;ID=match$2998 34 317;signature desc=Borrelia lipoprotein;Name=PF00921;status=T;Dbxref="InterPro:IPR000680"
gene-bt91E135 001326	lp36	gene	delta	Pfam	protein matc	44	328	5.4E-85	+	.	Target=null 44 328;ID=match$359 44 328;signature desc=Borrelia lipoprotein;Name=PF00921;status=T;Dbxref="InterPro:IPR000680"
gene-bt91E135 001331	lp36	gene	gamma	Pfam	protein matc	9	50	1.5E-10	+	.	Target=null 9 50;ID=match$1170 9 50;signature desc=Borrelia lipoprotein;Name=PF00921;status=T;Dbxref="InterPro:IPR000680"
gene-bt91E135 001336	lp36	pseudo	alpha	Pfam	protein matc	45	121	7.1E-14	+	.	Target=null 45 121;ID=match$1194 45 121;signature desc=Borrelia lipoprotein;Name=PF00921;status=T;Dbxref="InterPro:IPR000680"
gene-bt91E135 001336	lp36	pseudo		Pfam	protein matc	11	81	5.4E-07	+	.	Target=null 11 81;ID=match$2369 11 81;signature desc=Borrelia lipoprotein;Name=PF00921;status=T;Dbxref="InterPro:IPR000680"
gene-bt91E135 001336	lp36	pseudo		Pfam	protein matc	6	129	7.3E-32	+	.	Target=null 6 129;ID=match$2971 6 129;signature desc=Borrelia lipoprotein;Name=PF00921;status=T;Dbxref="InterPro:IPR000680"
gene-bt91E135 001338	lp36	pseudo	alpha	Pfam	protein matc	8	72	3.8E-13	+	.	Target=null 8 72;ID=match$2502 8 72;signature desc=Borrelia lipoprotein;Name=PF00921;status=T;Dbxref="InterPro:IPR000680"
gene-bt91E135 001338	lp36	pseudo		Pfam	protein matc	7	107	2.9E-16	+	.	Target=null 7 107;ID=match$1214 7 107;signature desc=Borrelia lipoprotein;Name=PF00921;status=T;Dbxref="InterPro:IPR000680"
gene-bt91E135 001339	lp36	pseudo	beta	Pfam	protein matc	2	40	0.000038	+	.	Target=null 2 40;ID=match$1906 2 40;signature desc=Borrelia lipoprotein;Name=PF00921;status=T;Dbxref="InterPro:IPR000680"
gene-bt91E135 001341	lp36	gene	beta	Pfam	protein matc	192	445	1.7E-29	+	.	Target=null 192 445;ID=match$2469 192 445;signature desc=Borrelia lipoprotein;Name=PF00921;status=T;Dbxref="InterPro:IPR000680"
gene-bt91E135 001345	lp38	gene	beta	Pfam	protein matc	136	257	3E-26	+	.	Target=null 136 257;ID=match$205 136 257;signature desc=Borrelia lipoprotein;Name=PF00921;status=T;Dbxref="InterPro:IPR000680"
gene-bt91E135 001345	lp38	gene		Pfam	protein matc	11	136	3.8E-30	+	.	Target=null 11 136;ID=match$205 11 136;signature desc=Borrelia lipoprotein;Name=PF00921;status=T;Dbxref="InterPro:IPR000680"
gene-bt91E135 001346	lp38	gene	delta	Pfam	protein matc	1	45	1.7E-08	+	.	Target=null 1 45;ID=match$652 1 45;signature desc=Borrelia lipoprotein;Name=PF00921;status=T;Dbxref="InterPro:IPR000680"
gene-bt91E135 001347	lp38	pseudo	alpha	Pfam	protein matc	57	253	1.8E-39	+	.	Target=null 57 253;ID=match$3074 57 253;signature desc=Borrelia lipoprotein;Name=PF00921;status=T;Dbxref="InterPro:IPR000680"
gene-bt91E135_001347_	lp38	pseudo		Pfam	protein_matc	7	106	3.2E-28	+	.	Target=null 7 106;ID=match$649_7_106;signature_desc=Borrelia lipoprotein;Name=PF00921;status=T;Dbxref="InterPro:IPR000680"
gene-bt91E135 001349	lp38	gene	beta	Pfam	protein matc	14	248	4.9E-26	+	.	Target=null 14 248;ID=match$7 14 248;signature desc=Borrelia lipoprotein;Name=PF00921;status=T;Dbxref="InterPro:IPR000680"
gene-bt91E135 001349	lp38	gene		Pfam	protein matc	236	277	0.0000018	+	.	Target=null 236 277;ID=match$7 236 277;signature desc=Borrelia lipoprotein;Name=PF00921;status=T;Dbxref="InterPro:IPR000680"
gene-bt91E135 001350	lp38	gene	alpha	Pfam	protein matc	7	306	2.2E-70	+	.	Target=null 7 306;ID=match$2643 7 306;signature desc=Borrelia lipoprotein;Name=PF00921;status=T;Dbxref="InterPro:IPR000680"
gene-bt91E135 001352	lp38	gene	delta	Pfam	protein matc	19	298	3.2E-85	+	.	Target=null 19 298;ID=match$1713 19 298;signature desc=Borrelia lipoprotein;Name=PF00921;status=T;Dbxref="InterPro:IPR000680"
gene-bt91E135 001354	lp38	gene	gamma	Pfam	protein matc	34	310	1.8E-78	+	.	Target=null 34 310;ID=match$1859 34 310;signature desc=Borrelia lipoprotein;Name=PF00921;status=T;Dbxref="InterPro:IPR000680"
gene-bt91E135 001356	lp38	gene	delta	Pfam	protein matc	14	81	1.4E-22	+	.	Target=null 14 81;ID=match$2259 14 81;signature desc=Borrelia lipoprotein;Name=PF00921;status=T;Dbxref="InterPro:IPR000680"
gene-bt91E135 001358	lp38	gene	beta	Pfam	protein matc	59	341	6.8E-46	+	.	Target=null 59 341;ID=match$795 59 341;signature desc=Borrelia lipoprotein;Name=PF00921;status=T;Dbxref="InterPro:IPR000680"
gene-bt91E135 001360	lp38	gene	delta	Pfam	protein matc	57	107	3.4E-16	+	.	Target=null 57 107;ID=match$2388 57 107;signature desc=Borrelia lipoprotein;Name=PF00921;status=T;Dbxref="InterPro:IPR000680"
gene-bt91E135 001360	lp38	gene		Pfam	protein matc	8	58	3.6E-14	+	.	Target=null 8 58;ID=match$2388 8 58;signature desc=Borrelia lipoprotein;Name=PF00921;status=T;Dbxref="InterPro:IPR000680"
gene-bt91E135 001362	lp38	gene	delta	Pfam	protein matc	44	325	5.2E-86	+	.	Target=null 44 325;ID=match$603 44 325;signature desc=Borrelia lipoprotein;Name=PF00921;status=T;Dbxref="InterPro:IPR000680"
gene-bt91E135 001365	lp38	gene	beta	Pfam	protein matc	20	307	2.4E-47	+	.	Target=null 20 307;ID=match$1334 20 307;signature desc=Borrelia lipoprotein;Name=PF00921;status=T;Dbxref="InterPro:IPR000680"
gene-bt91E135 001366	lp38	gene	alpha	Pfam	protein matc	14	55	1.9E-09	+	.	Target=null 14 55;ID=match$1656 14 55;signature desc=Borrelia lipoprotein;Name=PF00921;status=T;Dbxref="InterPro:IPR000680"
gene-bt91E135 001367	lp38	gene	delta	Pfam	protein matc	34	317	1.6E-82	+	.	Target=null 34 317;ID=match$2537 34 317;signature desc=Borrelia lipoprotein;Name=PF00921;status=T;Dbxref="InterPro:IPR000680"
gene-bt91E135 001368	lp38	gene	delta	Pfam	protein matc	8	292	7.3E-87	+	.	Target=null 8 292;ID=match$375 8 292;signature desc=Borrelia lipoprotein;Name=PF00921;status=T;Dbxref="InterPro:IPR000680"
gene-bt91E135 001370	lp38	gene	beta	Pfam	protein matc	14	297	2.1E-47	+	.	Target=null 14 297;ID=match$641 14 297;signature desc=Borrelia lipoprotein;Name=PF00921;status=T;Dbxref="InterPro:IPR000680"
gene-bt91E135 001371	lp38	gene	alpha	Pfam	protein matc	50	272	1.1E-36	+	.	Target=null 50 272;ID=match$1141 50 272;signature desc=Borrelia lipoprotein;Name=PF00921;status=T;Dbxref="InterPro:IPR000680"
gene-bt91E135 001371	lp38	gene		Pfam	protein matc	260	315	4.1E-09	+	.	Target=null 260 315;ID=match$1141 260 315;signature desc=Borrelia lipoprotein;Name=PF00921;status=T;Dbxref="InterPro:IPR000680"
gene-bt91E135 001373	lp38	pseudo	gamma	Pfam	protein matc	6	47	3E-11	+	.	Target=null 6 47;ID=match$1316 6 47;signature desc=Borrelia lipoprotein;Name=PF00921;status=T;Dbxref="InterPro:IPR000680"
gene-bt91E135 001378	lp38	gene	delta	Pfam	protein matc	42	76	5.7E-09	+	.	Target=null 42 76;ID=match$2975 42 76;signature desc=Borrelia lipoprotein;Name=PF00921;status=T;Dbxref="InterPro:IPR000680"
gene-bt91E135 001408	lp40	gene	alpha	Pfam	protein matc	7	306	2.2E-70	+	.	Target=null 7 306;ID=match$2645 7 306;signature desc=Borrelia lipoprotein;Name=PF00921;status=T;Dbxref="InterPro:IPR000680"
gene-bt91E135 001409	lp40	gene	beta	Pfam	protein matc	14	248	4.9E-26	+	.	Target=null 14 248;ID=match$5 14 248;signature desc=Borrelia lipoprotein;Name=PF00921;status=T;Dbxref="InterPro:IPR000680"
gene-bt91E135 001409	lp40	gene		Pfam	protein matc	236	277	0.0000018	+	.	Target=null 236 277;ID=match$5 236 277;signature desc=Borrelia lipoprotein;Name=PF00921;status=T;Dbxref="InterPro:IPR000680"
gene-bt91E135 001412	lp40	gene	alpha	Pfam	protein matc	57	355	6.2E-72	+	.	Target=null 57 355;ID=match$1957 57 355;signature desc=Borrelia lipoprotein;Name=PF00921;status=T;Dbxref="InterPro:IPR000680"
gene-bt91E135 001413	lp40	gene	delta	Pfam	protein matc	1	45	1.7E-08	+	.	Target=null 1 45;ID=match$650 1 45;signature desc=Borrelia lipoprotein;Name=PF00921;status=T;Dbxref="InterPro:IPR000680"
gene-bt91E135_001414_	lp40	gene	beta	Pfam	protein_matc	136	257	3E-26	+	.	Target=null 136 257;ID=match$208_136_257;signature_desc=Borrelia lipoprotein;Name=PF00921;status=T;Dbxref="InterPro:IPR000680"
gene-bt91E135 001414	lp40	gene		Pfam	protein matc	11	136	3.8E-30	+	.	Target=null 11 136;ID=match$208 11 136;signature desc=Borrelia lipoprotein;Name=PF00921;status=T;Dbxref="InterPro:IPR000680"
gene-bt91E135 001422	lp43	gene	beta	Pfam	protein matc	14	299	2.1E-47	+	.	Target=null 14 299;ID=match$834 14 299;signature desc=Borrelia lipoprotein;Name=PF00921;status=T;Dbxref="InterPro:IPR000680"
gene-bt91E135 001423	lp43	gene	alpha	Pfam	protein matc	49	217	1.2E-24	+	.	Target=null 49 217;ID=match$1220 49 217;signature desc=Borrelia lipoprotein;Name=PF00921;status=T;Dbxref="InterPro:IPR000680"
gene-bt91E135 001423	lp43	gene		Pfam	protein matc	216	250	0.0000065	+	.	Target=null 216 250;ID=match$1220 216 250;signature desc=Borrelia lipoprotein;Name=PF00921;status=T;Dbxref="InterPro:IPR000680"
gene-bt91E135 001424	lp43	gene	delta	Pfam	protein matc	44	328	3.6E-85	+	.	Target=null 44 328;ID=match$1191 44 328;signature desc=Borrelia lipoprotein;Name=PF00921;status=T;Dbxref="InterPro:IPR000680"
gene-bt91E135 001425	lp43	gene	gamma	Pfam	protein matc	34	322	3.4E-93	+	.	Target=null 34 322;ID=match$969 34 322;signature desc=Borrelia lipoprotein;Name=PF00921;status=T;Dbxref="InterPro:IPR000680"
gene-bt91E135 001428	lp43	gene	delta	Pfam	protein matc	34	316	5.6E-90	+	.	Target=null 34 316;ID=match$1399 34 316;signature desc=Borrelia lipoprotein;Name=PF00921;status=T;Dbxref="InterPro:IPR000680"
gene-bt91E135 001429	lp43	pseudo	gamma	Pfam	protein matc	10	86	6E-25	+	.	Target=null 10 86;ID=match$42 10 86;signature desc=Borrelia lipoprotein;Name=PF00921;status=T;Dbxref="InterPro:IPR000680"
gene-bt91E135 001440	lp43	gene	alpha	Pfam	protein matc	51	344	4.5E-67	+	.	Target=null 51 344;ID=match$1591 51 344;signature desc=Borrelia lipoprotein;Name=PF00921;status=T;Dbxref="InterPro:IPR000680"
gene-bt91E135 001441	lp43	gene	beta	Pfam	protein matc	20	306	2.4E-45	+	.	Target=null 20 306;ID=match$2769 20 306;signature desc=Borrelia lipoprotein;Name=PF00921;status=T;Dbxref="InterPro:IPR000680"
gene-bt91E135 001443	lp43	gene	delta	Pfam	protein matc	8	292	1.9E-87	+	.	Target=null 8 292;ID=match$2837 8 292;signature desc=Borrelia lipoprotein;Name=PF00921;status=T;Dbxref="InterPro:IPR000680"
gene-bt91E135 001444	lp43	gene	gamma	Pfam	protein matc	34	319	6.2E-85	+	.	Target=null 34 319;ID=match$1512 34 319;signature desc=Borrelia lipoprotein;Name=PF00921;status=T;Dbxref="InterPro:IPR000680"
gene-bt91E135 001446	lp43	gene	delta	Pfam	protein matc	34	318	1.7E-86	+	.	Target=null 34 318;ID=match$2852 34 318;signature desc=Borrelia lipoprotein;Name=PF00921;status=T;Dbxref="InterPro:IPR000680"
gene-bt91E135 001448	lp43	gene	delta	Pfam	protein matc	48	327	2.9E-86	+	.	Target=null 48 327;ID=match$116 48 327;signature desc=Borrelia lipoprotein;Name=PF00921;status=T;Dbxref="InterPro:IPR000680"
gene-bt91E135 001449	lp43	gene	delta	Pfam	protein matc	34	315	7.4E-84	+	.	Target=null 34 315;ID=match$2460 34 315;signature desc=Borrelia lipoprotein;Name=PF00921;status=T;Dbxref="InterPro:IPR000680"
gene-bt91E135 001452	lp43	gene	alpha	Pfam	protein matc	57	342	8.8E-64	+	.	Target=null 57 342;ID=match$1379 57 342;signature desc=Borrelia lipoprotein;Name=PF00921;status=T;Dbxref="InterPro:IPR000680"
gene-bt91E135 001457	lp43	pseudo	delta	Pfam	protein matc	34	244	2.7E-53	+	.	Target=null 34 244;ID=match$1983 34 244;signature desc=Borrelia lipoprotein;Name=PF00921;status=T;Dbxref="InterPro:IPR000680"
gene-bt91E135 001457	lp43	pseudo		Pfam	protein matc	1	67	2.2E-27	+	.	Target=null 1 67;ID=match$2260 1 67;signature desc=Borrelia lipoprotein;Name=PF00921;status=T;Dbxref="InterPro:IPR000680"
gene-bt91E135 001458	lp43	gene	alpha	Pfam	protein matc	3	41	1.2E-11	+	.	Target=null 3 41;ID=match$1729 3 41;signature desc=Borrelia lipoprotein;Name=PF00921;status=T;Dbxref="InterPro:IPR000680"
gene-bt91E135 001459	lp43	pseudo	beta	Pfam	protein matc	14	246	2.3E-29	+	.	Target=null 14 246;ID=match$2320 14 246;signature desc=Borrelia lipoprotein;Name=PF00921;status=T;Dbxref="InterPro:IPR000680"
gene-bt91E135 001459	lp43	pseudo		Pfam	protein matc	4	60	9E-16	+	.	Target=null 4 60;ID=match$3032 4 60;signature desc=Borrelia lipoprotein;Name=PF00921;status=T;Dbxref="InterPro:IPR000680"
gene-bt91E135 001468	lp48	pseudo	delta	Pfam	protein matc	4	52	0.000064	+	.	Target=null 4 52;ID=match$1624 4 52;signature desc=Borrelia lipoprotein;Name=PF00921;status=T;Dbxref="InterPro:IPR000680"
gene-bt91E135 001468	lp48	pseudo		Pfam	protein matc	8	100	1.4E-27	+	.	Target=null 8 100;ID=match$113 8 100;signature desc=Borrelia lipoprotein;Name=PF00921;status=T;Dbxref="InterPro:IPR000680"
gene-bt91E135 001472	lp48	gene	gamma	Pfam	protein matc	1	282	2.5E-85	+	.	Target=null 1 282;ID=match$2909 1 282;signature desc=Borrelia lipoprotein;Name=PF00921;status=T;Dbxref="InterPro:IPR000680"
gene-bt91E135_001474_	lp48	gene	beta	Pfam	protein_matc	20	305	2.3E-44	+	.	Target=null 20 305;ID=match$808_20_305;signature_desc=Borrelia lipoprotein;Name=PF00921;status=T;Dbxref="InterPro:IPR000680"
gene-bt91E135 001476	lp48	gene	delta	Pfam	protein matc	34	310	7.6E-87	+	.	Target=null 34 310;ID=match$2589 34 310;signature desc=Borrelia lipoprotein;Name=PF00921;status=T;Dbxref="InterPro:IPR000680"
gene-bt91E135 001478	lp48	gene	delta	Pfam	protein matc	34	314	1.2E-85	+	.	Target=null 34 314;ID=match$2897 34 314;signature desc=Borrelia lipoprotein;Name=PF00921;status=T;Dbxref="InterPro:IPR000680"
gene-bt91E135 001479	lp48	gene	gamma	Pfam	protein matc	1	276	6.5E-79	+	.	Target=null 1 276;ID=match$1911 1 276;signature desc=Borrelia lipoprotein;Name=PF00921;status=T;Dbxref="InterPro:IPR000680"
gene-bt91E135 001482	lp48	gene	delta	Pfam	protein matc	147	189	5E-12	+	.	Target=null 147 189;ID=match$867 147 189;signature desc=Borrelia lipoprotein;Name=PF00921;status=T;Dbxref="InterPro:IPR000680"
gene-bt91E135 001482	lp48	gene		Pfam	protein matc	22	151	7.5E-32	+	.	Target=null 22 151;ID=match$867 22 151;signature desc=Borrelia lipoprotein;Name=PF00921;status=T;Dbxref="InterPro:IPR000680"
gene-bt91E135 001484	lp48	gene	beta	Pfam	protein matc	20	309	3.1E-47	+	.	Target=null 20 309;ID=match$1921 20 309;signature desc=Borrelia lipoprotein;Name=PF00921;status=T;Dbxref="InterPro:IPR000680"
gene-bt91E135 001485	lp48	gene	alpha	Pfam	protein matc	57	345	1.6E-67	+	.	Target=null 57 345;ID=match$2886 57 345;signature desc=Borrelia lipoprotein;Name=PF00921;status=T;Dbxref="InterPro:IPR000680"
gene-bt91E135 001486	lp48	gene	delta	Pfam	protein matc	44	327	1.2E-76	+	.	Target=null 44 327;ID=match$2648 44 327;signature desc=Borrelia lipoprotein;Name=PF00921;status=T;Dbxref="InterPro:IPR000680"
gene-bt91E135 001487	lp48	gene	gamma	Pfam	protein matc	44	330	1.1E-90	+	.	Target=null 44 330;ID=match$1504 44 330;signature desc=Borrelia lipoprotein;Name=PF00921;status=T;Dbxref="InterPro:IPR000680"
gene-bt91E135 001506	lp48	gene	alpha	Pfam	protein matc	58	354	9.4E-70	+	.	Target=null 58 354;ID=match$1350 58 354;signature desc=Borrelia lipoprotein;Name=PF00921;status=T;Dbxref="InterPro:IPR000680"
gene-bt91E135 001507	lp48	pseudo	beta	Pfam	protein matc	55	136	1.3E-08	+	.	Target=null 55 136;ID=match$2468 55 136;signature desc=Borrelia lipoprotein;Name=PF00921;status=T;Dbxref="InterPro:IPR000680"
gene-bt91E135 001507	lp48	pseudo		Pfam	protein matc	29	129	9.1E-24	+	.	Target=null 29 129;ID=match$256 29 129;signature desc=Borrelia lipoprotein;Name=PF00921;status=T;Dbxref="InterPro:IPR000680"
gene-bt91E135 001513	lp53	pseudo	beta	Pfam	protein matc	14	246	2.3E-29	+	.	Target=null 14 246;ID=match$2318 14 246;signature desc=Borrelia lipoprotein;Name=PF00921;status=T;Dbxref="InterPro:IPR000680"
gene-bt91E135 001513	lp53	pseudo		Pfam	protein matc	4	60	9E-16	+	.	Target=null 4 60;ID=match$3034 4 60;signature desc=Borrelia lipoprotein;Name=PF00921;status=T;Dbxref="InterPro:IPR000680"
gene-bt91E135 001514	lp53	gene	alpha	Pfam	protein matc	3	41	1.2E-11	+	.	Target=null 3 41;ID=match$1731 3 41;signature desc=Borrelia lipoprotein;Name=PF00921;status=T;Dbxref="InterPro:IPR000680"
gene-bt91E135 001515	lp53	pseudo	delta	Pfam	protein matc	34	244	2.7E-53	+	.	Target=null 34 244;ID=match$1981 34 244;signature desc=Borrelia lipoprotein;Name=PF00921;status=T;Dbxref="InterPro:IPR000680"
gene-bt91E135 001515	lp53	pseudo		Pfam	protein matc	5	53	8.1E-16	+	.	Target=null 5 53;ID=match$606 5 53;signature desc=Borrelia lipoprotein;Name=PF00921;status=T;Dbxref="InterPro:IPR000680"
gene-bt91E135 001520	lp53	gene	alpha	Pfam	protein matc	57	342	8.8E-64	+	.	Target=null 57 342;ID=match$1381 57 342;signature desc=Borrelia lipoprotein;Name=PF00921;status=T;Dbxref="InterPro:IPR000680"
gene-bt91E135 001523	lp53	gene	delta	Pfam	protein matc	34	315	7.4E-84	+	.	Target=null 34 315;ID=match$2462 34 315;signature desc=Borrelia lipoprotein;Name=PF00921;status=T;Dbxref="InterPro:IPR000680"
gene-bt91E135 001524	lp53	gene	delta	Pfam	protein matc	48	327	2.9E-86	+	.	Target=null 48 327;ID=match$118 48 327;signature desc=Borrelia lipoprotein;Name=PF00921;status=T;Dbxref="InterPro:IPR000680"
gene-bt91E135 001526	lp53	pseudo	delta	Pfam	protein matc	6	47	4.6E-10	+	.	Target=null 6 47;ID=match$79 6 47;signature desc=Borrelia lipoprotein;Name=PF00921;status=T;Dbxref="InterPro:IPR000680"
gene-bt91E135 001527	lp53	gene	delta	Pfam	protein matc	4	31	0.0000033	+	.	Target=null 4 31;ID=match$371 4 31;signature desc=Borrelia lipoprotein;Name=PF00921;status=T;Dbxref="InterPro:IPR000680"
gene-bt91E135 001528	lp53	gene	alpha	Pfam	protein matc	58	353	9.1E-68	+	.	Target=null 58 353;ID=match$3091 58 353;signature desc=Borrelia lipoprotein;Name=PF00921;status=T;Dbxref="InterPro:IPR000680"
gene-bt91E135 001531	lp53	gene	beta	Pfam	protein matc	48	330	2.5E-45	+	.	Target=null 48 330;ID=match$335 48 330;signature desc=Borrelia lipoprotein;Name=PF00921;status=T;Dbxref="InterPro:IPR000680"
gene-bt91E135_001534_	lp53	gene	beta	Pfam	protein_matc	48	336	9.3E-47	+	.	Target=null 48 336;ID=match$1550_48_336;signature_desc=Borrelia lipoprotein;Name=PF00921;status=T;Dbxref="InterPro:IPR000680"
gene-bt91E135 001535	lp53	gene	delta	Pfam	protein matc	19	298	4.8E-88	+	.	Target=null 19 298;ID=match$866 19 298;signature desc=Borrelia lipoprotein;Name=PF00921;status=T;Dbxref="InterPro:IPR000680"
gene-bt91E135 001537	lp53	gene	gamma	Pfam	protein matc	1	267	1.9E-72	+	.	Target=null 1 267;ID=match$1486 1 267;signature desc=Borrelia lipoprotein;Name=PF00921;status=T;Dbxref="InterPro:IPR000680"
gene-bt91E135 001539	lp53	gene	delta	Pfam	protein matc	44	335	1.8E-86	+	.	Target=null 44 335;ID=match$1069 44 335;signature desc=Borrelia lipoprotein;Name=PF00921;status=T;Dbxref="InterPro:IPR000680"
gene-bt91E135 001541	lp53	gene	delta	Pfam	protein matc	31	317	6.5E-88	+	.	Target=null 31 317;ID=match$622 31 317;signature desc=Borrelia lipoprotein;Name=PF00921;status=T;Dbxref="InterPro:IPR000680"
gene-bt91E135 001545	lp53	gene	delta	Pfam	protein matc	44	329	6.6E-88	+	.	Target=null 44 329;ID=match$1681 44 329;signature desc=Borrelia lipoprotein;Name=PF00921;status=T;Dbxref="InterPro:IPR000680"
gene-bt91E135 001556	lp53	gene	gamma	Pfam	protein matc	1	281	1.2E-77	+	.	Target=null 1 281;ID=match$2289 1 281;signature desc=Borrelia lipoprotein;Name=PF00921;status=T;Dbxref="InterPro:IPR000680"
gene-bt91E135 001560	lp53	gene	delta	Pfam	protein matc	34	313	5.5E-85	+	.	Target=null 34 313;ID=match$2488 34 313;signature desc=Borrelia lipoprotein;Name=PF00921;status=T;Dbxref="InterPro:IPR000680"
gene-bt91E135 001562	lp53	gene	alpha	Pfam	protein matc	58	351	4.7E-66	+	.	Target=null 58 351;ID=match$1583 58 351;signature desc=Borrelia lipoprotein;Name=PF00921;status=T;Dbxref="InterPro:IPR000680"
gene-bt91E135 001563	lp53	gene	beta	Pfam	protein matc	20	205	1.5E-22	+	.	Target=null 20 205;ID=match$15 20 205;signature desc=Borrelia lipoprotein;Name=PF00921;status=T;Dbxref="InterPro:IPR000680"

Vsp	replicon	gene/pseudo									
gene-btBTE5EL 001273	lp28	gene		Pfam	protein matc	38	205	1.9E-58	+	.	Target=null 38 205;ID=match$1317_38_205;signature_desc=Lipoprotein;Name=PF01441;status=T;Dbxref="InterPro:IPR001800"
gene-btBTE5EL 001281	lp28	gene		Pfam	protein matc	38	206	4.4E-64	+	.	Target=null 38 206;ID=match$3074_38_206;signature_desc=Lipoprotein;Name=PF01441;status=T;Dbxref="InterPro:IPR001800"
gene-btBTE5EL 001283	lp28	gene		Pfam	protein matc	23	190	2.3E-58	+	.	Target=null 23 190;ID=match$23_23_190;signature_desc=Lipoprotein;Name=PF01441;status=T;Dbxref="InterPro:IPR001800"
gene-btBTE5EL 001333	lp32	gene		Pfam	protein matc	38	206	4E-66	+	.	Target=null 38 206;ID=match$3062_38_206;signature_desc=Lipoprotein;Name=PF01441;status=T;Dbxref="InterPro:IPR001800"
gene-btBTE5EL 001336	lp32	gene		Pfam	protein matc	38	206	2.7E-53	+	.	Target=null 38 206;ID=match$585_38_206;signature_desc=Lipoprotein;Name=PF01441;status=T;Dbxref="InterPro:IPR001800"
gene-btBTE5EL 001338	lp32	gene		Pfam	protein matc	22	190	4.8E-62	+	.	Target=null 22 190;ID=match$1879_22_190;signature_desc=Lipoprotein;Name=PF01441;status=T;Dbxref="InterPro:IPR001800"
gene-btBTE5EL 001374	lp34	pseudo		Pfam	protein matc	27	78	1E-21	+	.	Target=null 27 78;ID=match$1346_27_78;signature_desc=Lipoprotein;Name=PF01441;status=T;Dbxref="InterPro:IPR001800"
gene-btBTE5EL 001376	lp34	gene		Pfam	protein matc	39	210	3.3E-50	+	.	Target=null 39 210;ID=match$1524_39_210;signature_desc=Lipoprotein;Name=PF01441;status=T;Dbxref="InterPro:IPR001800"
gene-btBTE5EL 001396	lp34	gene		Pfam	protein matc	1	167	4.1E-60	+	.	Target=null 1 167;ID=match$458_1_167;signature_desc=Lipoprotein;Name=PF01441;status=T;Dbxref="InterPro:IPR001800"
gene-btBTE5EL 001410	lp37	gene		Pfam	protein matc	28	195	1.1E-61	+	.	Target=null 28 195;ID=match$1079_28_195;signature_desc=Lipoprotein;Name=PF01441;status=T;Dbxref="InterPro:IPR001800"
gene-btBTE5EL 001430	lp37	gene		Pfam	protein matc	28	195	1.1E-61	+	.	Target=null 28 195;ID=match$1081_28_195;signature_desc=Lipoprotein;Name=PF01441;status=T;Dbxref="InterPro:IPR001800"
gene-btBTE5EL 001446	lp42	gene		Pfam	protein matc	38	207	3.8E-53	+	.	Target=null 38 207;ID=match$1866_38_207;signature_desc=Lipoprotein;Name=PF01441;status=T;Dbxref="InterPro:IPR001800"
gene-btBTE5EL 001448	lp42	gene		Pfam	protein matc	38	207	1.7E-59	+	.	Target=null 38 207;ID=match$370_38_207;signature_desc=Lipoprotein;Name=PF01441;status=T;Dbxref="InterPro:IPR001800"
gene-btBTE5EL 001451	lp42	gene		Pfam	protein matc	23	188	2.2E-56	+	.	Target=null 23 188;ID=match$2853_23_188;signature_desc=Lipoprotein;Name=PF01441;status=T;Dbxref="InterPro:IPR001800"
gene-btBTE5EL 001458	lp42	pseudo		Pfam	protein matc	39	151	2E-39	+	.	Target=null 39 151;ID=match$2909_39_151;signature_desc=Lipoprotein;Name=PF01441;status=T;Dbxref="InterPro:IPR001800"
gene-btBTE5EL 001458	lp42	pseudo		Pfam	protein matc	2	39	0.00000018	+	.	Target=null 2 39;ID=match$1850_2_39;signature_desc=Lipoprotein;Name=PF01441;status=T;Dbxref="InterPro:IPR001800"
gene-btBTE5EL 001467	lp42	gene		Pfam	protein matc	38	206	1.6E-60	+	.	Target=null 38 206;ID=match$2402_38_206;signature_desc=Lipoprotein;Name=PF01441;status=T;Dbxref="InterPro:IPR001800"
gene-btBTE5EL 001508	lp45	gene		Pfam	protein matc	38	206	4.4E-64	+	.	Target=null 38 206;ID=match$3076_38_206;signature_desc=Lipoprotein;Name=PF01441;status=T;Dbxref="InterPro:IPR001800"
gene-btBTE5EL 001510	lp45	gene		Pfam	protein matc	23	190	2.3E-58	+	.	Target=null 23 190;ID=match$25_23_190;signature_desc=Lipoprotein;Name=PF01441;status=T;Dbxref="InterPro:IPR001800"
gene-btBTE5EL 001530	lp46	gene		Pfam	protein matc	38	201	2.7E-53	+	.	Target=null 38 201;ID=match$2589_38_201;signature_desc=Lipoprotein;Name=PF01441;status=T;Dbxref="InterPro:IPR001800"
gene-btBTE5EL 001580	lp49	gene		Pfam	protein matc	38	205	4E-64	+	.	Target=null 38 205;ID=match$742_38_205;signature_desc=Lipoprotein;Name=PF01441;status=T;Dbxref="InterPro:IPR001800"
gene-btBTE5EL 001599	lp49	gene		Pfam	protein matc	39	212	2.4E-47	+	.	Target=null 39 212;ID=match$1537_39_212;signature_desc=Lipoprotein;Name=PF01441;status=T;Dbxref="InterPro:IPR001800"
gene-btBTE5EL 001615	lp49	gene		Pfam	protein matc	37	206	2.2E-53	+	.	Target=null 37 206;ID=match$1062_37_206;signature_desc=Lipoprotein;Name=PF01441;status=T;Dbxref="InterPro:IPR001800"

Vlp			Subfamily								
gene-btBTE5EL 001272	lp28	pseudo	alpha	Pfam	protein matc	40	182	2.3E-24	+	.	Target=null 40 182;ID=match$1502_40_182;signature_desc=Borrelia lipoprotein;Name=PF00921;status=T;Dbxref="InterPro:IPR000680"
gene-btBTE5EL 001272	lp28	pseudo		Pfam	protein matc	4	143	1.4E-33	+	.	Target=null 4 143;ID=match$496_4_143;signature_desc=Borrelia lipoprotein;Name=PF00921;status=T;Dbxref="InterPro:IPR000680"
gene-btBTE5EL 001275	lp28	pseudo	gamma	Pfam	protein matc	3	42	0.000065	+	.	Target=null 3 42;ID=match$1708_3_42;signature_desc=Borrelia lipoprotein;Name=PF00921;status=T;Dbxref="InterPro:IPR000680"
gene-btBTE5EL 001275	lp28	pseudo		Pfam	protein matc	50	161	2.6E-33	+	.	Target=null 50 161;ID=match$330_50_161;signature_desc=Borrelia lipoprotein;Name=PF00921;status=T;Dbxref="InterPro:IPR000680"
gene-btBTE5EL 001282	lp28	gene	gamma	Pfam	protein matc	48	333	1.3E-88	+	.	Target=null 48 333;ID=match$1927_48_333;signature_desc=Borrelia lipoprotein;Name=PF00921;status=T;Dbxref="InterPro:IPR000680"
gene-btBTE5EL 001284	lp28	pseudo	delta	Pfam	protein matc	32	317	3E-83	+	.	Target=null 32 317;ID=match$1794_32_317;signature_desc=Borrelia lipoprotein;Name=PF00921;status=T;Dbxref="InterPro:IPR000680"
gene-btBTE5EL 001286	lp28	gene	delta	Pfam	protein matc	44	335	5E-87	+	.	Target=null 44 335;ID=match$1160_44_335;signature_desc=Borrelia lipoprotein;Name=PF00921;status=T;Dbxref="InterPro:IPR000680"
gene-btBTE5EL 001287	lp28	gene	beta	Pfam	protein matc	55	343	2.7E-47	+	.	Target=null 55 343;ID=match$2806_55_343;signature_desc=Borrelia lipoprotein;Name=PF00921;status=T;Dbxref="InterPro:IPR000680"
gene-btBTE5EL 001289	lp28	gene	alpha	Pfam	protein matc	59	343	1.3E-63	+	.	Target=null 59 343;ID=match$1714_59_343;signature_desc=Borrelia lipoprotein;Name=PF00921;status=T;Dbxref="InterPro:IPR000680"
gene-btBTE5EL 001290	lp28	gene	beta	Pfam	protein matc	14	297	2.9E-43	+	.	Target=null 14 297;ID=match$388_14_297;signature_desc=Borrelia lipoprotein;Name=PF00921;status=T;Dbxref="InterPro:IPR000680"
gene-btBTE5EL 001291	lp28	pseudo	gamma	Pfam	protein matc	34	115	6.4E-29	+	.	Target=null 34 115;ID=match$965_34_115;signature_desc=Borrelia lipoprotein;Name=PF00921;status=T;Dbxref="InterPro:IPR000680"
gene-btBTE5EL 001291	lp28	pseudo		Pfam	protein matc	4	203	2.8E-49	+	.	Target=null 4 203;ID=match$3079_4_203;signature_desc=Borrelia lipoprotein;Name=PF00921;status=T;Dbxref="InterPro:IPR000680"
gene-btBTE5EL 001298	lp30	gene	gamma	Pfam	protein matc	150	278	2.1E-23	+	.	Target=null 150 278;ID=match$3052_150_278;signature_desc=Borrelia lipoprotein;Name=PF00921;status=T;Dbxref="InterPro:IPR000680"
gene-btBTE5EL 001298	lp30	gene		Pfam	protein matc	6	138	5.1E-30	+	.	Target=null 6 138;ID=match$3052_6_138;signature_desc=Borrelia lipoprotein;Name=PF00921;status=T;Dbxref="InterPro:IPR000680"
gene-btBTE5EL 001301	lp30	gene	gamma	Pfam	protein matc	34	308	3.6E-77	+	.	Target=null 34 308;ID=match$3099_34_308;signature_desc=Borrelia lipoprotein;Name=PF00921;status=T;Dbxref="InterPro:IPR000680"
gene-btBTE5EL 001319	lp30	gene	gamma	Pfam	protein matc	1	276	3.1E-77	+	.	Target=null 1 276;ID=match$979_1_276;signature_desc=Borrelia lipoprotein;Name=PF00921;status=T;Dbxref="InterPro:IPR000680"
gene-btBTE5EL 001320	lp30	pseudo	beta	Pfam	protein matc	48	208	6.2E-16	+	.	Target=null 48 208;ID=match$596_48_208;signature_desc=Borrelia lipoprotein;Name=PF00921;status=T;Dbxref="InterPro:IPR000680"
gene-btBTE5EL 001320	lp30	pseudo		Pfam	protein matc	20	58	2.2E-09	+	.	Target=null 20 58;ID=match$1511_20_58;signature_desc=Borrelia lipoprotein;Name=PF00921;status=T;Dbxref="InterPro:IPR000680"
gene-btBTE5EL 001322	lp30	gene	delta	Pfam	protein matc	44	329	1.2E-87	+	.	Target=null 44 329;ID=match$808_44_329;signature_desc=Borrelia lipoprotein;Name=PF00921;status=T;Dbxref="InterPro:IPR000680"
gene-btBTE5EL 001323	lp30	gene	gamma	Pfam	protein matc	34	312	1.9E-84	+	.	Target=null 34 312;ID=match$326_34_312;signature_desc=Borrelia lipoprotein;Name=PF00921;status=T;Dbxref="InterPro:IPR000680"
gene-btBTE5EL 001325	lp30	gene	alpha	Pfam	protein matc	58	350	1.8E-74	+	.	Target=null 58 350;ID=match$2586_58_350;signature_desc=Borrelia lipoprotein;Name=PF00921;status=T;Dbxref="InterPro:IPR000680"
gene-btBTE5EL 001326	lp30	gene	beta	Pfam	protein matc	20	308	1.1E-50	+	.	Target=null 20 308;ID=match$2406_20_308;signature_desc=Borrelia lipoprotein;Name=PF00921;status=T;Dbxref="InterPro:IPR000680"
gene-btBTE5EL 001332	lp32	gene	beta	Pfam	protein matc	20	309	5.2E-48	+	.	Target=null 20 309;ID=match$1125_20_309;signature_desc=Borrelia lipoprotein;Name=PF00921;status=T;Dbxref="InterPro:IPR000680"
gene-btBTE5EL 001335	lp32	gene	delta	Pfam	protein matc	19	295	1.6E-84	+	.	Target=null 19 295;ID=match$1289_19_295;signature_desc=Borrelia lipoprotein;Name=PF00921;status=T;Dbxref="InterPro:IPR000680"
gene-btBTE5EL 001337	lp32	pseudo	delta	Pfam	protein matc	4	282	2.3E-82	+	.	Target=null 4 282;ID=match$2221_4_282;signature_desc=Borrelia lipoprotein;Name=PF00921;status=T;Dbxref="InterPro:IPR000680"
gene-btBTE5EL 001356	lp32	gene	gamma	Pfam	protein matc	1	278	1.2E-72	+	.	Target=null 1 278;ID=match$2044_1_278;signature_desc=Borrelia lipoprotein;Name=PF00921;status=T;Dbxref="InterPro:IPR000680"
gene-btBTE5EL 001358	lp32	gene	alpha	Pfam	protein matc	58	354	7.4E-65	+	.	Target=null 58 354;ID=match$2937_58_354;signature_desc=Borrelia lipoprotein;Name=PF00921;status=T;Dbxref="InterPro:IPR000680"
gene-btBTE5EL 001359	lp32	pseudo	beta	Pfam	protein matc	23	160	2.9E-16	+	.	Target=null 23 160;ID=match$1921_23_160;signature_desc=Borrelia lipoprotein;Name=PF00921;status=T;Dbxref="InterPro:IPR000680"
gene-btBTE5EL 001369	lp34	gene	delta	Pfam	protein matc	44	202	9.5E-37	+	.	Target=null 44 202;ID=match$2535_44_202;signature_desc=Borrelia lipoprotein;Name=PF00921;status=T;Dbxref="InterPro:IPR000680"
gene-btBTE5EL 001369	lp34	gene		Pfam	protein matc	210	290	3.4E-28	+	.	Target=null 210 290;ID=match$2535_210_290;signature_desc=Borrelia lipoprotein;Name=PF00921;status=T;Dbxref="InterPro:IPR000680"
gene-btBTE5EL 001370	lp34	gene	gamma	Pfam	protein matc	34	319	4.2E-87	+	.	Target=null 34 319;ID=match$1446_34_319;signature_desc=Borrelia lipoprotein;Name=PF00921;status=T;Dbxref="InterPro:IPR000680"
gene-btBTE5EL 001371	lp34	gene	delta	Pfam	protein matc	44	323	2.4E-82	+	.	Target=null 44 323;ID=match$468_44_323;signature_desc=Borrelia lipoprotein;Name=PF00921;status=T;Dbxref="InterPro:IPR000680"
gene-btBTE5EL 001373	lp34	gene	delta	Pfam	protein matc	30	121	1.5E-29	+	.	Target=null 30 121;ID=match$2620_30_121;signature_desc=Borrelia lipoprotein;Name=PF00921;status=T;Dbxref="InterPro:IPR000680"
gene-btBTE5EL 001375	lp34	gene	beta	Pfam	protein matc	20	307	1.7E-49	+	.	Target=null 20 307;ID=match$982_20_307;signature_desc=Borrelia lipoprotein;Name=PF00921;status=T;Dbxref="InterPro:IPR000680"
gene-btBTE5EL 001389	lp34	pseudo	gamma	Pfam	protein matc	1	90	2.1E-22	+	.	Target=null 1 90;ID=match$48_1_90;signature_desc=Borrelia lipoprotein;Name=PF00921;status=T;Dbxref="InterPro:IPR000680"
gene-btBTE5EL 001389	lp34	pseudo		Pfam	protein matc	26	208	9.2E-48	+	.	Target=null 26 208;ID=match$2828_26_208;signature_desc=Borrelia lipoprotein;Name=PF00921;status=T;Dbxref="InterPro:IPR000680"
gene-btBTE5EL 001390	lp34	gene	beta	Pfam	protein matc	38	322	4.3E-44	+	.	Target=null 38 322;ID=match$512_38_322;signature_desc=Borrelia lipoprotein;Name=PF00921;status=T;Dbxref="InterPro:IPR000680"
gene-btBTE5EL 001393	lp34	gene	alpha	Pfam	protein matc	53	348	2.3E-67	+	.	Target=null 53 348;ID=match$2532_53_348;signature_desc=Borrelia lipoprotein;Name=PF00921;status=T;Dbxref="InterPro:IPR000680"
gene-btBTE5EL 001394	lp34	pseudo	beta	Pfam	protein matc	14	261	2.1E-34	+	.	Target=null 14 261;ID=match$2171_14_261;signature_desc=Borrelia lipoprotein;Name=PF00921;status=T;Dbxref="InterPro:IPR000680"
gene-btBTE5EL 001394	lp34	pseudo		Pfam	protein matc	2	39	2.2E-10	+	.	Target=null 2 39;ID=match$2182_2_39;signature_desc=Borrelia lipoprotein;Name=PF00921;status=T;Dbxref="InterPro:IPR000680"
gene-btBTE5EL 001395	lp34	gene	delta	Pfam	protein matc	34	315	3.9E-88	+	.	Target=null 34 315;ID=match$2099_34_315;signature_desc=Borrelia lipoprotein;Name=PF00921;status=T;Dbxref="InterPro:IPR000680"
gene-btBTE5EL 001397	lp34	pseudo	delta	Pfam	protein matc	8	166	2.8E-38	+	.	Target=null 8 166;ID=match$184_8_166;signature_desc=Borrelia lipoprotein;Name=PF00921;status=T;Dbxref="InterPro:IPR000680"
gene-btBTE5EL 001397	lp34	pseudo		Pfam	protein matc	11	84	2E-28	+	.	Target=null 11 84;ID=match$247_11_84;signature_desc=Borrelia lipoprotein;Name=PF00921;status=T;Dbxref="InterPro:IPR000680"
gene-btBTE5EL 001402	lp37	gene	beta	Pfam	protein matc	171	423	1.2E-26	+	.	Target=null 171 423;ID=match$240_171_423;signature_desc=Borrelia lipoprotein;Name=PF00921;status=T;Dbxref="InterPro:IPR000680"
gene-btBTE5EL 001404	lp37	gene	delta	Pfam	protein matc	44	327	1.4E-84	+	.	Target=null 44 327;ID=match$1256_44_327;signature_desc=Borrelia lipoprotein;Name=PF00921;status=T;Dbxref="InterPro:IPR000680"
gene-btBTE5EL 001407	lp37	pseudo	beta	Pfam	protein matc	3	248	6.1E-31	+	.	Target=null 3 248;ID=match$798_3_248;signature_desc=Borrelia lipoprotein;Name=PF00921;status=T;Dbxref="InterPro:IPR000680"
gene-btBTE5EL 001408	lp37	gene	alpha	Pfam	protein matc	58	354	1.9E-70	+	.	Target=null 58 354;ID=match$3174_58_354;signature_desc=Borrelia lipoprotein;Name=PF00921;status=T;Dbxref="InterPro:IPR000680"
gene-btBTE5EL 001409	lp37	gene	delta	Pfam	protein matc	19	302	6.4E-88	+	.	Target=null 19 302;ID=match$2000_19_302;signature_desc=Borrelia lipoprotein;Name=PF00921;status=T;Dbxref="InterPro:IPR000680"
gene-btBTE5EL 001411	lp37	gene	beta	Pfam	protein matc	20	303	6.5E-43	+	.	Target=null 20 303;ID=match$268_20_303;signature_desc=Borrelia lipoprotein;Name=PF00921;status=T;Dbxref="InterPro:IPR000680"
gene-btBTE5EL 001412	lp37	gene	alpha	Pfam	protein matc	62	344	1.2E-64	+	.	Target=null 62 344;ID=match$1265_62_344;signature_desc=Borrelia lipoprotein;Name=PF00921;status=T;Dbxref="InterPro:IPR000680"
gene-btBTE5EL 001413	lp37	gene	gamma	Pfam	protein matc	1	274	2.8E-79	+	.	Target=null 1 274;ID=match$739_1_274;signature_desc=Borrelia lipoprotein;Name=PF00921;status=T;Dbxref="InterPro:IPR000680"
gene-btBTE5EL 001427	lp37	gene	gamma	Pfam	protein matc	1	274	2.8E-79	+	.	Target=null 1 274;ID=match$737_1_274;signature_desc=Borrelia lipoprotein;Name=PF00921;status=T;Dbxref="InterPro:IPR000680"
gene-btBTE5EL 001428	lp37	pseudo	alpha	Pfam	protein matc	48	330	1E-64	+	.	Target=null 48 330;ID=match$2292_48_330;signature_desc=Borrelia lipoprotein;Name=PF00921;status=T;Dbxref="InterPro:IPR000680"
gene-btBTE5EL 001429	lp37	gene	beta	Pfam	protein matc	20	303	6.5E-43	+	.	Target=null 20 303;ID=match$270_20_303;signature_desc=Borrelia lipoprotein;Name=PF00921;status=T;Dbxref="InterPro:IPR000680"
gene-btBTE5EL 001431	lp37	gene	delta	Pfam	protein matc	19	302	6.4E-88	+	.	Target=null 19 302;ID=match$1998_19_302;signature_desc=Borrelia lipoprotein;Name=PF00921;status=T;Dbxref="InterPro:IPR000680"
gene-btBTE5EL 001432	lp37	gene	alpha	Pfam	protein matc	58	354	1.9E-70	+	.	Target=null 58 354;ID=match$3176_58_354;signature_desc=Borrelia lipoprotein;Name=PF00921;status=T;Dbxref="InterPro:IPR000680"
gene-btBTE5EL 001433	lp37	pseudo	beta	Pfam	protein matc	3	248	6.1E-31	+	.	Target=null 3 248;ID=match$796_3_248;signature_desc=Borrelia lipoprotein;Name=PF00921;status=T;Dbxref="InterPro:IPR000680"
gene-btBTE5EL 001436	lp37	gene	delta	Pfam	protein matc	44	327	1.4E-84	+	.	Target=null 44 327;ID=match$1254_44_327;signature_desc=Borrelia lipoprotein;Name=PF00921;status=T;Dbxref="InterPro:IPR000680"
gene-btBTE5EL 001438	lp37	gene	beta	Pfam	protein matc	171	423	1.2E-26	+	.	Target=null 171 423;ID=match$242_171_423;signature_desc=Borrelia lipoprotein;Name=PF00921;status=T;Dbxref="InterPro:IPR000680"
gene-btBTE5EL 001443	lp42	pseudo	beta	Pfam	protein matc	16	304	3.2E-47	+	.	Target=null 16 304;ID=match$1358_16_304;signature_desc=Borrelia lipoprotein;Name=PF00921;status=T;Dbxref="InterPro:IPR000680"
gene-btBTE5EL 001444	lp42	gene	alpha	Pfam	protein matc	53	350	6.2E-72	+	.	Target=null 53 350;ID=match$432_53_350;signature_desc=Borrelia lipoprotein;Name=PF00921;status=T;Dbxref="InterPro:IPR000680"
gene-btBTE5EL 001445	lp42	gene	delta	Pfam	protein matc	31	315	1.8E-86	+	.	Target=null 31 315;ID=match$488_31_315;signature_desc=Borrelia lipoprotein;Name=PF00921;status=T;Dbxref="InterPro:IPR000680"
gene-btBTE5EL 001447	lp42	gene	gamma	Pfam	protein matc	1	277	2E-76	+	.	Target=null 1 277;ID=match$3206_1_277;signature_desc=Borrelia lipoprotein;Name=PF00921;status=T;Dbxref="InterPro:IPR000680"
gene-btBTE5EL 001449	lp42	gene	beta	Pfam	protein matc	20	306	3.9E-50	+	.	Target=null 20 306;ID=match$2781_20_306;signature_desc=Borrelia lipoprotein;Name=PF00921;status=T;Dbxref="InterPro:IPR000680"
gene-btBTE5EL 001450	lp42	gene	alpha	Pfam	protein matc	8	304	2.2E-57	+	.	Target=null 8 304;ID=match$2249_8_304;signature_desc=Borrelia lipoprotein;Name=PF00921;status=T;Dbxref="InterPro:IPR000680"
gene-btBTE5EL 001452	lp42	gene	delta	Pfam	protein matc	44	323	1E-83	+	.	Target=null 44 323;ID=match$3047_44_323;signature_desc=Borrelia lipoprotein;Name=PF00921;status=T;Dbxref="InterPro:IPR000680"
gene-btBTE5EL 001457	lp42	gene	alpha	Pfam	protein matc	7	134	3.7E-26	+	.	Target=null 7 134;ID=match$2251_7_134;signature_desc=Borrelia lipoprotein;Name=PF00921;status=T;Dbxref="InterPro:IPR000680"
gene-btBTE5EL 001459	lp42	gene	gamma	Pfam	protein matc	41	320	2.3E-85	+	.	Target=null 41 320;ID=match$2490_41_320;signature_desc=Borrelia lipoprotein;Name=PF00921;status=T;Dbxref="InterPro:IPR000680"
gene-btBTE5EL 001464	lp42	gene	gamma	Pfam	protein matc	1	279	5E-84	+	.	Target=null 1 279;ID=match$2890_1_279;signature_desc=Borrelia lipoprotein;Name=PF00921;status=T;Dbxref="InterPro:IPR000680"
gene-btBTE5EL 001465	lp42	gene	delta	Pfam	protein matc	44	329	7.1E-90	+	.	Target=null 44 329;ID=match$1164_44_329;signature_desc=Borrelia lipoprotein;Name=PF00921;status=T;Dbxref="InterPro:IPR000680"
gene-btBTE5EL 001468	lp42	gene	alpha	Pfam	protein matc	7	301	5.8E-65	+	.	Target=null 7 301;ID=match$1916_7_301;signature_desc=Borrelia lipoprotein;Name=PF00921;status=T;Dbxref="InterPro:IPR000680"
gene-btBTE5EL 001469	lp42	pseudo	beta	Pfam	protein matc	16	295	3.4E-44	+	.	Target=null 16 295;ID=match$3082_16_295;signature_desc=Borrelia lipoprotein;Name=PF00921;status=T;Dbxref="InterPro:IPR000680"
gene-btBTE5EL 001472	lp42	gene	alpha	Pfam	protein matc	62	346	1.3E-63	+	.	Target=null 62 346;ID=match$1057_62_346;signature_desc=Borrelia lipoprotein;Name=PF00921;status=T;Dbxref="InterPro:IPR000680"
gene-btBTE5EL 001473	lp42	gene	beta	Pfam	protein matc	14	297	4.9E-44	+	.	Target=null 14 297;ID=match$1936_14_297;signature_desc=Borrelia lipoprotein;Name=PF00921;status=T;Dbxref="InterPro:IPR000680"
gene-btBTE5EL 001474	lp42	gene	gamma	Pfam	protein matc	44	329	7.3E-85	+	.	Target=null 44 329;ID=match$548_44_329;signature_desc=Borrelia lipoprotein;Name=PF00921;status=T;Dbxref="InterPro:IPR000680"
gene-btBTE5EL 001509	lp45	gene	gamma	Pfam	protein matc	48	333	1.3E-88	+	.	Target=null 48 333;ID=match$1925_48_333;signature_desc=Borrelia lipoprotein;Name=PF00921;status=T;Dbxref="InterPro:IPR000680"
gene-btBTE5EL 001511	lp45	pseudo	delta	Pfam	protein matc	32	317	3E-83	+	.	Target=null 32 317;ID=match$1796_32_317;signature_desc=Borrelia lipoprotein;Name=PF00921;status=T;Dbxref="InterPro:IPR000680"
gene-btBTE5EL 001513	lp45	gene	delta	Pfam	protein matc	44	335	5E-87	+	.	Target=null 44 335;ID=match$1158_44_335;signature_desc=Borrelia lipoprotein;Name=PF00921;status=T;Dbxref="InterPro:IPR000680"
gene-btBTE5EL 001514	lp45	gene	beta	Pfam	protein matc	55	343	2.7E-47	+	.	Target=null 55 343;ID=match$2808_55_343;signature_desc=Borrelia lipoprotein;Name=PF00921;status=T;Dbxref="InterPro:IPR000680"
gene-btBTE5EL 001516	lp45	gene	alpha	Pfam	protein matc	59	343	1.3E-63	+	.	Target=null 59 343;ID=match$1716_59_343;signature_desc=Borrelia lipoprotein;Name=PF00921;status=T;Dbxref="InterPro:IPR000680"
gene-btBTE5EL 001517	lp45	gene	beta	Pfam	protein matc	14	297	2.9E-43	+	.	Target=null 14 297;ID=match$390_14_297;signature_desc=Borrelia lipoprotein;Name=PF00921;status=T;Dbxref="InterPro:IPR000680"
gene-btBTE5EL 001518	lp45	gene	gamma	Pfam	protein matc	34	313	2.5E-85	+	.	Target=null 34 313;ID=match$888_34_313;signature_desc=Borrelia lipoprotein;Name=PF00921;status=T;Dbxref="InterPro:IPR000680"
gene-btBTE5EL 001525	lp46	pseudo	delta	Pfam	protein matc	44	202	5.1E-38	+	.	Target=null 44 202;ID=match$2247_44_202;signature_desc=Borrelia lipoprotein;Name=PF00921;status=T;Dbxref="InterPro:IPR000680"
gene-btBTE5EL 001525	lp46	pseudo		Pfam	protein matc	11	84	2E-28	+	.	Target=null 11 84;ID=match$249_11_84;signature_desc=Borrelia lipoprotein;Name=PF00921;status=T;Dbxref="InterPro:IPR000680"
gene-btBTE5EL 001526	lp46	gene	gamma	Pfam	protein matc	34	319	4.2E-87	+	.	Target=null 34 319;ID=match$1448_34_319;signature_desc=Borrelia lipoprotein;Name=PF00921;status=T;Dbxref="InterPro:IPR000680"
gene-btBTE5EL 001527	lp46	pseudo	delta	Pfam	protein matc	44	184	7.7E-39	+	.	Target=null 44 184;ID=match$2495_44_184;signature_desc=Borrelia lipoprotein;Name=PF00921;status=T;Dbxref="InterPro:IPR000680"
gene-btBTE5EL 001527	lp46	pseudo		Pfam	protein matc	24	159	5.3E-40	+	.	Target=null 24 159;ID=match$1286_24_159;signature_desc=Borrelia lipoprotein;Name=PF00921;status=T;Dbxref="InterPro:IPR000680"
gene-btBTE5EL 001529	lp46	gene	delta	Pfam	protein matc	19	297	5E-87	+	.	Target=null 19 297;ID=match$1532_19_297;signature_desc=Borrelia lipoprotein;Name=PF00921;status=T;Dbxref="InterPro:IPR000680"
gene-btBTE5EL 001551	lp46	pseudo	alpha	Pfam	protein matc	62	343	2.3E-65	+	.	Target=null 62 343;ID=match$1333_62_343;signature_desc=Borrelia lipoprotein;Name=PF00921;status=T;Dbxref="InterPro:IPR000680"
gene-btBTE5EL 001560	lp46	pseudo	gamma	Pfam	protein matc	34	61	2.7E-10	+	.	Target=null 34 61;ID=match$306_34_61;signature_desc=Borrelia lipoprotein;Name=PF00921;status=T;Dbxref="InterPro:IPR000680"
gene-btBTE5EL 001560	lp46	pseudo		Pfam	protein matc	1	114	1.6E-23	+	.	Target=null 1 114;ID=match$1770_1_114;signature_desc=Borrelia lipoprotein;Name=PF00921;status=T;Dbxref="InterPro:IPR000680"
gene-btBTE5EL 001560	lp46	pseudo		Pfam	protein matc	117	192	2E-23	+	.	Target=null 117 192;ID=match$1770_117_192;signature_desc=Borrelia lipoprotein;Name=PF00921;status=T;Dbxref="InterPro:IPR000680"
gene-btBTE5EL 001573	lp49	pseudo	beta	Pfam	protein matc	36	164	5E-24	+	.	Target=null 36 164;ID=match$328_36_164;signature_desc=Borrelia lipoprotein;Name=PF00921;status=T;Dbxref="InterPro:IPR000680"
gene-btBTE5EL 001574	lp49	pseudo	delta	Pfam	protein matc	5	119	7.3E-26	+	.	Target=null 5 119;ID=match$2115_5_119;signature_desc=Borrelia lipoprotein;Name=PF00921;status=T;Dbxref="InterPro:IPR000680"
gene-btBTE5EL 001577	lp49	gene	beta	Pfam	protein matc	48	331	7.7E-44	+	.	Target=null 48 331;ID=match$3173_48_331;signature_desc=Borrelia lipoprotein;Name=PF00921;status=T;Dbxref="InterPro:IPR000680"
gene-btBTE5EL 001579	lp49	pseudo	gamma	Pfam	protein matc	1	103	3.1E-29	+	.	Target=null 1 103;ID=match$461_1_103;signature_desc=Borrelia lipoprotein;Name=PF00921;status=T;Dbxref="InterPro:IPR000680"
gene-btBTE5EL 001579	lp49	pseudo		Pfam	protein matc	33	205	7.8E-49	+	.	Target=null 33 205;ID=match$2277_33_205;signature_desc=Borrelia lipoprotein;Name=PF00921;status=T;Dbxref="InterPro:IPR000680"
gene-btBTE5EL 001591	lp49	gene	delta	Pfam	protein matc	44	323	1.9E-79	+	.	Target=null 44 323;ID=match$3081_44_323;signature_desc=Borrelia lipoprotein;Name=PF00921;status=T;Dbxref="InterPro:IPR000680"
gene-btBTE5EL 001593	lp49	gene	delta	Pfam	protein matc	34	315	2E-87	+	.	Target=null 34 315;ID=match$2416_34_315;signature_desc=Borrelia lipoprotein;Name=PF00921;status=T;Dbxref="InterPro:IPR000680"
gene-btBTE5EL 001594	lp49	gene	delta	Pfam	protein matc	44	323	2.9E-84	+	.	Target=null 44 323;ID=match$2876_44_323;signature_desc=Borrelia lipoprotein;Name=PF00921;status=T;Dbxref="InterPro:IPR000680"
gene-btBTE5EL 001597	lp49	gene	delta	Pfam	protein matc	44	332	1.4E-80	+	.	Target=null 44 332;ID=match$355_44_332;signature_desc=Borrelia lipoprotein;Name=PF00921;status=T;Dbxref="InterPro:IPR000680"
gene-btBTE5EL 001600	lp49	gene	delta	Pfam	protein matc	19	294	1.2E-83	+	.	Target=null 19 294;ID=match$899_19_294;signature_desc=Borrelia lipoprotein;Name=PF00921;status=T;Dbxref="InterPro:IPR000680"
gene-btBTE5EL 001603	lp49	gene	gamma	Pfam	protein matc	34	322	8.3E-94	+	.	Target=null 34 322;ID=match$680_34_322;signature_desc=Borrelia lipoprotein;Name=PF00921;status=T;Dbxref="InterPro:IPR000680"
gene-btBTE5EL 001604	lp49	gene	delta	Pfam	protein matc	44	311	1.1E-78	+	.	Target=null 44 311;ID=match$2920_44_311;signature_desc=Borrelia lipoprotein;Name=PF00921;status=T;Dbxref="InterPro:IPR000680"
gene-btBTE5EL 001607	lp49	gene	gamma	Pfam	protein matc	34	312	2.3E-89	+	.	Target=null 34 312;ID=match$2_34_312;signature_desc=Borrelia lipoprotein;Name=PF00921;status=T;Dbxref="InterPro:IPR000680"
gene-btBTE5EL 001608	lp49	gene	delta	Pfam	protein matc	44	325	6.3E-85	+	.	Target=null 44 325;ID=match$1934_44_325;signature_desc=Borrelia lipoprotein;Name=PF00921;status=T;Dbxref="InterPro:IPR000680"
gene-btBTE5EL 001611	lp49	gene	gamma	Pfam	protein matc	44	335	7.1E-85	+	.	Target=null 44 335;ID=match$119_44_335;signature_desc=Borrelia lipoprotein;Name=PF00921;status=T;Dbxref="InterPro:IPR000680"
gene-btBTE5EL 001612	lp49	gene	delta	Pfam	protein matc	44	334	2.8E-83	+	.	Target=null 44 334;ID=match$2350_44_334;signature_desc=Borrelia lipoprotein;Name=PF00921;status=T;Dbxref="InterPro:IPR000680"
gene-btBTE5EL 001613	lp49	pseudo	alpha	Pfam	protein matc	47	341	2.4E-64	+	.	Target=null 47 341;ID=match$366_47_341;signature_desc=Borrelia lipoprotein;Name=PF00921;status=T;Dbxref="InterPro:IPR000680"
gene-btBTE5EL 001614	lp49	pseudo	beta	Pfam	protein matc	16	296	1.3E-46	+	.	Target=null 16 296;ID=match$1095_16_296;signature_desc=Borrelia lipoprotein;Name=PF00921;status=T;Dbxref="InterPro:IPR000680"
gene-btBTE5EL 001616	lp49	gene	delta	Pfam	protein matc	8	292	2.8E-86	+	.	Target=null 8 292;ID=match$2580_8_292;signature_desc=Borrelia lipoprotein;Name=PF00921;status=T;Dbxref="InterPro:IPR000680"
gene-btBTE5EL 001617	lp49	gene	delta	Pfam	protein matc	44	325	4E-85	+	.	Target=null 44 325;ID=match$34_44_325;signature_desc=Borrelia lipoprotein;Name=PF00921;status=T;Dbxref="InterPro:IPR000680"
gene-btBTE5EL 001619	lp49	pseudo	alpha	Pfam	protein matc	41	183	2.3E-24	+	.	Target=null 41 183;ID=match$774_41_183;signature_desc=Borrelia lipoprotein;Name=PF00921;status=T;Dbxref="InterPro:IPR000680"
gene-btBTE5EL 001619	lp49	pseudo		Pfam	protein matc	4	143	1.4E-33	+	.	Target=null 4 143;ID=match$494_4_143;signature_desc=Borrelia lipoprotein;Name=PF00921;status=T;Dbxref="InterPro:IPR000680"
